# TriplatinNC and Biomolecules: Building Models Based on Non-covalent Interactions

**DOI:** 10.3389/fchem.2019.00307

**Published:** 2019-05-21

**Authors:** Nathália M. P. Rosa, Frederico Henrique do C. Ferreira, Nicholas P. Farrell, Luiz Antônio S. Costa

**Affiliations:** ^1^Núcleo de Estudos em Química Computacional, Departamento de Química, ICE, Universidade Federal de Juiz de Fora, Juiz de Fora, Brazil; ^2^Department of Chemistry, Virginia Commonwealth University, Richmond, VA, United States

**Keywords:** triplatin complexes, biomolecules, phosphate clamps, sulfate clamps, arginine-forks, mimetic model, DFT, ONIOM

## Abstract

The class of polynuclear platinum(II) compounds have demonstrated a great interest because their high activity against cancer cells. Among these new compounds, the TriplatinNC also called AH78, demonstrated surprising antitumor activity, in some cases equivalent to cisplatin. It is well-known that complex charge +8 favors interaction with DNA and other biomolecules non-covalently, through the hydrogen bonds with phosphate and sulfate groups present in these structures. The hydrogen atoms of the amine interact with the oxygen atoms of the phosphate and sulfate groups present in the DNA strand and heparan sulfate, respectively. These interactions can cause significant twists in double helix and inhibit the activity of these biomolecules. The present investigation is an attempt to provide a benchmark theoretical study about TriplatinNC. We have described the non-covalent interactions through small reliable mimetic models. The non-covalent interactions were also evaluated on larger models containing DNA fractions with six nitrogenous base pairs (CGCGAA) and fractions of the disaccharide that makes the HS evaluated by the hybrid QM/MM ONIOM methodology.

## Introduction

Cisplatin has been the most widely metal-based drug used for the treatment of cancer in almost four decades, but the efficacy of this drug has become more difficult because of acquired resistance and severe side effects such as nephrotoxicity, neurotoxicity, and hearing system damage (Wong and Giandomenico, 1999). Other platinum complexes have been approved for clinical use worldwide, such as carboplatin (second generation), oxaliplatin (third generation), and three compounds as nedaplatin, lobaplatin, and heptaplatin, which have been approved only in Japan, China, and South Korea, respectively (Barry and Sadler, 2013). Therefore, approaches that have a different cellular response from that of cisplatin have been the target of studies in several areas.

The searching for new compounds such as polynuclear platinum complexes (PPCs) that show anti-cancer activity have increase in recent years (Mangrum and Farrell, 2010; Prisecaru et al., 2014; Farrell, 2015; Qu et al., 2015). PPCs have shown great promise against cancer cells due to faster and more effective interactions with the DNA when compared to mononuclear complexes because they have more than one platinum core available to coordinate, exhibiting chemical and biological properties that differ significantly to cisplatin (Qu et al., 2004).

**Figure 1:**
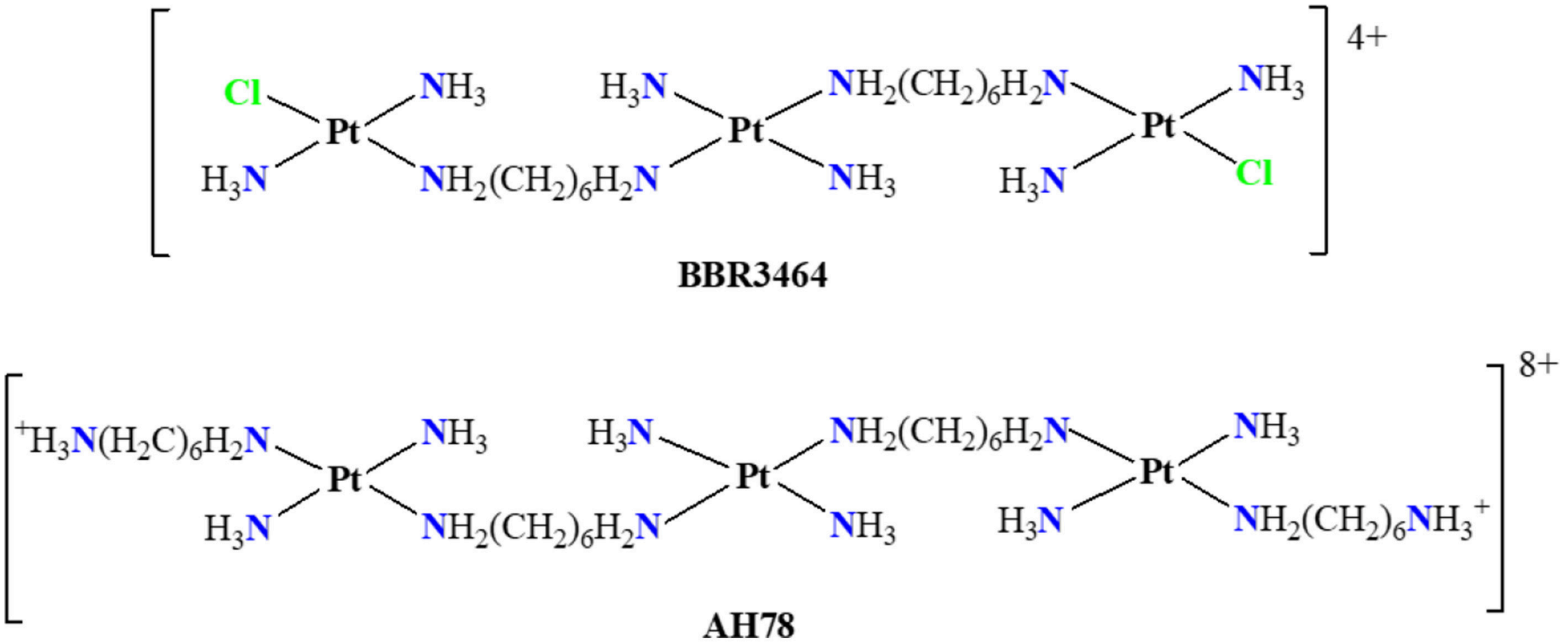
Planar structures of trinuclear platinum(II) complexes, Triplatin (BBR3464), and TriplatinNC (AH78).

The Triplatin complex BBR3464 (Figure 1) entered phase I of clinical trials in the 1990s and was the first multinuclear platinum compound to enter in phase II (Olszewski and Hamilton, 2010; Qu et al., 2015). Two of the three platinum centers in the Triplatin have chloride ligands, which allows the formation of two mono-functional adducts with DNA by long range cross-links (Manzotti et al., 2000; Boulikas and Vougiouka, 2003). The Triplatin (BBR3464) has demonstrated biological activity with cytotoxicity at concentrations in the micromolar order, similarly to cisplatin for some human cancer cell lines, they also have shown great potential to overcome the effects of drug resistance, and in many cases the solubility in water is appreciably the which makes it favorable for clinical practice (Riccardi et al., 2001; Wheate and Collins, 2005; Benedetti et al., 2011).

In principle, it was believed that platinum complexes should be neutral in order to exhibit anti-tumor activity. PPCs challenge this paradigm by presenting different forms of interaction with DNA. The TriplatinNC (Figure 1) complex shows overall charge +8, presenting cellular uptake about 5 times greater than its analog Triplatin, which has overall charge +4 and is comparable to that of cisplatin in some cell lines (Harris et al., 2005).

PPCs have different coordination modes to the DNA, inaccessible to mononuclear complexes; in mononuclear complexes short range connections, of type 1,2-intrastrand with DNA are predominant. Polynuclear platinum (II) complexes are capable of forming long-range coordination such as DNA inter- and intra-strand links (Komeda et al., 2010). These links can occur with distances up to 4 pairs of nitrogenous bases (Kasparkova et al., 2002). The charge created by the metal centers increases the binding kinetics with the DNA, because the connection is achieved by two coordination spheres of independent mono-functional platinum, eliminating many problems of steric hindrance which are present when two nucleobases are attached to one Pt atom, such as for cisplatin and its analogs. The charge present in the complex also impacts the extent and nature of the inter- and intra-strand crosslinks (Hegmans et al., 2004; Qu et al., 2015). Malina et al. have reported that the presence of the charged structure, as in the TriplatinNC, greatly can condense DNA and inhibit polymerase activity (Malina et al., 2014).

Differently from cisplatin, the trinuclear platinum (II) complexes shown in Figure 1 do not interact covalently with the DNA because they have no leaving groups, such as chloride present in the cisplatin. The observed non-covalent interactions are mainly of electrostatic type and the hydrogen bonds have an important role causing major distortions in the DNA double helix (Mangrum and Farrell, 2010). Komeda et al. (2006) report the crystal structure (PDB:2DYW) where the complex forms hydrogen bonds with the oxygen atoms of phosphate groups present in DNA (Mangrum and Farrell, 2010). These interactions occur between the hydrogen atoms of the amino groups of the complex and the oxygen atoms of the double helix backbone phosphates of DNA. Such interactions are known as *phosphate clamps* and may be backbone tracking or minor groove spanning. This term is used to indicate a cyclic structure in which one phosphate interacts with two hydrogen atoms from two a(m)mine ligands bound to a platinum core (Komeda et al., 2006; Wang and Gao, 2014). The exact mode depends on the specificity of base or helical local topological parameters of the oligonucleotide (Prisecaru et al., 2014).

Recent studies have shown another likely target for PPCs, glycosaminoglycans (GAGs), such as heparan sulfate (HS), which are linear polysaccharides composed of repeated disaccharide units of alternating residues of uronic acid and hexosamine. These molecules are directly related to the processes of angiogenesis and metastasis and when conjugated to proteins are found on the cell surface and extracellular matrix with critical functions in cell adhesion and migration. The TriplatinNC complex was shown to be capable of inhibiting the activity of these biomolecules by the formation of non-covalent interactions, through inhibition of Growth Factor Binding to HS, such as sulfate clamps, which are structures similar to those of phosphate clamps. The conformational similarity between HS and DNA justifies the choice of these targets for this study (Peterson et al., 2017; Katner et al., 2018).

Another possible structure is the phosphate or sulfate arginine-forks characterized by the formation of an eight-membered ring (including hydrogen atoms) stabilized by two hydrogen bonds between two a(m)mine groups at the *cis*-positions that interact with two oxygen atoms from the phosphate or sulfate. The PtN_4_ type of Platinum (II) centers have high selectivity for oxygen atoms present in biomolecules such as DNA and HS, being prone to the formation of these structures that have highly conserved geometry of binding (Komeda et al., 2010). Peterson and colleagues recently demonstrated that TriplatinNC is capable of blocking the interaction of different proteins with both DNA and HS (Peterson et al., 2014, 2017). The platinum (II) trinuclear complex inhibits the formation of the interaction between the TBP protein and DNA (Peterson et al., 2014).

The use of computational hybrid methods has been a very successful approach to enable more accurate performance on relatively large chemical systems combining quantum mechanics (QM) and molecular mechanics (MM) (Heerdt and Morgon, 2011). The already well-known method called ONIOM (Our Own n-layered Integrated Molecular Orbital and Molecular Mechanics), developed by Morokuma (2003), is a method that combines a variety of quantum methods with a multi-layered molecular mechanics method. The ONIOM method has been successfully applied in the study of interactions between DNA and metal complexes (Gkionis and Platts, 2012; Gkionis et al., 2013; Chung et al., 2015). These interactions, on several occasions, influence the cytotoxicity of the drug and can affect the DNA repair and other processes.

In recent years, although most of the theoretical studies of platinum drugs mostly involve the complex reaction mechanism with the purine bases of DNA, some theoretical studies have investigated on the PPC's reaction mechanism as anticancer drugs and their interaction with biological target. Chen and Zhou (2015) studied the mono-functional substitution reactions between cisplatin and PPCs, as Triplatin (BBR3464) with sulfur biomolecules and purine bases (guanine and adenine) in DNA with two different functional methods DFT-B3LYP/M06 by solvation model IEF-PCM. Recently, our group reported different works on platinum compounds involving the study of the hydrolysis process of a platinum dinuclear complex (Esteves et al., 2015), as well as inclusion complexes of binuclear platinum (II) complex (bisPt) into α-, β-, and γ-cyclodextrins via hydrogen interactions formed preferably by hydrogen atoms present in bisPt and the oxygen atoms from inside the cyclodextrin molecules (Paixão et al., 2012).

This study aims to describe the non-covalent interactions with TriplatinNC with DNA and HS complex through small reliable mimetic models, and by the use of DNA fractions containing six pairs of nitrogenous bases.

## Methodology

**Figure 2:**
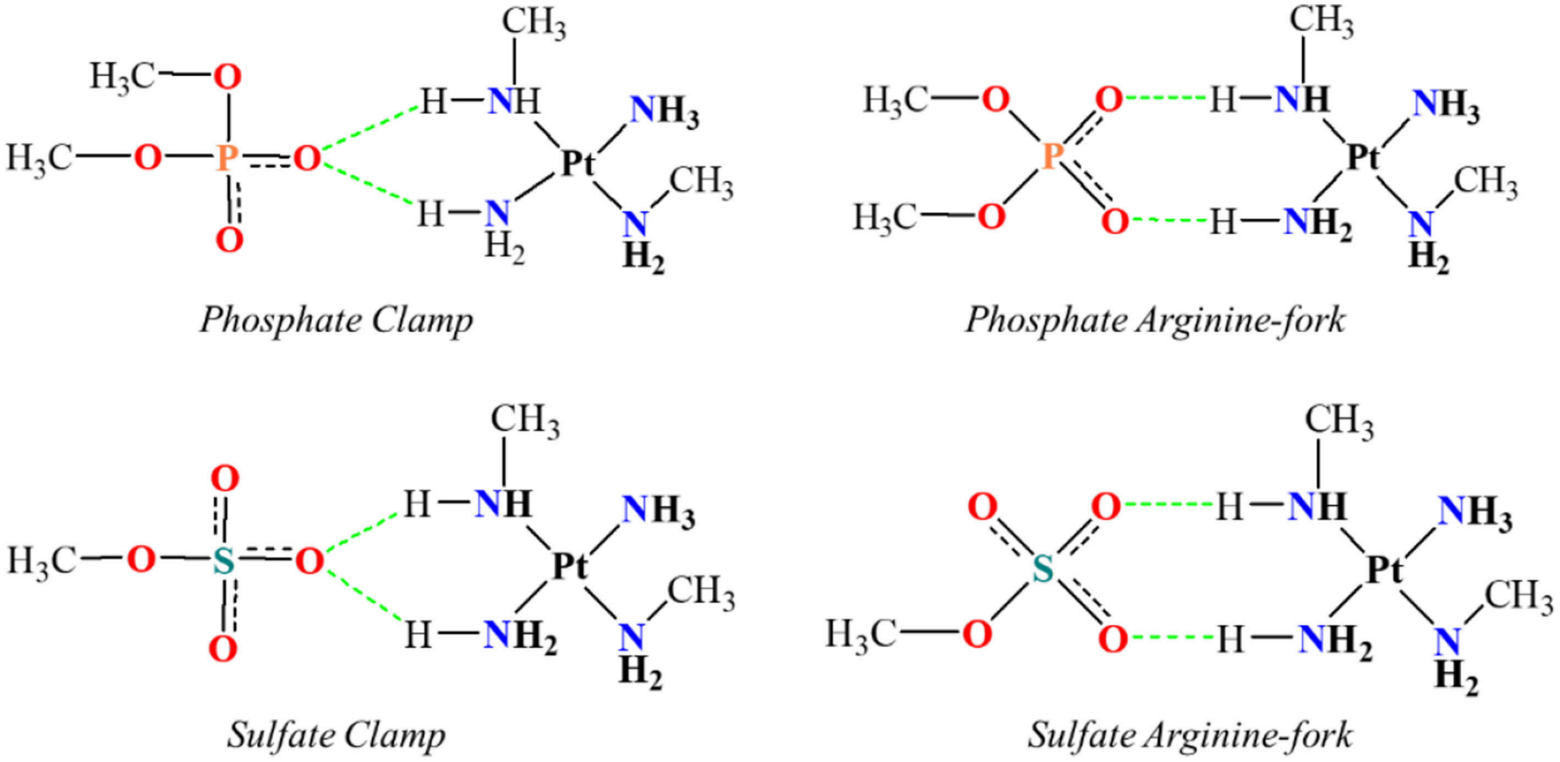
Mimetic models of phosphate and sulfate clamps and arginine-forks.

One of the problems in the study of many atoms systems is the “how” to diminish the size of the system while keeping the properties that might be of interest. The use of smaller chemical models that can represent the site of interest, in a feasible time, is an important strategy of study. The existing hydrogen interactions between the platinum centers and the DNA bases and HS were studied here by mimetic models. Initially, mimetic models were studied in order to evaluate the interaction energies for the structures of phosphate and sulfate clamps and arginine-forks, according to the models shown in Figure 2. These models were evaluated using the B3LYP (Becke, 1993a) and BHandH (Becke, 1993b) functional with the pseudopotential LANL2DZ (Hay and Wadt, 1985a,b,c) and SDD (Andrae et al., 1990), respectively for the platinum atom as well as the 6-31+G(d,p) (Here et al., 1972) basis set to the other atoms.

**Figure 3:**
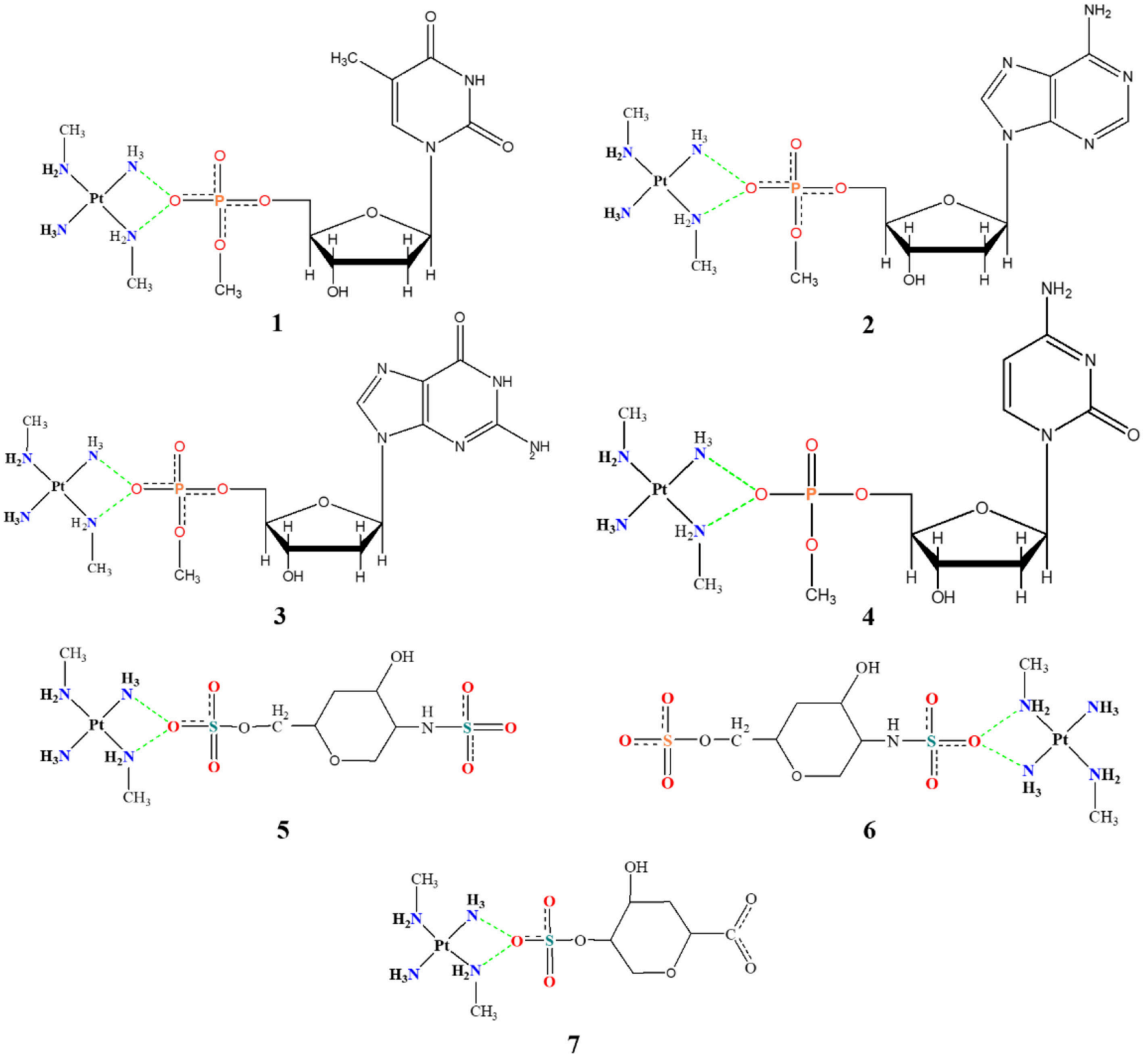
Mimetic models based on nitrogenous bases and fractions of the disaccharide.

Another seven models have been developed and are shown in Figure 3. Models **1**–**4** consist of the platinum coordination sphere and the nucleotide where the nitrogenous bases are thymine (T), adenine (A), guanine (G), and cytosine (C), respectively, and all atoms remained free. The models **5**–**7** consist of the platinum coordination sphere and fractions of the disaccharide that make the HS. These models were evaluated using the BHandH functional with the pseudopotential SDD for the platinum atom as well as the 6–31+G(d,p) basis set to the other atoms. All models have been optimized for clamps and arginine-forks structures. Natural Bond Orbital (NBO) calculations have also been performed at the very same level of theory described above.

**Figure 4:**
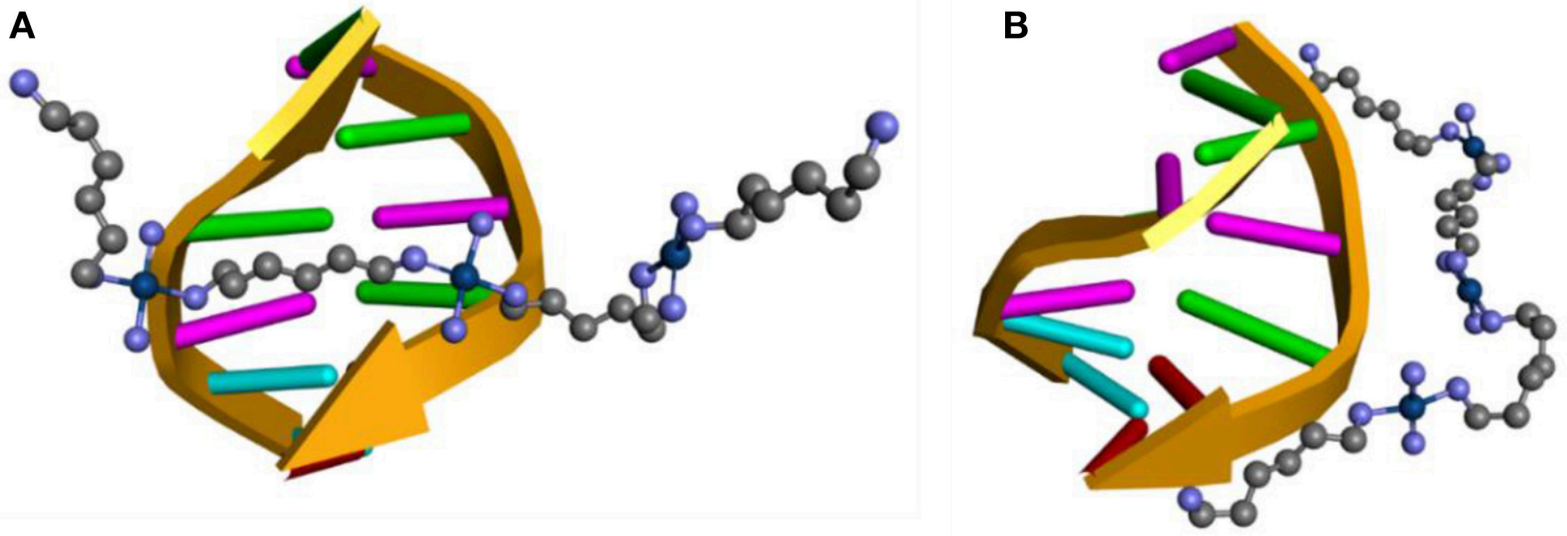
Scheme of the interaction position between the TriplatinNC complex with DNA: **(A)** Model A, **(B)** Model B.

The ONIOM hybrid method was used to evaluate the non-covalent interactions between a DNA fragment composed of 6 nitrogenous base pairs (CGCGAA) and the TriplatinNC complex arranged in two different ways, one with the complex spanning the two DNA strands, **Model A** (Figure 4A) and another with the complex placed along the same ribbon of the DNA fragment, **Model B** (Figure 4B). These structures were based on results reported by Komeda et al. (2010) and Prisecaru et al. (2014). The ONIOM method was also used to evaluate the interactions between the sulfate groups of a model of heparan sulfate formed by 3 units of the HS disaccharide, which corresponds to the half of the model reported in the PDB database, with 1HPN code and the TriplatinNC complex, **Model C** (Mulloy et al., 1993). The structures were obtained by defining for the upper layer the functional BHandH, with the set of basic functions 6–31+G(d,p) for the non-metallic atoms and SDD for the platinum atoms. For the low layer the force field UFF is being used (Rappé et al., 1992). Sodium atoms were added close to the phosphate groups that are not involved in the formation of phosphate clamps to decrease the loads of each layer. All the structures of the complexes were completely optimized using the IEFPCM model (Scalmani and Frisch, 2010) to include the solvent continuously, considering the molecule of the solute inserted in a medium of dielectric constant equal to that of water (ε = 78, 35^*^). All calculations, including NBO, were performed using the program package GAUSSIAN09 (Frisch et al., 2016).

## Results and Discussion

**Figure 5:**
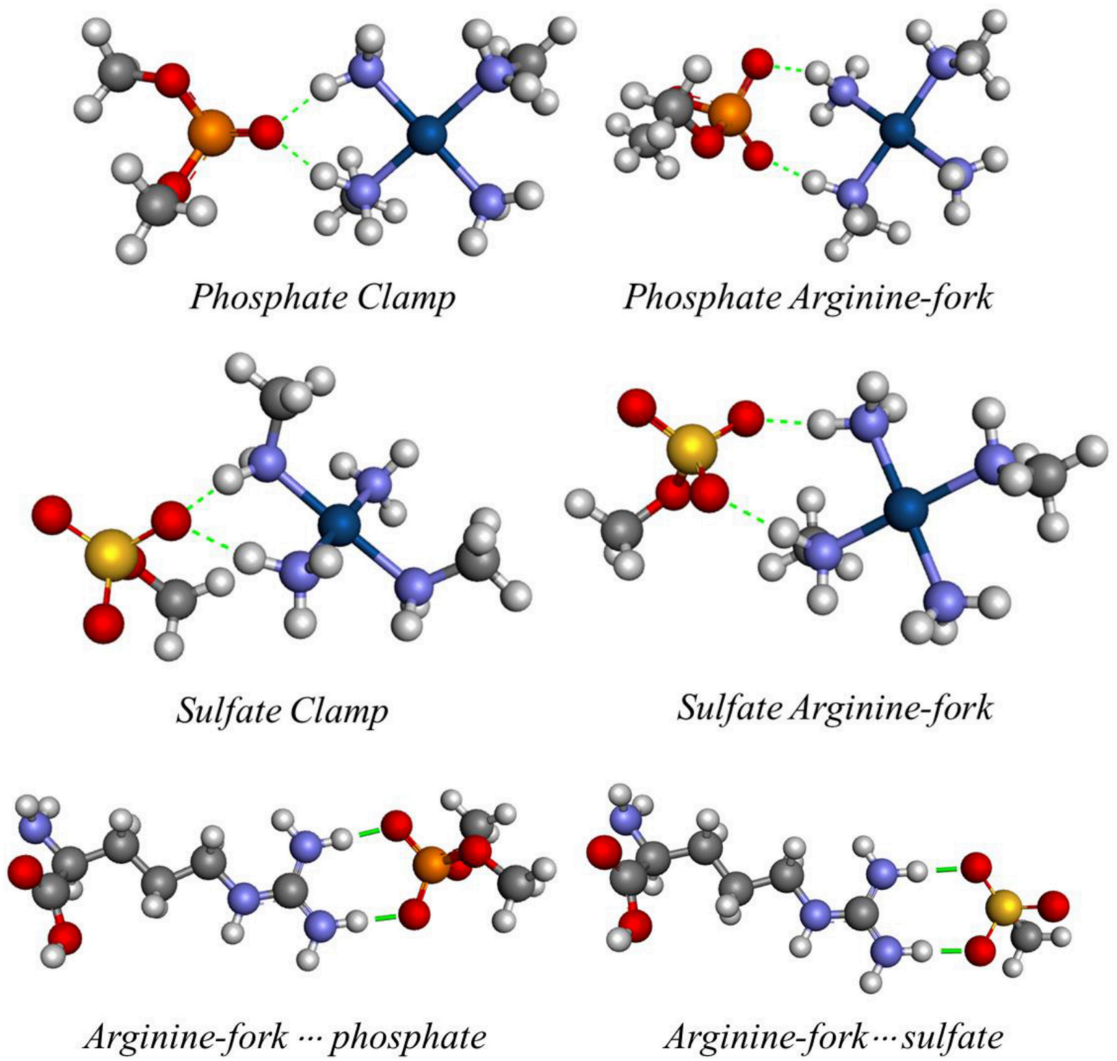
Optimized structures of the models using the BHandH functional.

Table 1 shows the values of interaction energies in kcal mol^−1^ for the models described in Figure 2. It is possible to observe that the structures obtained using the BHandH functional present the lowest free Gibbs energy values of interaction. Among these, it is noted that the arginine-fork structures present the most negative values, suggesting that such structures are more stable than the clamps, for the methodology studied. In order to compare, we analyzed a model that represents the arginine-forks composed of a phosphate group and the amino acid arginine (Figure 5). The Gibbs free energy values for the arginine-Forks are −7.88 and −7.38 kcal mol^−1^ for the phosphate and sulfate groups, respectively, contributing to the assertion that from the analyzed models the arginine-forks type structures can be more stable, from the point of view of the free energy of Gibbs for the methodology studied.

**Table 1:** Energy values of Gibbs free energy of interaction in kcal mol^−1^.

**ΔE_int_**	**B3LYP**		**BHandH**	
	**Phosphate**	**Sulfate**	**Phosphate**	**Sulfate**
Clamp	−2.28	3.45	−11.11	−6.12
Arginine-Fork	−3.63	2.06	−12.79	−8.29

Figure 5 shows the optimized structures obtained for the models analyzed with the respective hydrogen interactions among the hydrogen atoms from the amino groups of the platinum center with the oxygen atoms present in the DNA phosphate groups and HS sulfate groups using the BHandH functional.

**Figure 6:**
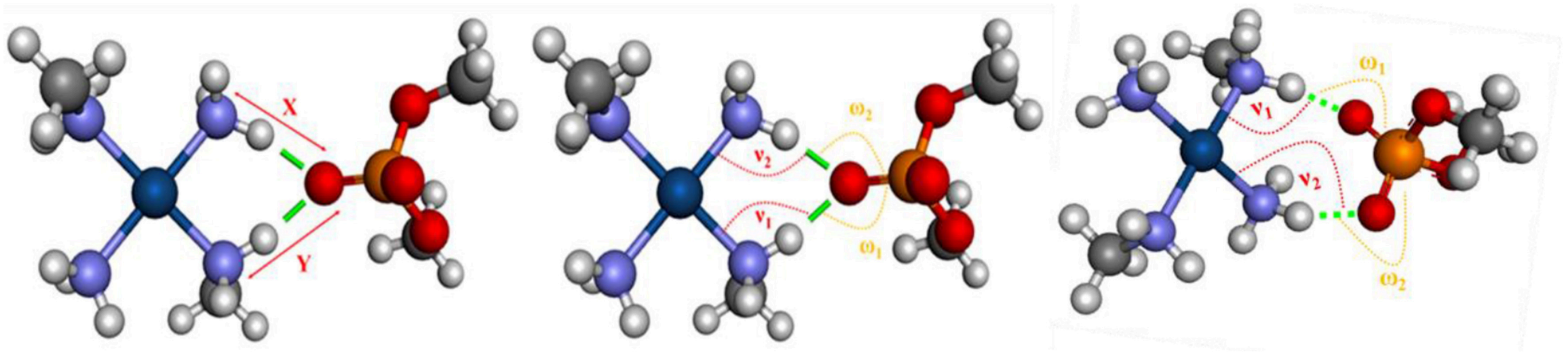
Bond distances and angles analyzed in models. Refer to Table 2 to see the values of x, y, ν_1_, ν_2_, ω_1_, and ω_2_.

According to Komeda et al. (2006), a phosphate clamp is a cyclic structure with a single oxygen atom of the phosphate group, which interacts via two hydrogen bonds from each of two amino ligands from a single Pt(II) center. It was observed that the formation of phosphate clamps seems to require the *cis* orientation of the nitrogenous ligands so that *trans* ligands do not participate in phosphate clamps, the mutually trans groups being too distant for the occurrence of interaction with a single phosphate group oxygen atom. Figure 6 shows the angles and distances analyzed and Table 2 presents the values obtained for the models of Figure 5.

**Table 2:** Distances (in Ångström) and bond angles (in degree) involved in the formation of clamps and arginine-forks using the BHandH functional.

		**Distances/Å**		**Angles/^°^**			
		**x**	**y**	**ν_1_**	**ν_2_**	**ω_1_**	**ω_2_**
Phosphate	Clamps	2.70	2.70	100.6	100.7	129.5	138.0
	Arginine-forks	2.74	2.69	103.4	111.7	127.0	96.3
Sulfate	Clamps	2.75	2.76	101.2	101.7	130.6	128.5
	Arginine-forks	2.78	2.78	112.4	118.2	108.1	123.7
Arginine-Forks	Phosphate	2.67	2.68	122.9	122.6	117.7	117.4
	Sulfate	2.74	2.75	120.4	121.6	121.1	120.5

The experimental values reported by Komeda et al. (2006) for x and y for the phosphate clamps are between 2.75 and 2.98 Å and between 2.83 and 3.22 Å, respectively. Thus, the values obtained for the mimetic models are close to the values reported in the literature. Considering that the structures formed by the sulfate groups are similar to those formed by the phosphate groups, it can be concluded that the values found for the binding distances for such structures are similar to the values reported in the literature to the phosphate clamps. For the analyzed angles, the experimental values for ν_1_ vary between 99 and 106° and for ν_2_ between 88 and 109°, from these data it was observed that the values obtained were similar to the experimental data. For the angles ω_1_ the values found are close to the experimental data that are between 113 and 145°, but for the angle ω_2_ it was observed that some values are below the experimentally reported interval, where the experimental values are between 128 and 151°, however for the sulfate clamp, angle ω_2_ is very close to that reported in the literature for phosphate clamps. Comparing with the results obtained for the arginine-forks with the phosphate and sulfate groups, it is noted that the values for the distances are close to the values obtained with the non-covalent structures formed by the centers of platinum (II). With respect to the angles, υ_1_ and υ_2_ are smaller when formed between the center of platinum and the phosphate and sulfate groups, already the angles ω_1_ and ω_2_ are mostly larger when compared to the results obtained for the arginine-forks.

**Figure 7:**
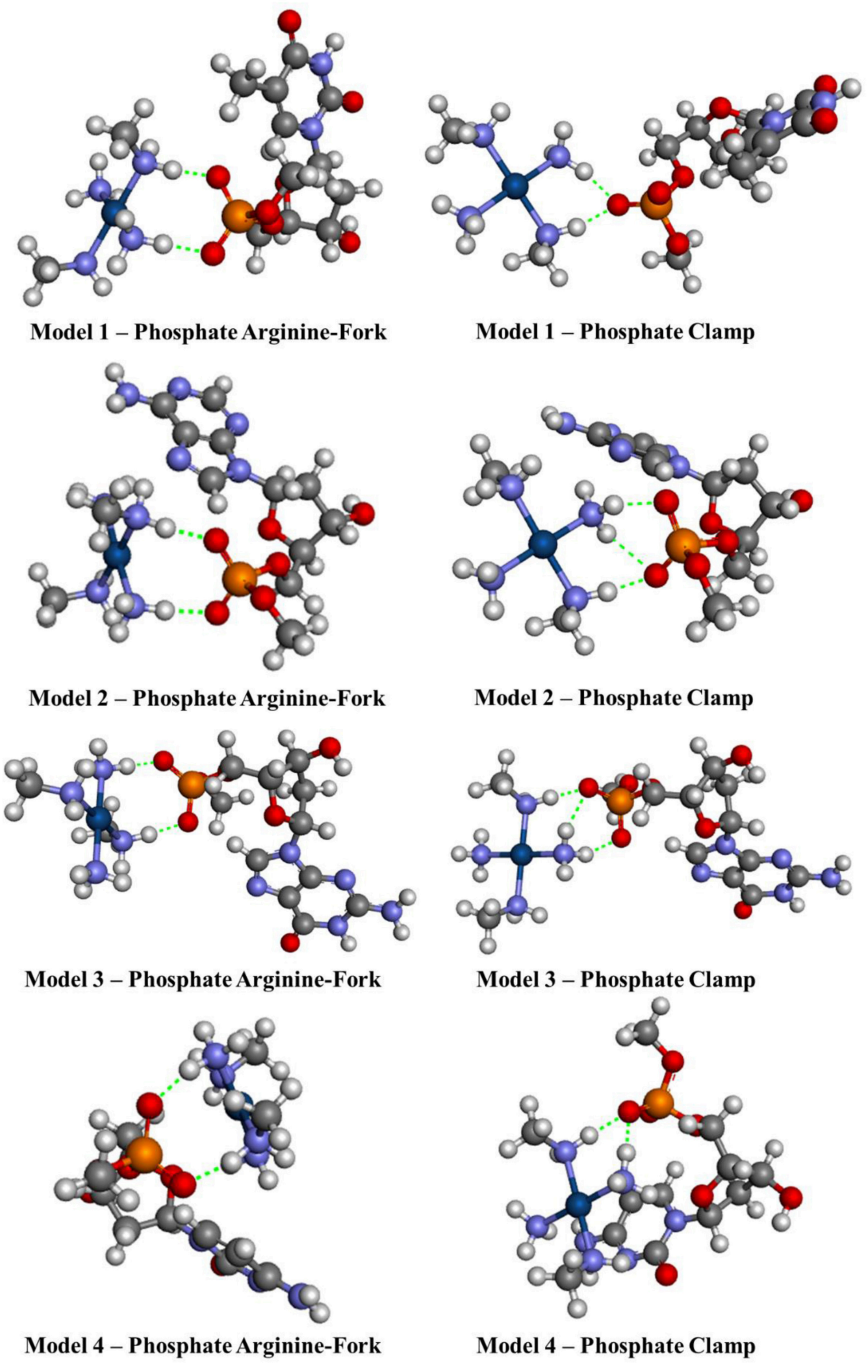
Optimized structures of each different phosphate models 1–4 using the BHandH functional.

**Figure 8:**
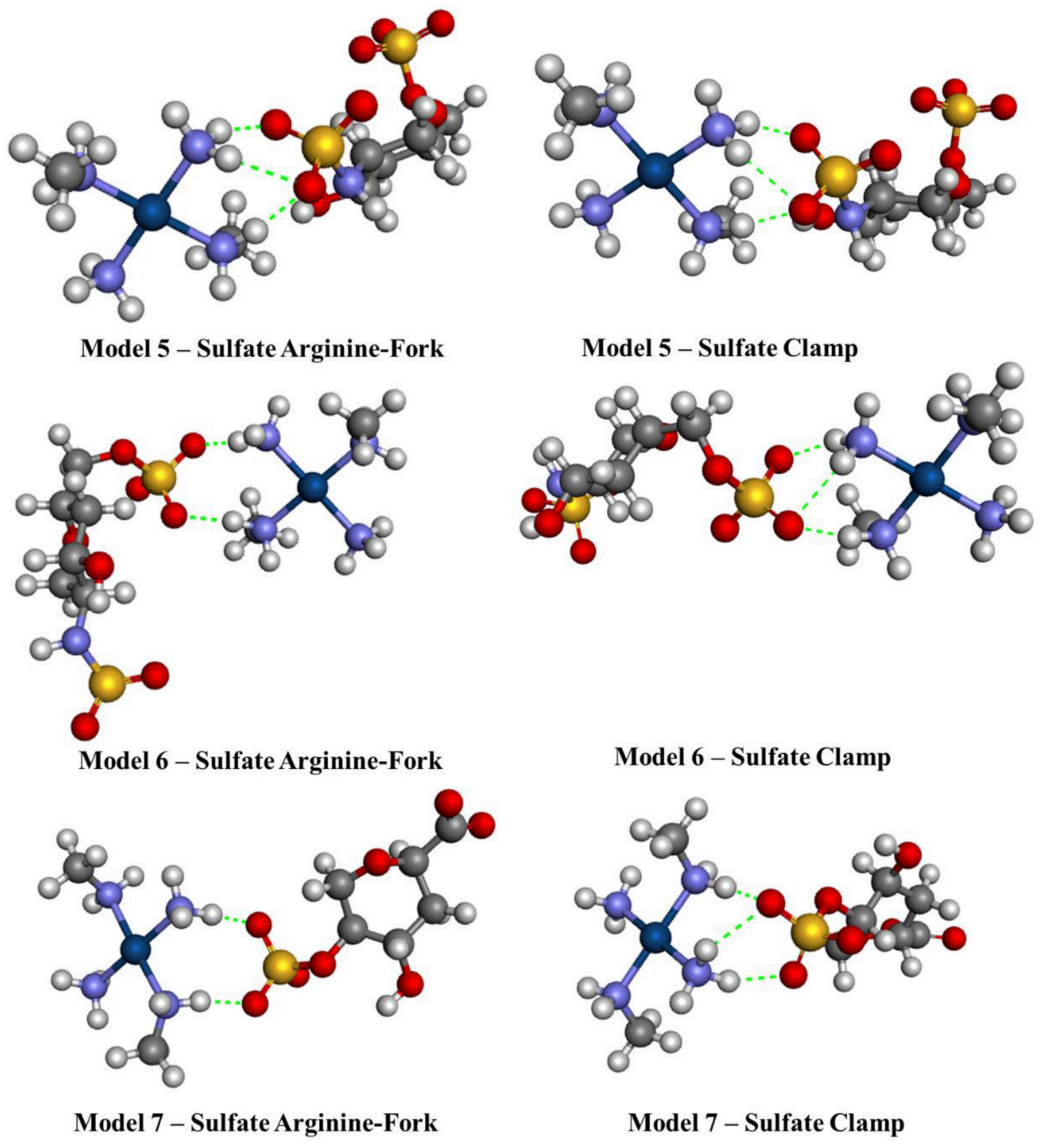
Optimized structures of each different sulfate models 5–7 using the BHandH functional.

Figures 7, 8 show the structures optimized for the 1–4 and 5–7 models, respectively. It is possible to note the formation of non-covalent interactions in all the structures obtained. In the models 2, 3, 5, and 7 we can observe the formation of both structures, phosphate clamps and phosphate arginine-forks. Table 3 shows the interaction distances involved in the described structures. The mean values obtained for the distances x and y are below the mean values reported by Komeda et al. (2006). The values of ν_1_ and ν_1_ are above the experimental average values. The mean values of the angles ω_1_ and ω_2_ are smaller than the experimental data, this is due to the contribution of the results obtained for the arginine-forks that presents a structure in which two oxygen atoms interact with two amino groups in *cis* position, presenting values of ∠N–O–S angles smaller than those presented by the phosphate clamps.

**Table 3:** Distances (in Ångström) and bond angles (in degree) involved in the formation of clamps and arginine-forks.

**Models**		**Distances/Å**		**Angles/^°^**			
		**x**	**y**	**ν_1_**	**ν_2_**	**ω_1_**	**ω_2_**
Phosphate Arginine-fork	1	2.70	2.67	111.9	112.7	130.6	113.9
	2	2.72	2.70	108.3	108.0	129.9	112.7
	3	2.68	2.68	114.2	112.6	114.2	130.7
	4	2.71	2.69	105.4	93.9	107.9	127.9
Phosphate Clamp	1	2.71	2.70	100.8	100.4	135.3	134.3
	2	2.92	2.72	105.4	99.2	128.9	90.9
	3	2.85	2.71	105.9	102.3	125.7	92.8
	4	2.76	2.71	102.3	100.7	126.3	124.5
Sulfate Arginine-Fork	5	2.81	2.80	106.1	110.6	109.6	98.2
	6	2.75	2.75	124.7	116.0	114.4	122.9
	7	2.76	2.76	109.5	104.1	128.5	122.7
Sulfate Clamp	5	2.89	2.80	105.9	102.9	109.5	94.6
	6	2.89	2.79	104.5	101.1	113.0	94.5
	7	2.85	2.79	105.5	103.6	122.2	97.1
Mean		2.79	2.73	107.9	104.9	121.1	111.3

**Figure 9:**
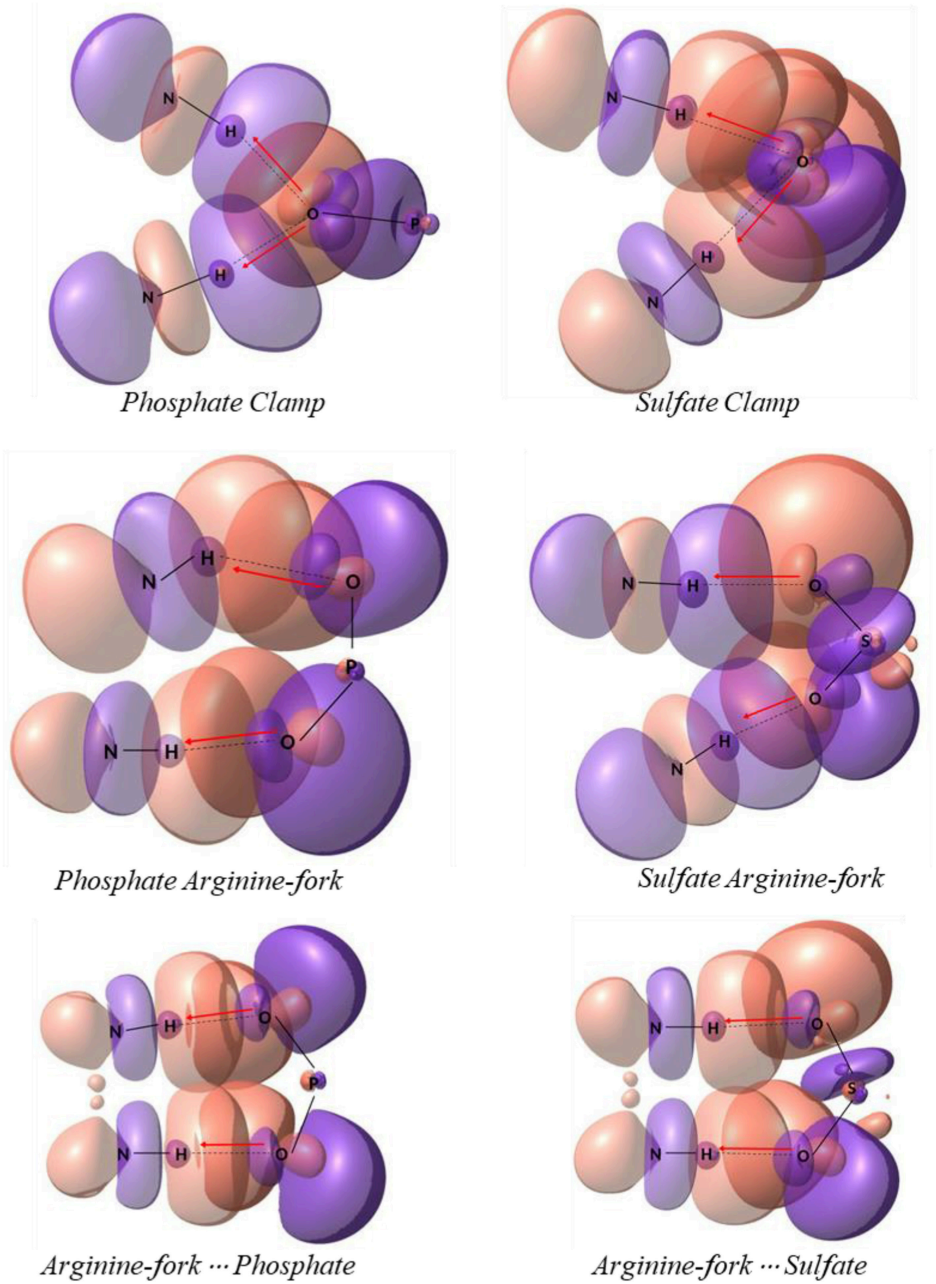
Natural bonding orbitals of models displayed on Figure 5. The electron donation is depicted by red arrows from oxygen atoms to N–H bond.

In order to obtain some detailed information about the bonds and specially the non-covalent interaction a population analysis was performed using the NBO method using the same level of theory mentioned in methodology section. NBO shows all bonding orbitals and provides a clear view of charge donation throughout the platinum models studied, which includes phosphate clamps and arginine-forks. In Figure 9 one can find the orbitals involved with the studied hydrogen interactions that generates clamps and arginine-forks for the models shown in the Figure 5; it is not hard to notice the orbitals superposition that stabilizes the system. The orbitals involved in models 1 to 7 are presented as Supplementary Images (Figures S1, S2) (Zhurko, 2019).

The red arrows drawn on each picture illustrates the donation of a lone pair orbital to an antibonding one (n → BD^*^). To sum up, all hydrogen interactions helped to stabilize the models suggested. When comparing both sulfates from HS and phosphates from DNA there are a slightly difference in energy that indicates the phosphate interactions are more stable.

The energy values for those superpositions have been reported on Table 4. The letters x and y (as displayed in Figure 6) are the notation used to represent the interactions of hydrogen atoms from the NH_3_ and NH_2_ groups, respectively. Values correspond to the stabilizing energies, that is, in fact, how the formation of this interaction stabilizes the system itself. Because the clamp and fork structures are generated by the presence of the two interactions simultaneously one can be seen on the last column the summation of each bond contribution. The same table including the orbital occupation can be found in the Supporting Material (Table S1).

**Table 4:** Second-order perturbative energy values of donor-acceptor (bond-antibond) interactions from NBO calculations for the H-bonds involved in the formation of clamps and Arginine-forks using the BHandH functional.

		$E_{lig.}{}_{y}^{a}$	$E_{lig.}{}_{y}^{a}$	$E_{TOTAL}^{b}$
Phosphate	Clamps	33.94	36.09	70.03
	Arginine-forks	28.06	40.3	68.36
Sulfate	Clamps	26.42	27.55	53.97
	Arginine-forks	28.08	29.43	57.51
Arginine-Forks	Phosphate	43.82	45.18	89.00
	Sulfate	32.53	32.24	64.77

*All values are displayed in kcal mol^−1^*.

*^a^The index x and y corresponds to each bond between oxygen and hydrogen atoms. Please, refer to Figure 6 for clarity*.

*^b^E_TOTAL_ corresponds to the summation of each column values*.

When comparing the stabilization energies for the clamps, it is visible that the phosphate one shows better stability than the sulfate, 70.03 and 53.97 kcal mol^−1^, respectively. The same pattern can be seen in the forks and specially in the arginine structure (in which the difference is up to 25 kcal mol^−1^). In the other hand, when comparing the phosphate ones with the platinum center it shows a slightly higher energy when forming clamps, contrarily to the sulfates that appears to be interacting more efficiently in a fork-shaped structure. In all studied models the oxygen's lone pair from the sulfate/phosphate to the antibonding orbital of the N–H bond is clearly the primary donation. Such electron transfer is responsible for the interactions that stabilize all models and form the studied clamps and arginine-forks.

**Figure 10:**
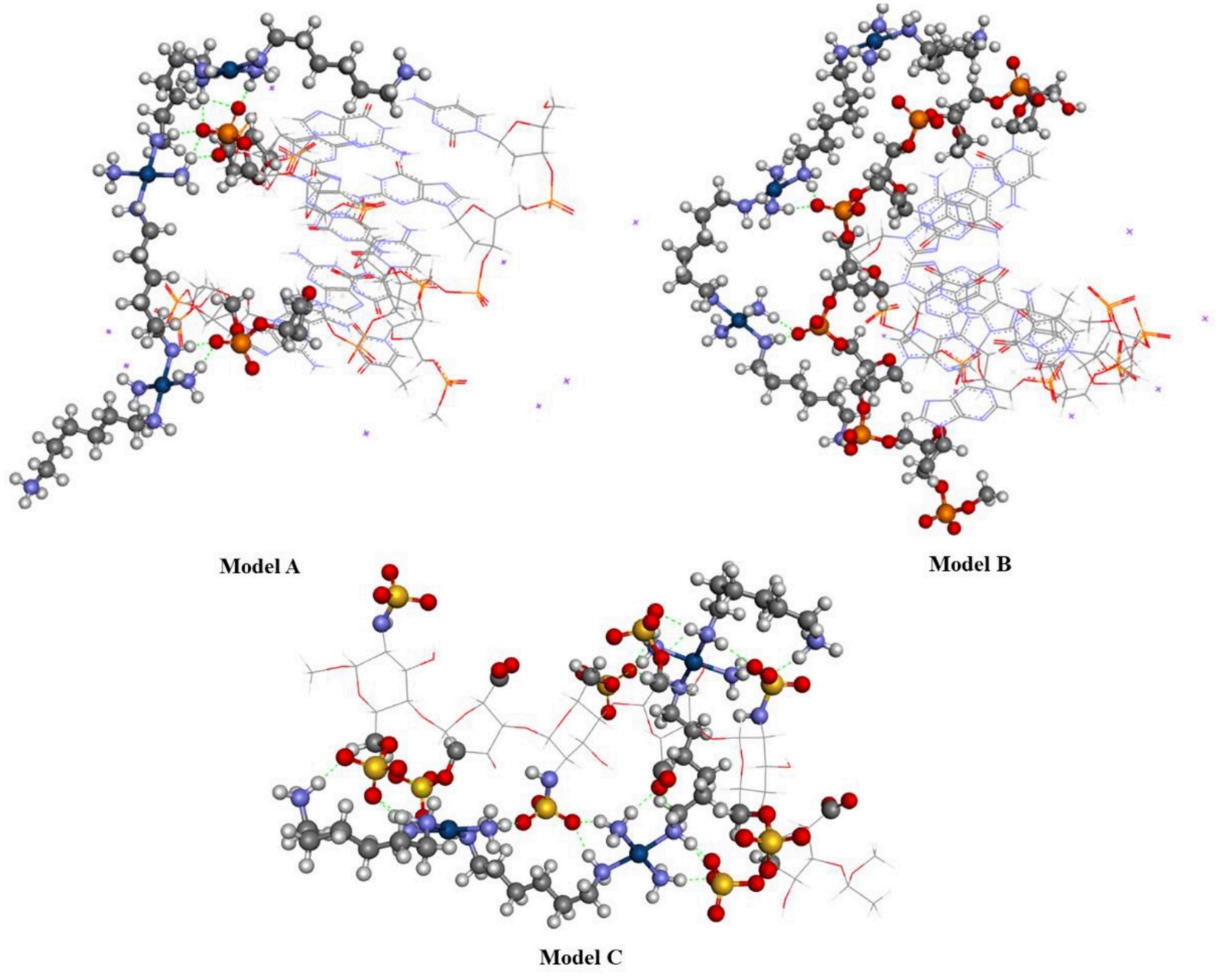
Optimized structure of models (A–C) using the functional BHandH.

Figure 10 shows the optimized structure obtained for Models A, B, and C using the BHandH functional. It is possible to observe the formation of the phosphate clamps and arginine-fork between the TriplatinNC complex and oxygen atoms from phosphate groups of two guanine nitrogen bases. The interactions of the type hydrogen bonds present in model A feature distances between the hydrogen and oxygen atoms ranges from 1.61 and 2.44 Å. For Model B and C, the distances are between 1.68 and 1.76 Å and 1.63 and 2.29 Å, respectively. In Model A, a total of three phosphate clamps and two arginine-forks were observed, and the NH_3_ groups of two Pt centers participate in both clamps and arginine-forks. Two structures of the phosphate clamps type were observed in the Model B. There was no formation of any arginine-fork structure in this model. The average distance between the oxygen atoms of the phosphate groups and the hydrogen atoms of the amino groups present in the A and B models is 1.83 Å. For Model C, the average distance between the hydrogen atoms of the amino groups and the oxygen atoms of the sulfate groups is 1.93 Å.

Model C showed a significantly higher number of non-covalent interactions compared to models A and B. This difference can be justified by the presence of a higher number of sulfate groups in heparin compared to the number of phosphate groups present in the DNA in the models analyzed. According to Katner et al. (2018) heparin has a mean of 2.7 sulfate groups per disaccharide compared to the mean value of two phosphate groups per base pair present in DNA.

Table 5 shows the main values for distances and connection angles involved in the formation of the phosphate and sulfate clamps and arginine-forks. These calculated values are close to the experimental ones reported by Komeda et al. (2006) as can be seen in Table 5. The *cis* orientation of the amine groups is essential for the formation of the phosphate clamps and arginine-forks. It is noted that one of the phosphate groups defined in the high layer interacts with two platinum centers, forming two structures of phosphate clamps and two phosphate arginine-forks.

**Table 5:** Distances (in Ångström) and bond angles (in degree) involved in the formation of non-covalent interactions for models A–C.

		**Distances/Å**		**Angles/^°^**			
	**x**	**y**	**ν_1_**	**ν_2_**	**ω_1_**	**ω_2_**
Model A	PC1^a^	2.64	2.99	91.4	102.5	99.3	138.7
	PAF1^b^	2.64	2.83	113.2	102.5	106.4	138.7
	PC2	2.72	2.75	103.3	104.3	126.5	111.4
	PAF2	3.03	2.75	103.3	105.9	126.5	97.9
	PC3	2.73	2.73	101.5	101.8	121.5	127.7
Model B	PC1	2.72	2.76	102.6	104.1	167.4	118.2
	PC2	2.69	2.76	100.3	102.3	111.6	155.8
Model C	SC1^c^	2.89	2.86	102.0	101.0	96.5	138.4
	SAF1^d^	2.89	2.87	128.7	101.0	96.8	138.4
	SC2	2.80	2.95	97.6	102.7	115.3	109.1
	SC3	2.78	2.75	102.5	101.7	140.2	148.6
	SC4	2.76	2.82	92.3	94.7	147.6	141.9
	SC5	2.86	3.02	97.0	101.4	97.7	125.6
	SAF2	2.86	2.93	108.2	101.4	101.9	125.3
	SC6	2.80	2.92	96.5	100.2	155.9	135.4
	SC7	2.89	2.94	103.9	105.9	110.4	101.3
	SAF3	2.89	3.04	85.7	105.9	101.8	101.3
	SC8	2.86	2.78	105.8	103.5	117.9	106.3
	SAF4	2.86	2.78	105.8	118.8	117.9	101.7
Exp.	PC_mean_	2.90	2.95	103	100	127	140

a *PC, phosphate clamp*.

b *PAF, phosphate arginine-fork*.

c *SC, sulfate clamp*.

d *SAF, sulfate arginine-fork*.

## Conclusion

The study of hydrogen bond interactions by phosphate clamps and arginine-forks through mimetic models has been the main point described in this research paper. The mimetic models approach confirms that TriplatinNC complex has the capability to interact non-covalently with the phosphate groups present in the DNA and sulfate groups present in the HexaHS through the presence of the interactions such as phosphate clamps and arginine-forks that make possible the formation of a bidentate structure of the amino ligands and oxygen atoms. The interaction energies values suggest that the BHandH functional along with 6–31+G(d,p)/SDD basis sets provides the best values, confirming that such methodology is adequate for the analysis of the types of interaction studied in this work. NBO analysis proved efficient in describing the donation process for the models proposed here.

The structures obtained with a DNA fraction containing 6 nitrogenous base pairs and the TriplatinNC complex confirm the formation of the phosphate clamps through the formation of hydrogen interactions between the hydrogen atoms from amine groups in the *cis* position of the platinum center, with the same oxygen atom of a phosphate group present in the DNA strand. Arginine-fork structures have also been observed and have similar structures to clamps. These interactions contribute to complex-DNA interaction.

It is known that molecules such as cisplatin can find different biomolecules in which it is possible to form bonds, thus presenting a competition of several molecules to each other, affecting bioavailability, with only a small fraction of the compound effectively binding to DNA. The results shown in this work suggest that non-covalent interactions can occur with other biomolecules other than DNA, so that the similarity of sulfate groups present in heparin with the phosphate groups of DNA allows the interaction of the TriplatinNC complex with the heparin. The size of the heparin model used was shown to be efficient interacting with the three platinum centers present in TriplatinNC. The formation of eight sulfate clamps structures and four arginine-fork structures were noted in this work.

## Author Contributions

NMPR conducted the simulations of the structures of the phosphate groups. FHCF conducted the simulations of the structures of the sulfate groups. FHCF and LASC conducted the NBO analyzes. NMPR and FHCF obtained the structures using the ONIOM methodology. NMPR, FHCF, NPF, and LASC participated in the discussion of the results. NMPR, FHCF, and LASC wrote the manuscript together.

### Conflict of Interest Statement

The authors declare that the research was conducted in the absence of any commercial or financial relationships that could be construed as a potential conflict of interest.

## Supplementary Material

Data Sheet S1 Figures S1 and S2 show NBO of models 1–7. Table S1 shows details about NBO calculations. Spatial coordinates (xyz) of the optimized structures are available.
