# Supplementary material for: TriplatinNC and Biomolecules: Building Models Based on Non-covalent Interactions
Source: Front Chem. 2019 May 21;7:307. doi: 10.3389/fchem.2019.00307 (PMC6558404; doi:10.3389/fchem.2019.00307)
Supplement: Data Sheet S1 — Figures S1 and S2 show NBO of models 1–7. Table S1 shows details about NBO calculations. Spatial coordinates (xyz) of the optimized structures are available. [file Data_Sheet_1.docx]

***Supporting Information***

**TriplatinNC and Biomolecules: building models based on non-covalent interactions**

Nathália M. P. Rosa^*1^, Frederico Henrique do C. Ferreira^1^, Nicholas P. Farrell^2^ and Luiz Antônio S. Costa^1*^

^1^NEQC – Núcleo de Estudos em Química Computacional, Departamento de Química, ICE, Universidade Federal de Juiz de Fora, Juiz de Fora, MG, 36033-900, Brasil.

^2^Department of Chemistry, Virginia Commonwealth University, Richmond, Virginia, 23284, USA.

**Table S1.** Energy values of donor-acceptor (bond-antibond) interactions from NBO calculations for the H-bonds involved in the formation of clamps and arginine-forks using the BHandH functional. The last column displays the occupancy of the oxygen’s lone pair (LP) All energy values are displayed in kcal mol^-1^.

**
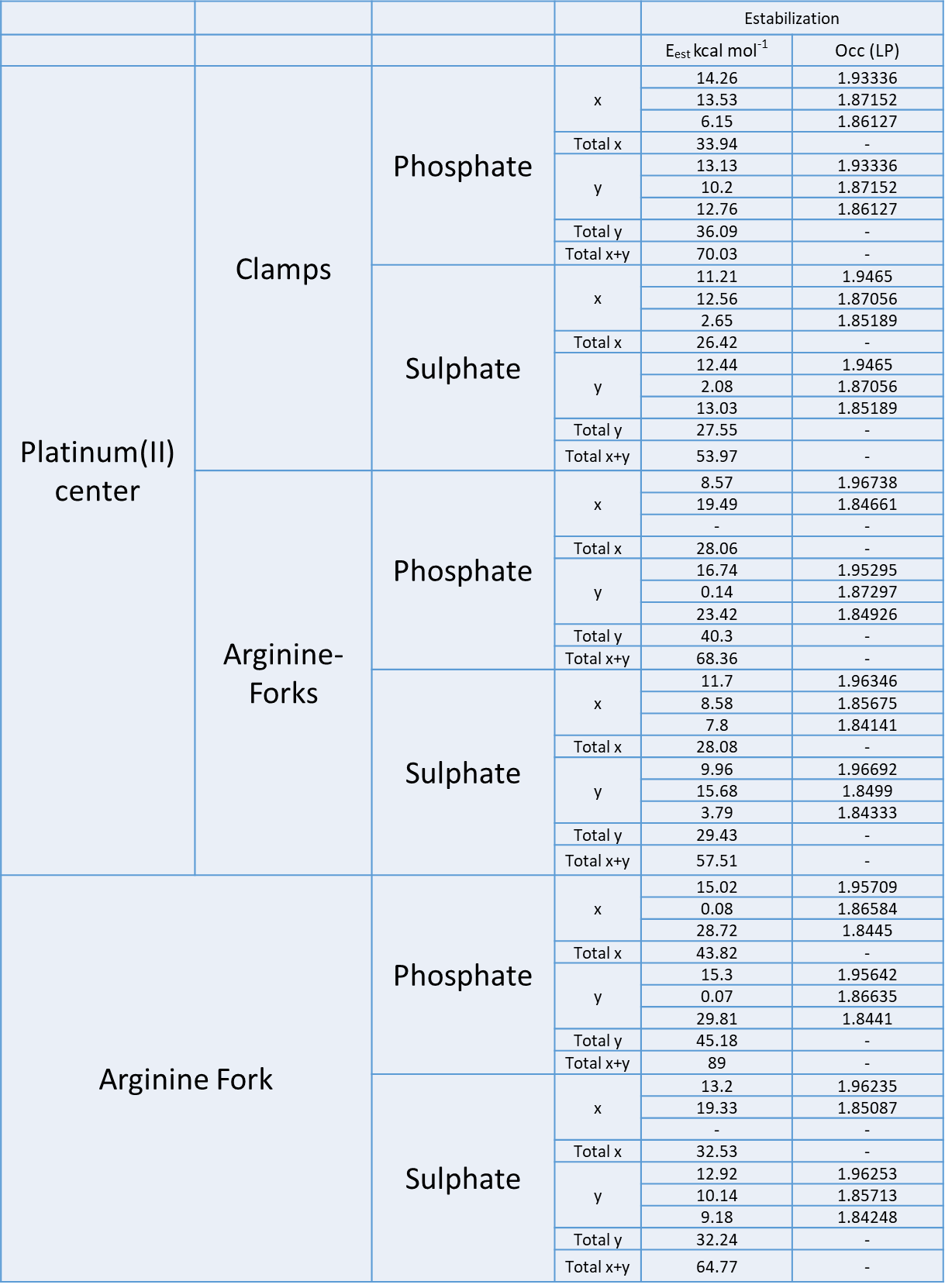
**

**
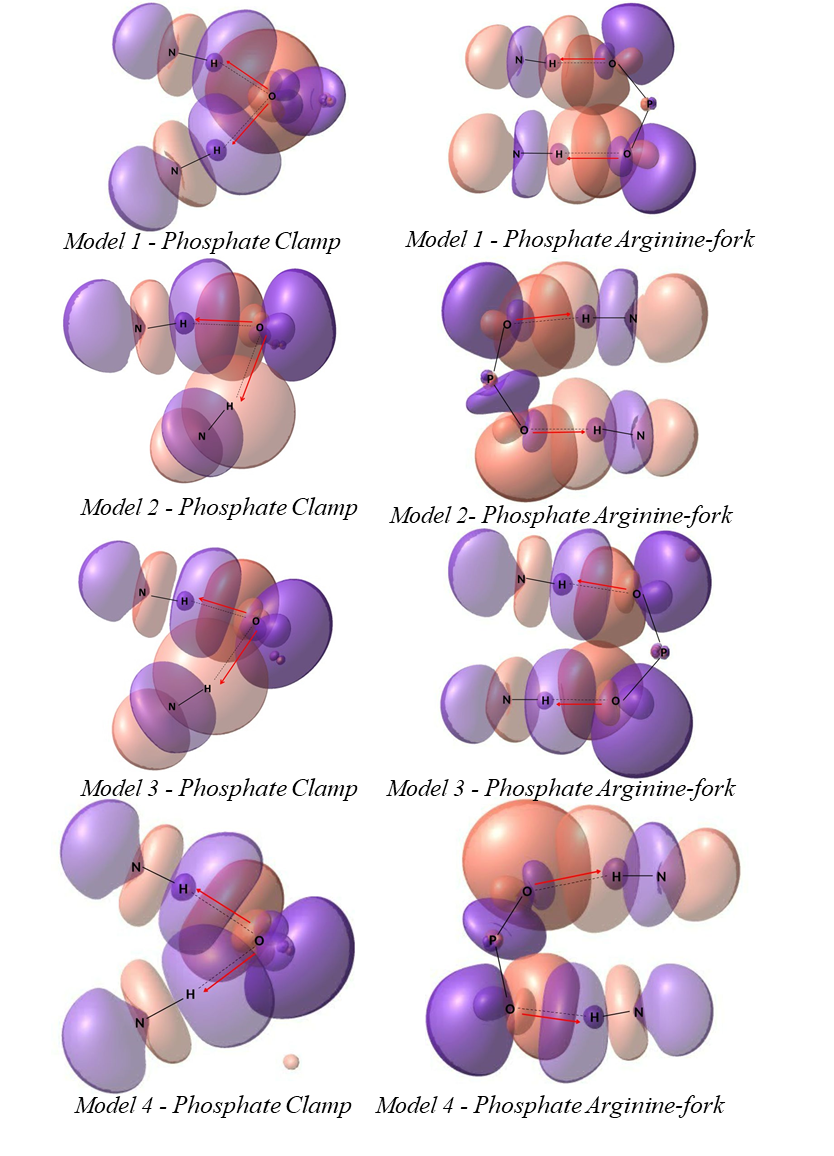
**

**Figure S1.** Natural Bonding Orbitals of models displayed on Figure 7. The electron donation is depicted by red arrows from oxygen atoms to N−H bond.

**
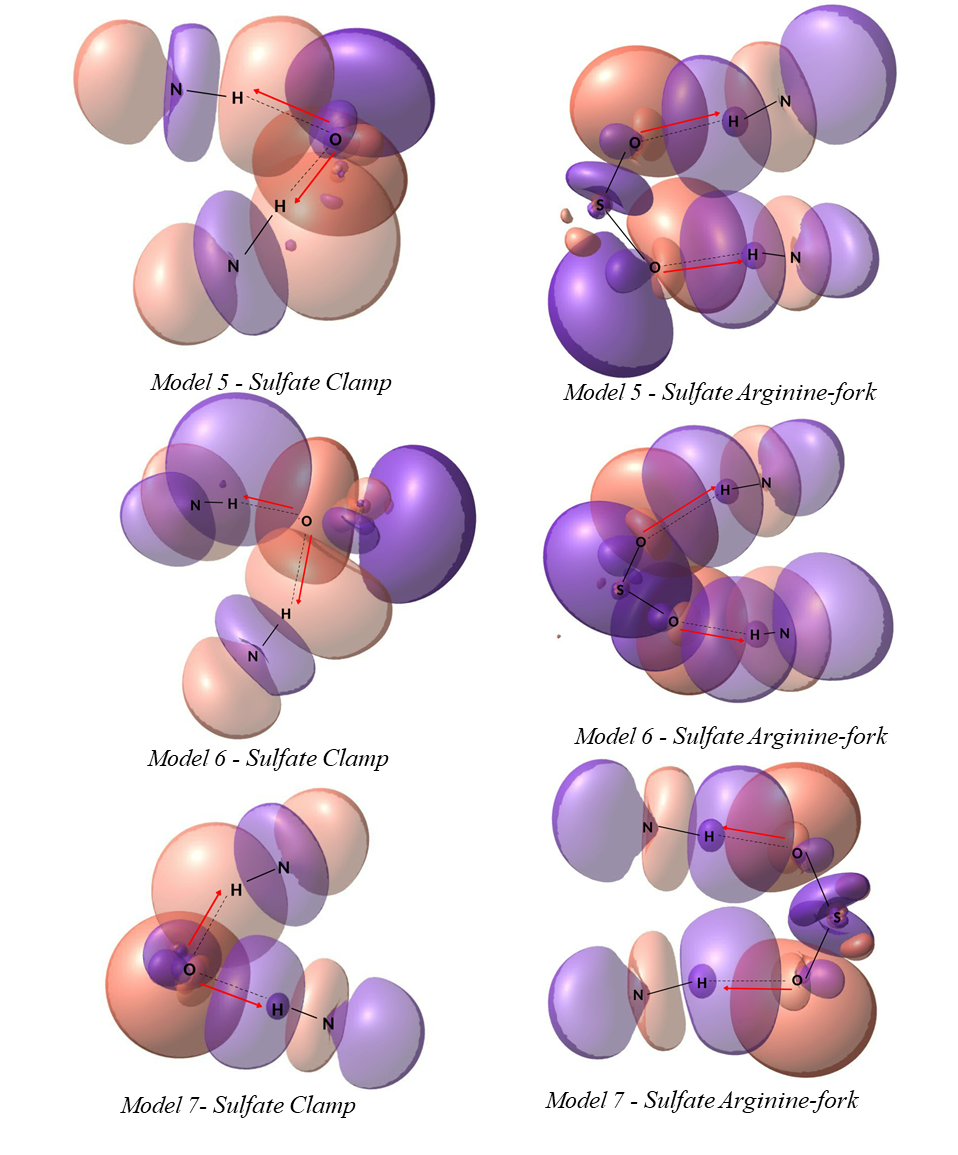
**

**Figure S2.** Natural Bonding Orbitals of models displayed on Figure 8. The electron donation is depicted by red arrows from oxygen atoms to N−H bond.

**This file contains all coordinates of the optimized structures related to this work.**

1. Coordinates of the optimized molecules shown in figure 5 using the BHandH functional.

*Phosphate Clamp*

1 1

C 5.36330500 1.56475100 0.07899400

H 5.46452600 1.63053000 1.16316400

O 4.02148200 1.35613600 -0.28359500

P 3.27422700 0.07727800 0.32417800

O 1.90492800 0.11469200 -0.29576900

O 3.43299800 -0.00356600 1.79285600

O 4.09285000 -1.14258200 -0.31116700

C 4.11817700 -1.27016800 -1.71064400

H 4.61723100 -0.41194800 -2.16686100

H 3.10518600 -1.34800900 -2.10851100

C -0.15225400 -1.73623700 1.67031300

N -0.27845000 -1.37837000 0.26097700

N -3.10505900 -1.58079900 -0.31748700

N -0.40912200 1.47164300 0.02440900

Pt -1.76241200 -0.04001400 -0.14855800

N -3.24780000 1.32602100 -0.52719000

C -4.17183900 1.53683600 0.58778100

H -1.07261600 -2.18977200 2.03261800

H 0.04463600 -0.83688300 2.25039500

H -0.38798700 -2.22325700 -0.29016600

H -3.92371700 -1.35545200 -0.87397700

H -2.70161500 -2.40287000 -0.75614900

H -0.50812800 2.17452300 -0.70043300

H 0.56021900 1.10532600 -0.04667300

H -3.77075100 1.04774200 -1.35231700

H -4.65311400 0.59679500 0.84721900

H -3.61492700 1.89050800 1.45152200

H -2.84078600 2.22049000 -0.78311700

H -0.50144400 1.94979700 0.91520300

H -3.44523900 -1.87446200 0.59390800

H 0.61951500 -0.95692000 -0.03902400

H 4.67296200 -2.17501000 -1.94662400

H 5.68295000 2.50003200 -0.37404700

H 5.99308000 0.75218800 -0.29158500

H 0.67136400 -2.43634200 1.81441600

H -4.93604200 2.26688000 0.32369100

*Phosphate Arginine-fork*

1 1

C 5.00362300 0.64108000 -1.03217300

H 5.82380900 0.36456100 -1.68987800

O 4.13527700 -0.46285900 -0.95332700

P 2.77604700 -0.29229800 -0.13282700

O 2.07503800 -1.60729100 -0.17982700

O 2.04404300 0.92809200 -0.59442800

O 3.29221600 0.02945500 1.34493700

C 4.04273900 -0.95755600 2.00924200

H 4.98615100 -1.13850600 1.48875800

H 3.48184400 -1.89125000 2.07034700

C -0.79460000 2.79303100 -0.75187000

N -0.40357800 1.75208300 0.19285900

N -2.79021000 0.78468900 1.54633900

N -0.24879400 -0.66976100 -1.30110300

Pt -1.52747900 0.05209200 0.10576800

N -2.63555600 -1.67349000 -0.02533300

C -3.65528100 -1.66855000 -1.07441900

H -1.81760300 3.11389800 -0.56552900

H -0.73929200 2.40017500 -1.76525900

H -0.40081000 2.13611900 1.13250200

H -3.73719900 0.42443200 1.48007600

H -2.45380100 0.55049500 2.47602700

H -0.70121800 -1.19839000 -2.03880500

H 0.49230000 -1.25141000 -0.86715800

H -3.07708100 -1.87423400 0.86702400

H -4.35659900 -0.85772700 -0.89399400

H -3.18330500 -1.50363300 -2.04008900

H -2.00877300 -2.45917200 -0.17309900

H 0.27596300 0.08106200 -1.74430700

H -2.88017600 1.79539300 1.51336900

H 0.58204000 1.48059200 -0.00181500

H 4.25063000 -0.59060600 3.01115600

H 5.40056600 0.88931000 -0.04515700

H 4.48467300 1.50990600 -1.43965900

H -0.13025000 3.65377800 -0.67026100

H -4.19373700 -2.61530500 -1.09463100

*Sulfate Clamp*

1 1

O 3.84299500 -1.57342100 -0.41393400

S 3.55076200 -0.16663300 -0.23309100

O 2.31511900 0.23179000 -0.93437400

O 3.21765700 0.03514700 1.31268500

O 4.64580500 0.74652100 -0.45007900

C -3.90345900 -1.21574800 -0.69149600

H -3.64676600 -1.00618400 -1.72649600

H -4.40305100 -0.34561200 -0.27239000

N -2.68068500 -1.49569700 0.06120700

H -2.26712000 -2.34631400 -0.30779600

H -2.91964900 -1.73263100 1.01985400

Pt -1.29550000 0.01880200 0.04625800

N -2.52670800 1.17488500 1.20859800

N 0.06243000 1.54830900 -0.01269500

N -0.07487300 -1.13129500 -1.11234800

H -3.20419800 1.67993700 0.64365900

H -2.02846500 1.87398600 1.75057800

H 0.98039300 1.17743700 -0.29213000

H 0.20798600 1.92598700 0.91907400

H 0.00820100 -2.08355300 -0.76966400

H -3.05421700 0.63699100 1.88953100

H 0.88334900 -0.75464400 -1.15404000

H -0.42208400 -1.18864400 -2.06513400

C -0.29262200 2.63629900 -0.92251400

H -1.24309200 3.07730600 -0.62964000

H -0.38940700 2.24157600 -1.93103300

H 0.47537800 3.40950100 -0.91116000

H -4.58267900 -2.06622800 -0.64955600

C 2.23626200 -0.82124900 1.85583200

H 2.52054400 -1.86325300 1.71120900

H 2.18756400 -0.59166400 2.91618600

H 1.25925600 -0.63392200 1.40160000

*Sulfate Arginie-Fork*

1 1

O -4.28816700 -1.40160800 -0.78092900

S -3.20960900 -0.63013400 -0.20442600

O -2.43619000 0.12264600 -1.19169200

O -3.87650100 0.46199700 0.74157800

O -2.36048800 -1.35585500 0.73184700

C 4.13427700 -0.87440900 0.74712200

H 3.69389100 -1.13886300 1.70484100

H 4.43957300 0.16868600 0.78560800

N 3.13937700 -1.06047500 -0.31055800

H 2.89387900 -2.04503700 -0.34943400

H 3.56793900 -0.87845000 -1.21376200

Pt 1.44801600 0.08649200 -0.11195400

N 2.47723800 1.75150600 -0.71287000

N -0.22500700 1.23010100 0.09655300

N 0.40631000 -1.56708300 0.46874800

H 2.45567500 2.48135900 -0.00599000

H 2.08450700 2.14452100 -1.56363400

H -0.97878400 0.83540200 -0.48639800

H -0.06521500 2.15905200 -0.27985900

H 0.51258700 -2.32792200 -0.19568400

H 3.45883200 1.58803600 -0.91365500

H -0.61170000 -1.42533500 0.55847400

H 0.73163400 -1.91273000 1.36693400

C -0.72430600 1.37026600 1.46355300

H 0.03411800 1.83540400 2.08844900

H -0.96798400 0.38791300 1.86170700

H -1.63251900 1.97383100 1.47353300

H 5.01015100 -1.49599200 0.56536500

C -4.76644200 1.35792700 0.10816800

H -5.59471600 0.81212100 -0.34382600

H -5.13838100 2.01593500 0.88775600

H -4.24084000 1.93937300 -0.64976400

1. Coordinates of the optimized molecules shown in figure 7, models 1, 2, 3 and 4, using the BHandH functional.

*Model 1: Phosphate Arginine-Fork*

1 1

P 0.24344800 -1.02571700 1.75716400

O -1.01101700 -1.81643300 1.90278200

O 0.15156500 0.33929700 1.14715900

O 1.33793700 -1.86164500 0.93244300

C 0.98035400 -2.33190200 -0.34520300

H 0.32886600 -1.61587400 -0.85538600

H 0.44974300 -3.28183400 -0.24542400

C 2.22396700 -2.52533900 -1.15283400

H 1.98103700 -3.12670900 -2.03261700

O 2.71938600 -1.27463900 -1.58198000

C 4.00344900 -1.04152600 -1.09358800

H 4.75502200 -1.19988300 -1.86870700

N 4.10844300 0.35346000 -0.75214600

C 3.11506500 0.95703500 -0.03168700

H 2.26811900 0.32533300 0.21544000

C 3.14678500 2.24794700 0.32534000

C 2.06839300 2.92105100 1.08346700

H 2.44634700 3.31168100 2.03061700

H 1.67614200 3.77413100 0.52409800

H 1.25075000 2.22535600 1.27339800

C 4.29319400 3.02561300 -0.07003300

O 4.45756900 4.20226300 0.18773400

N 5.23628600 2.34394700 -0.79633400

H 6.05220900 2.86297100 -1.08859300

C 5.21207700 1.03623500 -1.16402900

O 6.11493700 0.53408100 -1.80143100

C 3.35324500 -3.18469700 -0.37324600

H 2.96360200 -3.74327600 0.47639000

C 4.17568100 -1.99682900 0.06249900

H 3.74053900 -1.56386900 0.96374000

H 5.21723100 -2.25495300 0.24661400

O 4.07093400 -4.09916200 -1.14685600

O 0.96439200 -0.95396700 3.16589200

H 4.46835400 -3.67069600 -1.90762900

C -2.17178200 2.74623800 0.62675400

N -1.86164100 1.53973500 -0.13316700

N -3.26779500 -0.38783400 1.46061300

N -3.55342900 0.92452500 -2.40098900

Pt -3.42164900 0.26826100 -0.46136800

N -4.99632900 -1.02562600 -0.74612700

C -6.26470600 -0.59013500 -0.15777300

H -2.55733800 2.47484700 1.60750100

H -2.92512800 3.34019600 0.11437800

H -1.10547000 1.02344000 0.36593700

H -4.04007400 -0.98104800 1.74618400

H -2.39623200 -0.93944100 1.63139700

H -3.00577800 0.33398600 -3.02063100

H -4.50169600 0.93147100 -2.76382400

H -4.74634900 -1.93588200 -0.36872100

H -6.14751900 -0.46074000 0.91587300

H -6.55445300 0.36518800 -0.58684500

H -5.13988000 -1.20402200 -1.73535600

H -3.21125000 1.87203500 -2.52623800

H -3.26740000 0.38499900 2.11912900

H -1.44182000 1.81449300 -1.01555600

H -1.27165600 3.34732500 0.76528200

H -7.04730900 -1.32476200 -0.34313600

C 2.12987100 -0.17688500 3.30227700

H 2.96233800 -0.63793100 2.76697200

H 1.96908500 0.83348300 2.92289400

H 2.36890800 -0.13529100 4.36151900

*Model 1: Phosphate Clamp*

1 1

P 0.49505600 -0.03605400 1.31319800

O -0.83143800 -0.69271300 1.04001900

O 0.67970300 1.40462000 1.03466000

O 1.60900500 -0.90952200 0.54538200

C 1.30267300 -1.54325200 -0.66930000

H 0.94563300 -0.81698400 -1.40549500

H 0.52517500 -2.29498500 -0.51110600

C 2.54885100 -2.19011100 -1.18550900

H 2.28712200 -2.81813600 -2.04098100

O 3.46133200 -1.19960100 -1.61017300

C 4.63888700 -1.22686800 -0.86115500

H 5.45871400 -1.64530800 -1.44630800

N 5.03019300 0.12980600 -0.58148000

C 4.12228100 0.98597300 -0.02307500

H 3.15579500 0.54241300 0.19341600

C 4.38142600 2.27522100 0.22877800

C 3.39762400 3.20505600 0.82796800

H 3.76817500 3.60471800 1.77465300

H 3.22834300 4.06107900 0.17103200

H 2.44813200 2.69366800 0.99915000

C 5.68917200 2.77215500 -0.11493600

O 6.06825100 3.91412200 0.05710300

N 6.53351900 1.84879600 -0.67882100

H 7.45662400 2.17081300 -0.93373300

C 6.27547100 0.54120100 -0.94396500

O 7.10328000 -0.18415900 -1.45568500

C 3.28442900 -3.02013300 -0.14360800

H 2.60772400 -3.34646300 0.64543600

C 4.32468000 -2.05244800 0.36530100

H 3.88448800 -1.41711300 1.13484000

H 5.20003600 -2.55748100 0.77062600

O 3.83342000 -4.18534700 -0.68285800

O 0.92905000 -0.30892000 2.81815700

H 4.46014300 -3.97995700 -1.37944900

C -3.93015100 -1.34874400 2.14250900

N -3.41782000 -0.10310900 1.57966400

N -2.54519900 -0.70610600 -1.07157600

N -5.65404500 1.40923200 0.53119800

Pt -4.10628600 0.34301200 -0.28800600

N -4.79203300 0.78317100 -2.17321300

C -5.85317200 -0.09435600 -2.66832500

H -3.65681500 -2.17470100 1.48909300

H -5.01492300 -1.31526400 2.22031100

H -2.38722900 -0.18225500 1.52123700

H -2.14944900 -0.27371800 -1.89968900

H -1.77910400 -0.77617500 -0.37631900

H -5.34264600 2.33151400 0.82234200

H -6.43431600 1.55674500 -0.10133100

H -4.02297000 0.76673900 -2.83621200

H -5.48611000 -1.11664100 -2.70477400

H -6.70465100 -0.05906800 -1.99263900

H -5.11436600 1.74651800 -2.19521200

H -6.04732700 0.95743000 1.35123200

H -2.82193800 -1.64678900 -1.33508900

H -3.60441400 0.65926600 2.22321400

H -3.50620900 -1.52454600 3.13157800

H -6.17358100 0.21193300 -3.66348700

C 0.85857700 -1.62497100 3.30993800

H -0.15213400 -2.02138900 3.20222000

H 1.55987800 -2.26875700 2.77470500

H 1.12905600 -1.59392600 4.36230000

*Model 2: Phosphate Arginine-Fork*

1 1

P -0.72333900 2.84373600 0.68740200

O 0.16227300 3.11885900 -0.48334000

O -0.32331700 1.80399400 1.67936200

O -2.18634600 2.46744400 0.15825600

C -2.57194300 2.53510900 -1.18955700

H -1.69507500 2.47046900 -1.83547500

H -3.08165000 3.48417300 -1.37897400

C -3.51429800 1.40573300 -1.46258000

H -3.95417900 1.54528300 -2.45436300

O -2.80483900 0.18444000 -1.43355300

C -3.36684600 -0.69617700 -0.50969400

H -4.02619300 -1.41642100 -1.00573800

N -2.32182300 -1.48595200 0.05961800

C -1.19940600 -1.03988300 0.67123500

H -1.01482300 0.01632000 0.84931700

N -0.39823600 -2.00534200 1.02081500

C -1.03701700 -3.14846200 0.62449200

C -0.71634100 -4.50426600 0.71563900

N 0.40448300 -4.94259800 1.28656200

H 0.53197900 -5.93311500 1.39990800

H 0.97206100 -4.32099100 1.83449100

N -1.56481700 -5.38112700 0.18997100

C -2.67546700 -4.94529300 -0.38612000

H -3.32216700 -5.71222200 -0.79711200

N -3.08827300 -3.70251200 -0.51404700

C -2.23063800 -2.84185400 0.00923200

C -4.62396600 1.28168600 -0.42420700

H -4.75499300 2.21598500 0.11946000

C -4.10968800 0.17896400 0.46811700

H -3.40199600 0.59492500 1.18704200

H -4.90678600 -0.34342200 0.99447500

O -5.86575000 0.99672600 -0.99569100

H -5.83453100 0.18691900 -1.50865600

O -0.99039600 4.17678600 1.51738800

C -1.48239600 5.29516300 0.82156700

H -2.48177100 5.09237300 0.42930500

H -1.53916700 6.12036000 1.52677500

H -0.81606400 5.56256100 -0.00003900

C 2.69155000 0.19817100 2.70975900

N 1.73201000 0.05200600 1.62271700

N 2.66764100 2.08963900 -0.17235400

N 2.29513400 -1.97197300 -0.29603100

Pt 2.52900900 0.05804000 -0.25303500

N 3.36821900 0.05334400 -2.12616900

C 4.81971900 -0.13226600 -2.11964300

H 3.23241700 1.13766400 2.60829300

H 3.41432500 -0.61483000 2.68703400

H 1.01512100 0.80057900 1.67960300

H 3.31049100 2.49125200 -0.84672200

H 1.72909100 2.51590000 -0.33516700

H 1.40028300 -2.19209400 0.14893500

H 2.27236900 -2.38366600 -1.22291100

H 3.14260800 0.91615400 -2.61241500

H 5.28335800 0.66483400 -1.54361300

H 5.05861900 -1.08286100 -1.64894000

H 2.94419000 -0.66891700 -2.70051300

H 3.02830900 -2.45042400 0.21854700

H 2.98980700 2.41272900 0.73414600

H 1.20105900 -0.81089100 1.75329500

H 2.18114500 0.19597300 3.67364200

H 5.21657100 -0.12188800 -3.13428600

*Model 2: Phosphate Clamp*

1 1

P 0.39374400 -2.52268200 0.55303200

O -0.17623500 -2.15833000 -0.78333200

O 0.27468500 -1.53927100 1.66783400

O 1.93614400 -2.90067700 0.37355000

C 2.57959200 -3.02259300 -0.86894800

H 1.88868100 -2.76911800 -1.67462500

H 2.91285700 -4.05627400 -0.98897700

C 3.76559000 -2.11070800 -0.89816700

H 4.37903700 -2.35810000 -1.77070900

O 3.31850700 -0.77606400 -0.99538300

C 3.92970400 0.01460900 -0.01785700

H 4.83633700 0.48510800 -0.41397200

N 3.05899900 1.09231200 0.30662800

C 1.85516300 1.01532300 0.93202200

H 1.47627100 0.08149800 1.33899200

N 1.21447700 2.14461200 0.94194700

C 2.04037700 3.01452600 0.28836800

C 1.93736000 4.36999300 -0.03096500

N 0.88490700 5.10807800 0.30954100

H 0.87671400 6.08849900 0.09166000

H 0.15143900 4.72176700 0.87559300

N 2.93445600 4.92953200 -0.70960900

C 3.97975000 4.19085200 -1.05252200

H 4.75587900 4.70679200 -1.60668200

N 4.18976100 2.91635500 -0.80228400

C 3.18831100 2.37519300 -0.12632200

C 4.62311300 -2.19069500 0.36364500

H 4.39295100 -3.08912400 0.93375400

C 4.22512600 -0.94011400 1.10950200

H 3.30895600 -1.13482900 1.67044600

H 5.00251100 -0.58836400 1.78553800

O 5.98947300 -2.26122700 0.08445300

H 6.27743300 -1.51398500 -0.44324000

O -0.23808800 -3.88892400 1.07528200

C -0.15820600 -5.01457300 0.23649300

H 0.88421400 -5.29918400 0.07519700

H -0.67607200 -5.83039600 0.73421300

H -0.63333500 -4.81471600 -0.72534100

C -3.24923600 2.99258600 0.88910800

N -3.47006900 1.62755600 1.36667000

N -5.11972300 -0.12608100 -0.30316900

N -1.11388800 0.40366000 0.25967800

Pt -3.10617500 0.16097500 -0.02360200

N -2.68252900 -1.28569900 -1.39691900

C -2.58994300 -0.81165000 -2.77467700

H -3.92535500 3.20172200 0.06431300

H -2.22824200 3.09098900 0.52706400

H -4.41681100 1.55559200 1.72632800

H -5.33726800 -0.68544900 -1.12192800

H -5.53923300 -0.60683600 0.48743100

H -0.76147800 -0.23760500 0.98928300

H -0.59419800 0.11901100 -0.56787600

H -3.36312800 -2.03700700 -1.34557900

H -3.52942700 -0.35926200 -3.08620700

H -1.80785600 -0.05826500 -2.84481100

H -1.77458500 -1.71974100 -1.13789600

H -0.77988400 1.33768200 0.49718300

H -5.62611800 0.74558400 -0.42469900

H -2.88042600 1.45600700 2.17617000

H -3.41656200 3.71401400 1.68802000

H -2.34863800 -1.63467900 -3.44787500

*Model 3: Phosphate Arginine-Fork*

1 1

P 0.38512400 1.20313600 1.68286400

O 1.75539000 1.77602200 1.79948500

O 0.23997200 -0.15100900 1.06075000

O -0.58654100 2.21219900 0.90245700

C -0.26893300 2.53040000 -0.43079300

H 0.34620100 1.74592000 -0.88067800

H 0.28813500 3.47011500 -0.45319300

C -1.52798800 2.67145900 -1.22623900

H -1.27828300 3.12137300 -2.19100800

O -2.09533900 1.39641000 -1.44575100

C -3.39462100 1.33143300 -0.95422000

H -4.12836800 1.43853500 -1.76033500

N -3.60247600 0.02458700 -0.41349900

C -2.71143200 -0.72919100 0.29586200

H -1.68916200 -0.40682400 0.46747100

N -3.22169900 -1.85343300 0.68763900

C -4.50374100 -1.84119400 0.22185400

C -5.54646800 -2.78862300 0.33361100

O -5.55034200 -3.87049300 0.89385800

N -6.69378200 -2.34673600 -0.31170000

H -7.47738500 -2.98074400 -0.24979900

C -6.83822900 -1.17326000 -0.97680100

N -8.03181800 -0.92341700 -1.51802600

H -8.73663500 -1.63364200 -1.60286000

H -8.11398900 -0.10618200 -2.09655600

N -5.88078400 -0.29664500 -1.07931900

C -4.75419800 -0.67728400 -0.47021000

C -2.59922300 3.50766200 -0.53823800

H -2.16260500 4.14761400 0.22686400

C -3.49608200 2.45069700 0.05554400

H -3.07521200 2.10798000 1.00156100

H -4.51528900 2.80039100 0.21217100

O -3.25993600 4.36463500 -1.42060000

C 2.94506700 -1.58278600 2.22356000

N 2.54688500 -1.50937200 0.81986100

N 4.10255900 0.92223200 0.81050000

N 3.65461100 -2.26156300 -1.71249700

Pt 3.88973000 -0.65785400 -0.45659700

N 5.25127300 0.20213300 -1.72976100

C 6.64606600 0.03335600 -1.32065600

H 3.00806600 -0.57612500 2.63704300

H 3.91232100 -2.07282100 2.32157100

H 1.64410600 -0.98604200 0.78537900

H 4.55003400 1.73239100 0.39286200

H 3.18174600 1.25099500 1.18148800

H 2.72319000 -2.27833200 -2.11919300

H 4.30342900 -2.28074200 -2.49270300

H 5.04798200 1.19301800 -1.82596800

H 6.78873100 0.46112400 -0.33092100

H 6.88023800 -1.02709700 -1.27526500

H 5.13886600 -0.15779800 -2.67272200

H 3.78795800 -3.14562000 -1.22867900

H 4.68111300 0.66326400 1.60527700

H 2.32281100 -2.45050500 0.50735900

H 2.20158400 -2.13625000 2.79823300

H 7.31744200 0.52435700 -2.02448900

O -0.28191400 1.23577500 3.12031800

H -3.69574200 3.87320200 -2.11942400

C -1.53315300 0.62516800 3.32489600

H -2.32719300 1.21422400 2.86102600

H -1.54373300 -0.38541100 2.91346500

H -1.70224400 0.58533100 4.39806500

*Model 3: Phosphate Clamp*

1 1

P 0.24294900 1.24154600 0.93604400

O 1.53059700 1.97659900 0.71142500

O 0.17565800 -0.13937500 0.36282800

O -0.98349400 2.12102100 0.42100000

C -0.94639700 2.60196400 -0.89984800

H -0.42584300 1.89924600 -1.55674600

H -0.42490300 3.56166600 -0.92645100

C -2.35087600 2.76649600 -1.38786700

H -2.32666200 3.30312000 -2.33998100

O -2.93373000 1.49676800 -1.59355900

C -4.03692200 1.30006600 -0.76542000

H -4.96876700 1.38705300 -1.33309700

N -4.01072100 -0.04612400 -0.28407700

C -2.93216400 -0.74720900 0.17689300

H -1.92842000 -0.33732800 0.17874100

N -3.25076000 -1.94145000 0.56300800

C -4.59601900 -2.03411800 0.35444000

C -5.50650700 -3.09027700 0.58668400

O -5.30159500 -4.20066600 1.04491800

N -6.79490300 -2.73058900 0.21277000

H -7.48666500 -3.45419900 0.34971800

C -7.17337800 -1.53719300 -0.30995300

N -8.45864600 -1.39442700 -0.63408400

H -9.15569100 -2.06294900 -0.36187300

H -8.76326400 -0.48640500 -0.93627100

N -6.33843100 -0.55971500 -0.51836300

C -5.08235600 -0.86083600 -0.17745400

C -3.25797400 3.49513600 -0.40778400

H -2.68009300 4.12227300 0.26966100

C -3.92957400 2.35305000 0.31316000

H -3.27392000 1.98502300 1.10281500

H -4.89382100 2.63245600 0.73476500

O -4.15610300 4.35128300 -1.04881900

C 5.37106400 -2.04116100 -2.52515900

N 4.33473000 -2.18635700 -1.50262200

N 5.89022700 -1.23553100 0.77085000

N 2.49492400 0.01525000 -1.12715600

Pt 4.18405000 -0.61439200 -0.19163400

N 4.02148300 0.96393600 1.08892600

C 4.95202500 2.05972600 0.83515000

H 6.34269800 -1.94147400 -2.04724500

H 5.17853100 -1.14443400 -3.10822900

H 4.49056900 -3.04844700 -0.98827300

H 5.94940700 -0.90958600 1.73070500

H 5.97406900 -2.24590000 0.82560500

H 1.63922600 -0.31494000 -0.64556400

H 2.41214700 1.02887400 -1.07569300

H 4.13014300 0.64708400 2.04741900

H 5.98119800 1.71190100 0.90427300

H 4.78609300 2.44612300 -0.16800200

H 3.05364100 1.33921200 1.03222600

H 2.44035100 -0.24045300 -2.10695400

H 6.72543600 -0.89665800 0.30151600

H 3.44026900 -2.33423400 -1.96031600

H 5.38597300 -2.90780500 -3.18498700

H 4.80123200 2.86413500 1.55550800

O -0.17764400 1.23529400 2.46789400

C 0.47610000 0.33210500 3.32644600

H 1.53493900 0.58230200 3.42091100

H 0.00549100 0.41706300 4.30229400

H 0.37359600 -0.68973100 2.95871800

H -4.68410100 3.87756600 -1.69438700

*Model 4: Phosphate Arginine-Fork*

1 1

C 2.71541100 -0.40610700 2.54260700

N 1.51756400 -0.74778900 1.78476300

N 3.41592500 -0.02734400 -0.28900600

N -0.09509000 -2.09961100 -0.20038900

Pt 1.70404100 -1.12898600 -0.21512500

N 1.84115800 -1.53377900 -2.22688600

C 2.51976900 -2.78986600 -2.55441300

H 3.08391700 0.56064700 2.20358700

H 3.48414500 -1.16631800 2.41439500

H 0.87866300 0.06866900 1.88036300

H 3.87063600 0.00384700 -1.19513100

H 3.11397900 0.93786200 -0.04564000

H -0.53896400 -2.13051900 0.71317200

H -0.71854700 -1.56807600 -0.81387100

H 2.30993900 -0.77443200 -2.71269200

H 3.53136500 -2.76691600 -2.15703600

H 1.98740300 -3.61975300 -2.09639200

H 0.91034900 -1.54836400 -2.63609000

H -0.04755100 -3.05838000 -0.53033000

H 4.12271100 -0.33721500 0.36861200

H 1.07777200 -1.54595700 2.23088900

H 2.47795800 -0.32294100 3.60297200

H 2.55662600 -2.93741800 -3.63298400

P 0.76275000 2.48975900 0.80660400

O 2.16639300 2.29951400 0.33351800

O 0.28001900 1.64456000 1.94457000

O -0.26917000 2.31300700 -0.40573400

C 0.13119500 1.84522900 -1.66629700

H 0.93359900 1.11301900 -1.55664000

H 0.49564900 2.67402700 -2.27881600

C -1.04788600 1.21659900 -2.34112600

H -0.74973200 0.92389500 -3.35169100

O -1.46198900 0.06326600 -1.62832600

C -2.80094000 0.17351300 -1.21062800

H -3.45363500 -0.41641300 -1.85426200

N -2.91188600 -0.42506600 0.08936000

C -2.11378300 0.01838800 1.08210500

H -1.43655100 0.83198800 0.84803100

C -2.12854400 -0.55636100 2.29416200

H -1.48919500 -0.19409000 3.08607900

C -3.04671600 -1.62991900 2.47160600

N -3.12968000 -2.23700200 3.64690000

H -3.78341500 -2.99130400 3.76548600

H -2.56644100 -1.95082400 4.42689200

N -3.82392100 -2.05636800 1.50206100

C -3.79426900 -1.48547200 0.28885700

O -4.49990900 -1.85104600 -0.63897100

C -2.27021600 2.12145000 -2.42122200

H -1.98396400 3.16740900 -2.31965300

C -3.10723100 1.64909300 -1.25974300

H -2.76058500 2.13023000 -0.34422800

H -4.16718200 1.85629800 -1.39744500

O -2.92503600 2.02377100 -3.64985800

O 0.47429000 4.02334500 1.12758400

C 1.02144400 4.55622000 2.30931500

H 2.11224900 4.51511300 2.28566600

H 0.70633300 5.59454600 2.37248700

H 0.65537600 4.01179500 3.18106800

H -3.25404000 1.13487500 -3.79736400

*Model 4: Phosphate Clamp*

1 1

C -3.54082500 1.33540200 -2.27172400

N -2.60749100 1.37198400 -1.15107600

N -3.61380900 -0.61055500 0.69511700

N -0.27052100 -0.31790200 -1.64385100

Pt -1.95393300 -0.45969200 -0.50363600

N -1.31493700 -2.28177200 0.15877200

C -1.83466400 -3.41928800 -0.59230300

H -4.41397900 0.74230100 -2.00781400

H -3.05958300 0.87180100 -3.12964600

H -3.02571700 1.85074200 -0.35451400

H -3.93124100 -1.56707300 0.81838800

H -3.40710600 -0.23806200 1.62659200

H 0.25160600 0.55251000 -1.51325300

H 0.39305700 -1.06147100 -1.37552100

H -1.53825200 -2.39403700 1.14267500

H -2.92258700 -3.43253500 -0.56226900

H -1.52181400 -3.33341000 -1.63088800

H -0.28017300 -2.30406800 0.10196200

H -0.47512300 -0.40998400 -2.63352000

H -4.41767400 -0.10091000 0.34191200

H -1.82375900 1.98699800 -1.37253400

H -3.86277300 2.34142300 -2.54079500

H -1.45593900 -4.35656400 -0.18352000

P 2.45446900 -1.90081500 0.52599000

O 1.33525400 -2.21601800 -0.43131800

O 2.15972000 -1.84010400 1.97612000

O 3.11544200 -0.50103400 0.12639700

C 3.33878200 -0.16849800 -1.21749700

H 2.64981300 -0.71993500 -1.86164800

H 4.36202800 -0.42799300 -1.50076000

C 3.11890600 1.30332800 -1.39105600

H 3.28262400 1.55831700 -2.44095400

O 1.78726900 1.62884000 -1.04109800

C 1.73957600 2.42995500 0.11408100

H 1.45518100 3.43930800 -0.18046800

N 0.66885500 1.98470100 0.96433800

C 0.80499500 0.96455600 1.83624800

H 1.76073300 0.46045600 1.88057700

C -0.22594900 0.55923700 2.59679600

H -0.11968500 -0.26920600 3.28052800

C -1.44241200 1.25993100 2.43439600

N -2.52372400 0.90956900 3.14672500

H -3.28510600 1.57018100 3.17161400

H -2.40307000 0.33430900 3.96384900

N -1.59533600 2.21154500 1.54486000

C -0.58093000 2.54496200 0.73589900

O -0.72303200 3.30290500 -0.21766100

C 3.98884700 2.18568300 -0.51276000

H 4.93283400 1.69883200 -0.27181600

C 3.12496500 2.38861200 0.70869800

H 3.25640300 1.54011800 1.37635500

H 3.37254700 3.30560800 1.24048400

O 4.32529500 3.38309600 -1.14655400

O 3.67591900 -2.87273700 0.18546000

C 3.43059100 -4.25627900 0.13400200

H 2.63883000 -4.47732400 -0.58357800

H 4.35236400 -4.73871600 -0.18129500

H 3.14726800 -4.63834600 1.11694100

H 3.53855200 3.86670600 -1.40700700

1. Coordinates of the optimized molecules shown in figure 8, models 5, 6 and 7, using the BHandH functional.

*Model 5: Sulfate Arginine-Fork*

0 1

Pt 4.05082100 0.32116700 0.22510300

N 5.05533900 0.35332400 2.01241700

N 5.36888000 1.68296600 -0.55671800

N 3.02777200 0.29217300 -1.53362200

H 4.83346100 -0.42858900 2.62064400

H 4.84241700 1.19550100 2.53987500

H 5.89164900 2.13914900 0.18470900

H 4.84666100 2.43979200 -0.98932300

H 2.96013500 -0.65575900 -1.89864800

H 6.06389500 0.32523100 1.89416100

H 3.41839400 0.87229100 -2.26773800

H 2.05206000 0.58228600 -1.38755900

N 2.72423000 -1.03622300 0.97538400

H 3.22055200 -1.77883200 1.45833200

H 2.23261600 -1.49850400 0.19684100

C 1.71585400 -0.46053600 1.86239000

H 2.19035500 0.07502600 2.68193200

H 1.10779700 0.23554700 1.28530700

C 6.31516800 1.12658200 -1.52478100

H 6.96312200 1.90677600 -1.92218100

H 6.91955100 0.36505800 -1.03914300

H 1.08565400 -1.25059700 2.27533600

H 5.77197300 0.66216100 -2.34456800

C -3.29419200 -1.80102700 -0.81893400

C -2.26336900 -1.08139100 0.01875400

C -2.67721000 -1.09938100 1.46718900

C -4.06356800 -0.54334400 1.59538600

C -5.00112900 -1.28500800 0.67524300

C -6.40723600 -0.75958500 0.73614900

N -0.93319800 -1.63856200 -0.07486200

O -1.78666500 -0.35106600 2.23374100

O -4.55046800 -1.22788300 -0.65003400

O -6.50340300 0.62955900 0.94942700

S -0.00306200 -1.13003100 -1.34082000

O -0.71798000 -1.35314200 -2.58623400

O 0.25172100 0.28683600 -1.06912000

O 1.21462800 -1.94228500 -1.21019300

S -6.40390300 1.65508100 -0.28655900

O -7.15517400 2.79343700 0.21424600

O -4.98889200 1.93284800 -0.46876800

O -7.02475900 0.97143500 -1.41041400

H -3.03233200 -1.73925800 -1.87392800

H -2.20414300 -0.03506800 -0.29708400

H -2.67308100 -2.14772100 1.80662700

H -4.06036100 0.51135900 1.30804000

H -5.04611000 -2.34525900 0.97163700

H -6.93919800 -1.02969200 -0.17789600

H -6.91620500 -1.20289200 1.59099900

H -0.90642800 -2.65142200 -0.01556200

H -0.90287100 -0.58602200 1.93258400

H -3.31982600 -2.86400400 -0.52947100

H -4.41146100 -0.61987600 2.62834000

*Model 5: Sulfate Clamp*

0 1

Pt -4.06642600 0.21544800 -0.32194500

N -5.08740200 -0.42301500 -1.98193600

N -5.41311800 1.74108900 -0.06553300

N -3.02143400 0.84450600 1.30767800

H -4.78467300 -1.33011200 -2.32284700

H -4.97314700 0.22280700 -2.75843500

H -5.96050400 1.87548200 -0.91006800

H -4.90757700 2.61621300 0.04127900

H -2.95941000 0.10378000 2.00314800

H -6.08496200 -0.51475200 -1.81452000

H -3.40149900 1.66266100 1.77076400

H -2.04473600 1.04920600 1.05868200

N -2.71511000 -1.29690300 -0.55560800

H -3.19921800 -2.17090200 -0.73708700

H -2.21372700 -1.44424300 0.33278500

C -1.72266100 -1.05931200 -1.60225100

H -2.21241100 -0.84599600 -2.55022100

H -1.11588600 -0.20154500 -1.31365500

C -6.33005600 1.56627100 1.06207700

H -6.99791400 2.42218700 1.15094800

H -6.91546800 0.66292800 0.91330500

H -1.08816700 -1.93929600 -1.72405600

H -5.76303200 1.45994100 1.98390200

C 3.30203800 -1.38332600 1.40291800

C 2.27315900 -1.00607600 0.36316600

C 2.68311100 -1.54037900 -0.98405500

C 4.07237400 -1.07535800 -1.30234300

C 5.00757800 -1.44709400 -0.17844800

C 6.41630200 -0.98669500 -0.42208300

N 0.94115400 -1.48908800 0.64882500

O 1.79409300 -1.10754200 -1.96536100

O 4.56138600 -0.91727800 1.03925500

O 6.51874900 0.23943300 -1.10755800

S 0.00865400 -0.55573700 1.64119800

O 0.72076700 -0.30999500 2.88369200

O -0.24602000 0.66500300 0.87227400

O -1.20897700 -1.36068400 1.81095900

S 6.43007300 1.63322800 -0.30872000

O 7.19967300 2.51536300 -1.16953000

O 5.01817300 1.97330800 -0.24823800

O 7.03701600 1.37831400 0.98828600

H 3.04291900 -0.94487900 2.36524700

H 2.21829200 0.08431500 0.28491600

H 2.67165200 -2.64084600 -0.92916000

H 4.07658400 0.01266000 -1.40905200

H 5.04518200 -2.54364900 -0.07760800

H 6.95024600 -0.92320500 0.52763800

H 6.91924600 -1.70463600 -1.06862000

H 0.91277500 -2.45464100 0.96045900

H 0.91028400 -1.21401300 -1.59834900

H 3.32091100 -2.47939100 1.51519600

H 4.41726600 -1.51654100 -2.24064600

*Model 6: Sulfate Arginine-Fork*

0 1

Pt 3.40099600 -0.62261100 -0.02127800

N 2.90747100 1.18780500 0.77268500

N 1.64275300 -0.66606500 -1.05241400

N 3.89833300 -2.44511400 -0.81968600

H 3.71242300 1.79019500 0.91259400

H 2.46518500 1.07470700 1.68060900

H 0.90853100 -0.10993100 -0.59100300

H 1.26979900 -1.61048800 -1.06944000

H 4.79031700 -2.41940300 -1.30524800

H 2.24542000 1.73204500 0.19358900

H 3.22789600 -2.79232200 -1.49879200

H 3.96335100 -3.16184100 -0.10196600

N 5.17878700 -0.58104800 1.00942700

H 5.72089900 0.22092000 0.69975300

H 5.74763000 -1.38229400 0.75395800

C 5.05945000 -0.55584800 2.46777200

H 4.53940100 0.34598800 2.78183200

H 4.48742200 -1.42008200 2.79444200

C 1.73837800 -0.15908000 -2.41983000

H 0.76767900 -0.21862100 -2.91235500

H 2.05517000 0.88131900 -2.38643900

H 6.04377800 -0.57180000 2.93365600

H 2.46814100 -0.73354800 -2.98654000

C -4.41206900 0.84316200 -0.49700600

C -3.98636900 -0.18014000 0.52664700

C -3.79512900 0.49413800 1.85948100

C -2.81486000 1.61869500 1.70544200

C -3.25223200 2.54717000 0.59703200

C -2.25468500 3.63446900 0.32840600

N -4.90885300 -1.27442300 0.69702100

O -3.33212800 -0.41980700 2.80233000

O -3.46354900 1.85567600 -0.60001700

O -0.90689900 3.20766700 0.37832600

S -4.76227000 -2.54741500 -0.36362500

O -4.82056400 -2.01058200 -1.72383100

O -3.46223200 -3.13223100 -0.05511800

O -5.90295400 -3.39041200 -0.02666000

S -0.26633600 2.39724800 -0.83616200

O 1.15307700 2.71964400 -0.70529000

O -0.51125000 0.98590600 -0.55799400

O -0.88005800 2.89875500 -2.04432500

H -4.51518100 0.37508900 -1.47442600

H -3.02218300 -0.60419000 0.22881700

H -4.77034800 0.89752100 2.17758000

H -1.83901500 1.20067900 1.44752000

H -4.19324400 3.04371000 0.88343600

H -2.45578800 4.08481300 -0.64473000

H -2.32302300 4.39778500 1.10143900

H -5.88047500 -0.98714900 0.74619700

H -3.83800900 -1.22877900 2.66284300

H -5.38832200 1.26582200 -0.20806800

H -2.71932500 2.17578600 2.64075200

*Model 6: Sulfate Clamp*

0 1

Pt 4.35607800 -0.34148100 -0.06780200

N 2.88330200 1.00380800 -0.48865100

N 2.98714700 -1.44589600 0.96272600

N 5.82479400 -1.70362500 0.36933900

H 3.15121400 1.74017300 -1.13218100

H 2.56922900 1.44704100 0.37272000

H 2.27306000 -0.80940200 1.34189300

H 3.41472500 -1.88184300 1.77342600

H 6.13053900 -2.20099600 -0.46227100

H 2.03896300 0.55880000 -0.86830900

H 5.53801700 -2.41790800 1.03134500

H 6.64599300 -1.26609600 0.77652600

N 5.70936100 0.79957100 -1.10881600

H 5.32576500 1.01264000 -2.02539000

H 6.54729900 0.26311300 -1.31241400

C 6.10001900 2.04271700 -0.44265000

H 5.21848700 2.65203200 -0.25709000

H 6.56277100 1.80825100 0.51226700

C 2.30367800 -2.46358800 0.16531900

H 1.60913800 -3.02946300 0.78718200

H 1.74348300 -1.97153000 -0.62761700

H 6.80017400 2.60620700 -1.05813100

H 3.02788700 -3.14554300 -0.27482400

C -4.36918700 0.50094100 -1.41143800

C -5.09717700 0.43653900 -0.08960000

C -4.92477600 1.73658900 0.65292100

C -3.46398200 2.05198700 0.77607500

C -2.82118500 2.02992300 -0.58888500

C -1.34907900 2.31267500 -0.54083400

N -6.50869200 0.16702800 -0.20116600

O -5.50804700 1.65323600 1.91404500

O -3.02118400 0.79675000 -1.21894500

O -0.69467100 1.77579400 0.59261600

S -6.96191000 -1.42909100 -0.31932500

O -6.22152000 -2.02736900 -1.43108500

O -6.59396300 -2.00927600 0.96700300

O -8.39734800 -1.34590900 -0.56026400

S -0.18880500 0.26613000 0.61177900

O 0.93362600 0.35428900 1.54796500

O -1.27796200 -0.55653400 1.08292400

O 0.25662700 -0.03074400 -0.74800300

H -4.43745400 -0.45708700 -1.92346100

H -4.66029000 -0.35711100 0.52491400

H -5.41685300 2.52866000 0.06526300

H -2.98294000 1.29604000 1.40231300

H -3.27105700 2.81372700 -1.21912400

H -0.86899200 1.94389600 -1.44858700

H -1.18812500 3.38598100 -0.45827400

H -6.96944800 0.70270500 -0.92879800

H -6.35359700 1.20659300 1.79154300

H -4.83576300 1.27034000 -2.04750400

H -3.32060800 3.02888400 1.24435900

*Model 7: Sulfate Arginine-Fork*

0 1

C -3.19593700 0.82951700 -0.12676100

C -3.27274400 -0.65081800 0.21975300

C -4.48858400 -1.26289400 -0.41260600

C -5.72420200 -0.56703300 0.09356600

C -5.47162800 0.93450300 0.20346200

C -6.73145400 1.71828900 -0.11353500

O -2.16613700 -1.37149700 -0.30048200

O -4.57156900 -2.62055600 -0.11245900

O -4.40956200 1.29677400 -0.62911400

O -7.69120100 1.44802200 0.63246500

O -6.71463400 2.52424000 -1.05232800

S -0.84898300 -1.38195300 0.60352600

O 0.09034500 -2.10658000 -0.23892300

O -0.50372200 0.02656000 0.78371400

O -1.19928700 -2.04958100 1.83727700

H -2.44320800 0.97039800 -0.90210800

H -3.31557600 -0.82700800 1.29930500

H -4.40155500 -1.12169500 -1.49660400

H -5.98244300 -0.98320200 1.06895400

H -5.19947200 1.18544200 1.23910400

H -3.74942400 -3.03696000 -0.38321300

H -6.55988300 -0.77433900 -0.57697400

H -2.89966000 1.41494700 0.75057900

Pt 3.20568600 0.31564300 -0.07446300

N 4.90174900 -0.63634900 0.56577100

N 3.77806400 1.97287600 0.99155500

N 1.50254800 1.27092200 -0.66060900

H 4.98736300 -1.58451900 0.21674300

H 4.93309000 -0.70769000 1.57864700

H 4.49626000 1.73723800 1.66982300

H 2.99688200 2.28705700 1.56106600

H 1.34648700 1.22135200 -1.66242800

H 5.74378200 -0.15001700 0.27137900

H 1.48307400 2.25888100 -0.43017000

H 0.69181600 0.83777900 -0.18706000

N 2.55351400 -1.28055200 -1.17850300

H 2.48947200 -0.97776100 -2.14595600

H 1.59042300 -1.48465300 -0.87290800

C 3.28532700 -2.53968300 -1.14740100

H 4.28810600 -2.40909100 -1.55203800

H 3.33920600 -2.90718000 -0.12373200

C 4.26005700 3.08448700 0.17074300

H 4.50800600 3.94350300 0.79292100

H 5.14156800 2.76916300 -0.38107000

H 2.77250700 -3.28897700 -1.74982800

H 3.49342400 3.37289900 -0.54476600

*Model 7: Sulfate Clamp*

0 1

C -2.77572200 -0.74193600 -0.50002400

C -3.01560600 0.73564400 -0.22716900

C -4.02978700 0.90048800 0.86625800

C -5.32161500 0.24465200 0.45843200

C -5.03827300 -1.08228700 -0.24396000

C -6.10513800 -2.10588400 0.09591200

O -1.83902800 1.37935900 0.24067000

O -4.26266100 2.24924600 1.12431900

O -3.75896800 -1.53342400 0.09153800

O -7.25045800 -1.77423200 -0.26319200

O -5.77536000 -3.13985900 0.69044500

S -0.85751500 1.94143500 -0.88607700

O 0.27982600 2.39708200 -0.09067700

O -0.53565100 0.79204800 -1.72558900

O -1.57025800 2.99304900 -1.57409000

H -1.81838800 -1.02826700 -0.06524100

H -3.36888500 1.27371900 -1.11249500

H -3.63090800 0.40883900 1.76161100

H -5.86003000 0.92164700 -0.20742100

H -5.07754300 -0.93607900 -1.33291000

H -3.42016200 2.66593100 1.32210100

H -5.94716300 0.09560700 1.34023400

H -2.73438700 -0.93192500 -1.57833900

Pt 2.99474500 -0.36085500 0.17606400

N 3.72582800 -1.49809300 1.71672200

N 4.19126200 -1.37207700 -1.15072900

N 2.26382300 0.77496000 -1.34953500

H 3.21172300 -1.38680500 2.58490700

H 4.69265900 -1.26669400 1.92695600

H 4.88412400 -1.92512600 -0.65538500

H 4.74463900 -0.69936300 -1.67414100

H 1.31509300 0.49071600 -1.62206600

H 3.69418400 -2.49196800 1.50814900

H 2.84044200 0.78089700 -2.18386500

H 2.15477700 1.74492700 -1.06135500

N 1.79424300 0.65376900 1.48208700

H 1.22358300 -0.00845600 1.99938400

H 1.12745500 1.22731000 0.94813500

C 2.49088200 1.53441200 2.41629000

H 3.21038800 0.97181500 3.00809500

H 3.02870300 2.29511000 1.85555800

C 3.47646100 -2.24517400 -2.08250200

H 4.17024800 -2.71855300 -2.77605200

H 2.94509000 -3.00890300 -1.52109100

H 1.78143300 2.01720000 3.08813400

H 2.75019300 -1.66341800 -2.64532400

1. Coordinates of the optimized molecules shown in figure 9 using the ONIOM method.

*Model A*

6 1 6 1 6 1

H-H_ -1 3.92740900 15.06683600 -0.63979800 L

O-O_3 -1 3.62610400 14.42561600 0.01690500 L

C-C_3 -1 2.83960100 15.11139200 0.99594600 L

H-H_ -1 1.98463800 15.61376300 0.52558300 L

H-H_ -1 3.44637800 15.87053900 1.51008200 L

C-C_3 -1 2.31976500 14.13214200 2.04227100 L

H-H_ -1 1.94804500 14.72810600 2.88526100 L

O-O_3 -1 1.22754200 13.34709200 1.51905100 L

C-C_3 -1 1.47513000 11.93560500 1.69620300 L

H-H_ -1 0.93465600 11.58492000 2.58218700 L

N-N_R -1 0.92773200 11.23393200 0.47103500 L

C-C_R -1 1.50156700 11.49776400 -0.73306000 L

H-H_ -1 2.33925300 12.17772100 -0.77703000 L

C-C_R -1 1.04858300 10.93503700 -1.88536500 L

H-H_ -1 1.52953800 11.17236800 -2.83400800 L

C-C_R -1 -0.06289800 10.04113700 -1.77588900 L

N-N_R -1 -0.55303200 9.45993000 -2.85469300 L

H-H_ -1 -1.33780300 8.83449800 -2.73570600 L

H-H_ -1 -0.14656300 9.64236500 -3.76134200 L

N-N_R -1 -0.62906600 9.77034200 -0.60303300 L

C-C_R -1 -0.16129300 10.34414000 0.53811100 L

O-O_R -1 -0.66237200 10.11769000 1.64940700 L

C-C_3 -1 3.38235700 13.14679000 2.56377900 L

H-H_ -1 4.39296200 13.45323700 2.28649900 L

C-C_3 -1 2.99295200 11.81337700 1.90941400 L

H-H_ -1 3.55135900 11.72558900 0.97470500 L

H-H_ -1 3.26855800 10.97479600 2.54439400 L

O-O_3 -1 3.29812700 13.14357400 4.00838300 L

P-P_3+5 -1 4.12726500 12.08345500 4.95304000 L

O-O_2 -1 4.27068800 12.77963800 6.30455400 L

O-O_2 -1 5.33075200 11.50744200 4.25766600 L

O-O_3 -1 3.01398600 10.88804400 5.09729500 L

C-C_3 -1 1.71796000 11.16046300 5.65177000 L

H-H_ -1 1.14403200 11.77949000 4.95347000 L

H-H_ -1 1.82621100 11.70245500 6.59895400 L

C-C_3 -1 0.98224000 9.85466900 5.90477200 L

H-H_ -1 0.12129300 10.08354500 6.54585900 L

O-O_3 -1 0.49954500 9.28756400 4.66950800 L

C-C_3 -1 0.94989800 7.92559600 4.53674000 L

H-H_ -1 0.15854200 7.23758400 4.85646200 L

N-N_R -1 1.21483200 7.70685300 3.08296200 L

C-C_R -1 2.20783500 8.27003000 2.33791100 L

H-H_ -1 2.91845600 8.93636600 2.80472600 L

N-N_R -1 2.17080900 7.95032400 1.06872000 L

C-C_R -1 1.08997400 7.08098300 0.97368000 L

C-C_R -1 0.55141700 6.40137000 -0.14972700 L

O-O_R -1 0.92796500 6.42878900 -1.32027700 L

N-N_R -1 -0.55170700 5.61748100 0.20825900 L

H-H_ -1 -1.00277000 5.09925000 -0.51820600 L

C-C_R -1 -1.06666600 5.50284600 1.48321200 L

N-N_R -1 -2.13132200 4.69980500 1.61793300 L

H-H_ -1 -2.59871100 4.18299200 0.88673200 L

H-H_ -1 -2.51928200 4.61746400 2.54682100 L

N-N_R -1 -0.56004600 6.13930000 2.53558400 L

C-C_R -1 0.51340400 6.90553200 2.20466400 L

C-C_3 -1 1.83335200 8.77400900 6.59458700 L

H-H_ -1 2.72692300 9.19276700 7.06380300 L

C-C_3 -1 2.18031500 7.81856500 5.44350500 L

H-H_ -1 3.08244300 8.19033200 4.95052800 L

H-H_ -1 2.37325000 6.80957300 5.80698800 L

O-O_3 -1 1.00968100 8.16531300 7.59717100 L

P-P_3+5 -1 1.46672800 6.96648700 8.63726700 L

O-O_2 -1 0.77579100 7.26006700 9.95113700 L

O-O_2 -1 2.94990800 6.69204800 8.57231300 L

O-O_3 -1 0.72381300 5.69955600 7.87950300 L

C-C_3 -1 -0.68120000 5.73028500 7.62024600 L

H-H_ -1 -0.90319000 6.49479800 6.86769700 L

H-H_ -1 -1.22421000 5.98033100 8.54087800 L

C-C_3 -1 -1.16018800 4.37199700 7.11640300 L

H-H_ -1 -2.24203600 4.31658700 7.28466800 L

O-O_3 -1 -0.91788600 4.23271600 5.70139700 L

C-C_3 -1 -0.11038100 3.06906600 5.44467000 L

H-H_ -1 -0.75895700 2.23834900 5.14429300 L

N-N_R -1 0.78584700 3.41359000 4.29104600 L

C-C_R -1 1.85444600 4.23826400 4.50598500 L

H-H_ -1 2.03505400 4.61685000 5.50010300 L

C-C_R -1 2.70171500 4.58967800 3.50208100 L

H-H_ -1 3.54830600 5.24449800 3.70788100 L

C-C_R -1 2.42004600 4.06224300 2.20268400 L

N-N_R -1 3.20212000 4.36284900 1.18247000 L

H-H_ -1 2.97013700 3.96969900 0.28174000 L

H-H_ -1 4.00174600 4.96493800 1.31403600 L

N-N_R -1 1.37924700 3.26472600 1.98330000 L

C-C_R -1 0.54260200 2.92500300 3.00075100 L

O-O_R -1 -0.43892000 2.18535200 2.83527200 L

C-C_3 -1 -0.46537000 3.18738800 7.80895400 L

H-H_ -1 -0.00130400 3.48617200 8.74985500 L

C-C_3 -1 0.57157300 2.72487800 6.77308700 L

H-H_ -1 1.49035000 3.28077500 6.96570700 L

H-H_ -1 0.79652100 1.66545000 6.87552400 L

O-O_3 -1 -1.46797400 2.19253400 8.07751200 L

P-P_3+5 -1 -1.13834900 0.68412500 8.61951600 L

O-O_2 -1 -2.12101300 0.31486000 9.71158400 L

O-O_2 -1 0.33992200 0.50421500 8.90934600 L

O-O_3 -1 -1.47985900 -0.17351400 7.25958400 L H-H_ 96 0.000

C-C_3 -1 -2.57832400 0.16958900 6.40508500 H

H-H_ -1 -2.44579700 1.17529400 5.99858900 H

H-H_ -1 -3.51717600 0.14422900 6.97419900 H

C-C_3 -1 -2.65899800 -0.83370900 5.26336100 H

H-H_ -1 -3.67389500 -0.78616700 4.85006300 H

O-O_3 -1 -1.73488900 -0.49005200 4.21062700 H

C-C_3 -1 -0.79109400 -1.55749000 4.00067100 H

H-H_ -1 -1.09087800 -2.13367700 3.11669900 H

N-N_R -1 0.53190100 -0.92726700 3.70445600 L H-H_ 102 0.00

C-C_R -1 1.36476700 -0.24624500 4.55684800 L

H-H_ -1 1.11165900 -0.15791700 5.60336900 L

N-N_R -1 2.41370600 0.27846500 3.97264700 L

C-C_R -1 2.28629100 -0.11415300 2.64506700 L

C-C_R -1 3.11861600 0.14787000 1.52477900 L

O-O_R -1 4.16277500 0.79520600 1.47448500 L

N-N_R -1 2.61149400 -0.44615400 0.36240300 L

H-H_ -1 3.12917400 -0.31658000 -0.48358800 L

C-C_R -1 1.45484300 -1.19564000 0.29067800 L

N-N_R -1 1.14122700 -1.67704000 -0.91907400 L

H-H_ -1 1.65249600 -1.54062100 -1.77956900 L

H-H_ -1 0.29114100 -2.21986100 -0.98281700 L

N-N_R -1 0.67739100 -1.44099600 1.34145800 L

C-C_R -1 1.15677700 -0.87066600 2.47962100 L

C-C_3 -1 -2.33001700 -2.26735400 5.69523300 H

H-H_ -1 -2.45356400 -2.40686800 6.77002400 H

C-C_3 -1 -0.86150400 -2.42096700 5.25681800 H

H-H_ -1 -0.23403900 -2.02826700 6.06085600 H

H-H_ -1 -0.59021500 -3.46118300 5.08615500 H

O-O_3 -1 -3.25014800 -3.14570800 5.00960100 H

P-P_3+5 -1 -3.10778100 -4.77675200 5.08787000 H

O-O_2 -1 -4.51588300 -5.34184900 5.09528800 H

O-O_2 -1 -2.11661600 -5.21065900 6.14941000 H

O-O_3 -1 -2.42143300 -5.10381200 3.63798400 H

C-C_3 -1 -2.94065800 -4.52658200 2.43177200 H

H-H_ -1 -2.77181400 -3.44661100 2.42644200 H

H-H_ -1 -4.01765900 -4.72099500 2.35436200 H

C-C_3 -1 -2.22600600 -5.17285800 1.25463700 L H-H_ 129 0.00

H-H_ -1 -2.83357600 -5.02825900 0.35399000 L

O-O_3 -1 -0.94874700 -4.54024900 1.05052000 L

C-C_3 -1 0.10787300 -5.50857300 1.15415100 L

H-H_ -1 0.41238900 -5.82384800 0.14741200 L

N-N_R -1 1.25305400 -4.80090100 1.77970200 L

C-C_R -1 1.47855200 -4.44170300 3.08767200 L

H-H_ -1 0.77934200 -4.74790500 3.85206100 L

N-N_R -1 2.58088900 -3.78160700 3.28089000 L

C-C_R -1 3.15092200 -3.69971200 2.01611600 L

C-C_R -1 4.33555400 -3.11876900 1.54896300 L

N-N_R -1 5.20624200 -2.47642500 2.34026300 L

H-H_ -1 6.04581100 -2.07647800 1.94708200 L

H-H_ -1 5.02193400 -2.39178600 3.33038100 L

N-N_R -1 4.59880300 -3.21992000 0.23882600 L

C-C_R -1 3.73195500 -3.85786200 -0.54074300 L

H-H_ -1 3.92439500 -3.95043700 -1.59915600 L

N-N_R -1 2.59207500 -4.44120100 -0.21822300 L

C-C_R -1 2.35194400 -4.32477100 1.10444100 L

C-C_3 -1 -1.94678700 -6.67144200 1.47385000 L

H-H_ -1 -2.60510600 -7.10402400 2.22734700 L

C-C_3 -1 -0.47775900 -6.68450700 1.92342500 L

H-H_ -1 -0.46546800 -6.52303600 3.00362800 L

H-H_ -1 0.01020900 -7.63503100 1.70975200 L

O-O_3 -1 -2.20324900 -7.32763200 0.21613300 L

P-P_3+5 -1 -1.82785900 -8.89091500 -0.10692000 L

O-O_2 -1 -3.01336000 -9.45657100 -0.88773000 L

O-O_2 -1 -1.31228200 -9.62692300 1.09976500 L

O-O_3 -1 -0.59010100 -8.69990200 -1.14431400 L

C-C_3 -1 -0.60071300 -7.73718400 -2.20961400 L

H-H_ -1 -0.65269800 -6.72475300 -1.80016700 L

H-H_ -1 -1.46777900 -7.90613300 -2.86125000 L

C-C_3 -1 0.68643000 -7.92408800 -2.99853600 L

H-H_ -1 0.57632900 -7.46419200 -3.98773700 L

O-O_3 -1 1.77131800 -7.26743400 -2.30767300 L

C-C_3 -1 2.79887200 -8.21822800 -1.98240000 L

H-H_ -1 3.59771000 -8.15161500 -2.73314700 L

N-N_R -1 3.39848800 -7.79480100 -0.67136300 L

C-C_R -1 2.93216300 -7.92838500 0.62047100 L

H-H_ -1 2.01454800 -8.46903100 0.80008200 L

N-N_R -1 3.69680700 -7.39705400 1.52832500 L

C-C_R -1 4.77182000 -6.89001400 0.80796100 L

C-C_R -1 5.92907300 -6.20444600 1.19617600 L

N-N_R -1 6.21359500 -5.89514700 2.46819800 L

H-H_ -1 7.06510200 -5.39849200 2.68870200 L

H-H_ -1 5.57699800 -6.15727800 3.20714300 L

N-N_R -1 6.78754000 -5.84705900 0.23146300 L

C-C_R -1 6.49957500 -6.15712700 -1.02994500 L

H-H_ -1 7.17763300 -5.87803200 -1.82242500 L

N-N_R -1 5.44926200 -6.79515200 -1.51235900 L

C-C_R -1 4.60240000 -7.14299900 -0.52071300 L

C-C_3 -1 1.05056400 -9.41365300 -3.13269700 L

H-H_ -1 0.19099600 -10.05191300 -2.92608600 L

C-C_3 -1 2.13065400 -9.58753700 -2.05419300 L

H-H_ -1 1.61324900 -9.83716500 -1.12584400 L

H-H_ -1 2.81903400 -10.39004100 -2.31203100 L

O-O_3 -1 1.46852700 -9.67116200 -4.48379200 L

P-P_3+5 -1 2.19218200 -11.10849600 -4.89908600 L

O-O_2 -1 1.84746300 -11.33819500 -6.35555500 L

O-O_2 -1 1.95129800 -12.17956700 -3.86193600 L

O-O_3 -1 3.77341200 -10.66321100 -4.75744000 L

C-C_3 -1 4.12904000 -9.44721300 -5.41769700 L

H-H_ -1 3.83067300 -8.61422200 -4.81603200 L

H-H_ -1 3.63450900 -9.39805800 -6.36528700 L

C-C_3 -1 15.39212900 -4.08655100 4.42379500 L

H-H_ -1 16.02064100 -3.95802900 5.28017200 L

O-O_2 -1 15.89781300 -3.31356400 3.34537000 L

P-P_3+q -1 15.15942700 -2.32196400 3.16532700 L

O-O_2 -1 16.51940600 -1.73965700 3.11777300 L

O-O_2 -1 14.19333400 -1.71121700 4.10511800 L

O-O_2 -1 14.63446900 -2.30180200 1.65486200 L

C-C_3 -1 15.15364300 -3.26724000 0.72166700 L

H-H_ -1 14.92795200 -4.27400500 1.07486800 L

H-H_ -1 16.23363700 -3.14635600 0.63657000 L

C-C_3 -1 14.51937300 -3.06439700 -0.64516900 L

H-H_ -1 15.14339400 -3.52918200 -1.40878200 L

O-O_3 -1 13.26307100 -3.80611400 -0.69722000 L

C-C_3 -1 12.16478800 -2.91014600 -0.60648100 L

H-H_ -1 11.54843000 -2.99824800 -1.50104700 L

N-N_R -1 11.22145900 -3.36645200 0.50842700 L

C-C_R -1 11.43853900 -3.08753800 1.83200700 L

H-H_ -1 12.30984600 -2.52102000 2.12871200 L

C-C_R -1 10.56114200 -3.45877100 2.77367400 L

C-C_3 -1 10.79011500 -3.15808700 4.22534800 L

H-H_ -1 10.86159700 -2.07971100 4.36674700 L

H-H_ -1 9.95787000 -3.54703500 4.81304400 L

H-H_ -1 11.71635400 -3.62941500 4.55262500 L

C-C_R -1 9.35687200 -4.16541300 2.42093900 L

O-O_R -1 8.49620800 -4.54549300 3.21354800 L

N-N_R -1 9.21991700 -4.40341500 1.06780300 L

H-H_ -1 8.32292800 -4.93656000 0.75162100 L

C-C_R -1 10.10543400 -4.03366300 0.07680300 L

O-O_R -1 9.89668000 -4.29026400 -1.09805400 L

C-C_3 -1 14.12712300 -1.62547400 -0.98926100 L

H-H_ -1 14.80070000 -0.93379900 -0.48375800 L

C-C_3 -1 12.72586500 -1.50401100 -0.38774600 L

H-H_ -1 12.71733000 -0.70964700 0.35848400 L

H-H_ -1 12.00983300 -1.26883900 -1.17591200 L

O-O_2 -1 14.02604600 -1.30896400 -2.36946000 L

P-P_3+q -1 13.26098400 -0.31823000 -2.53056100 L

O-O_2 -1 14.40849800 -0.00175600 -3.40952900 L

O-O_2 -1 12.87399300 0.69904700 -1.52834600 L

O-O_2 -1 12.05806300 -0.68798800 -3.51804200 L

C-C_3 -1 12.06618800 -1.96572200 -4.18264100 L

H-H_ -1 12.06376000 -2.76272000 -3.43889600 L

H-H_ -1 12.96126100 -2.04844200 -4.79936700 L

C-C_3 -1 10.83680300 -2.10152200 -5.06504000 L

H-H_ -1 11.01025500 -2.87358600 -5.81491800 L

O-O_3 -1 9.73224700 -2.61541000 -4.26011100 L

C-C_3 -1 8.80581000 -1.57474800 -3.98403400 L

H-H_ -1 7.82946100 -1.83952300 -4.39044900 L

N-N_R -1 8.54482300 -1.50625500 -2.47876900 L

C-C_R -1 9.38268500 -0.86587300 -1.60368100 L

H-H_ -1 10.27562600 -0.37992200 -1.96818700 L

C-C_R -1 9.09070300 -0.78257000 -0.29777600 L

C-C_3 -1 10.00134800 -0.08407200 0.66774000 L

H-H_ -1 10.11366700 0.95941600 0.37276000 L

H-H_ -1 9.57591500 -0.13373700 1.67055400 L

H-H_ -1 10.97756200 -0.56880600 0.66387700 L

C-C_R -1 7.88296000 -1.36517800 0.22640700 L

O-O_R -1 7.53355100 -1.33856700 1.40486300 L

N-N_R -1 7.10053200 -1.98820900 -0.72421200 L

H-H_ -1 6.17457300 -2.44529000 -0.37506200 L

C-C_R -1 7.37377400 -2.09039600 -2.07318100 L

O-O_R -1 6.61646400 -2.66455700 -2.83854100 L

C-C_3 -1 10.29898800 -0.79910800 -5.66225600 L

H-H_ -1 11.12137500 -0.09925400 -5.80876100 L

C-C_3 -1 9.37580800 -0.27700500 -4.55961300 L

H-H_ -1 9.72235000 0.70136700 -4.22762500 L

H-H_ -1 8.35991800 -0.18988800 -4.94685400 L

O-O_2 -1 9.52360900 -0.91710900 -6.84612200 L

P-P_3+q -1 8.81336800 0.13362000 -6.75041000 L

O-O_2 -1 9.44859200 -0.05842300 -8.07316400 L

O-O_2 -1 8.88956100 1.48518400 -6.15214300 L

O-O_2 -1 7.30377000 -0.36759000 -6.92683600 L

C-C_3 -1 7.04081900 -1.78236800 -6.98941400 L

H-H_ -1 7.38055100 -2.25685000 -6.06979400 L

H-H_ -1 7.57238400 -2.21410500 -7.83823900 L

C-C_3 -1 5.55075900 -2.02690100 -7.15714200 L

H-H_ -1 5.38853600 -3.02972900 -7.55315100 L

O-O_3 -1 4.92456500 -2.04915100 -5.83855700 L

C-C_3 -1 4.19715000 -0.84808600 -5.62552800 L

H-H_ -1 3.14596200 -1.08458000 -5.45503000 L

N-N_R -1 4.63979600 -0.26177100 -4.32858700 L

C-C_R -1 5.77764000 0.47883600 -4.24549200 L

H-H_ -1 6.37546600 0.65081200 -5.12813000 L

C-C_R -1 6.17987000 1.01264500 -3.06122800 L

H-H_ -1 7.09384900 1.60433900 -3.01277800 L

C-C_R -1 5.35404100 0.75660000 -1.92171300 L

N-N_R -1 5.68542700 1.24490900 -0.74077700 L

H-H_ -1 5.07643700 1.04180500 0.03857900 L

H-H_ -1 6.52200500 1.80013600 -0.63597100 L

N-N_R -1 4.24362700 0.03121800 -2.01295600 L

C-C_R -1 3.85823000 -0.49478500 -3.20686100 L

O-O_R -1 2.82989500 -1.17607600 -3.33146200 L

C-C_3 -1 4.78146200 -0.94977800 -7.92593300 L

H-H_ -1 5.44227200 -0.48295900 -8.65599700 L

C-C_3 -1 4.43023600 0.06118700 -6.83351500 L

H-H_ -1 4.87043600 1.02820000 -7.07681400 L

H-H_ -1 3.34763300 0.16200000 -6.76562100 L

O-O_2 -1 3.56529300 -1.35497300 -8.53731800 L

P-P_3+q -1 2.78574300 -0.12744300 -8.47777100 L

O-O_2 -1 2.68405800 -0.82920000 -9.77638500 L

O-O_2 -1 3.12093300 1.31465200 -8.51052900 L

O-O_2 -1 1.40055900 -0.41237200 -7.73035000 L

C-C_3 -1 1.17253500 -1.71228700 -7.15480800 L

H-H_ -1 1.93533900 -1.91696800 -6.40341300 L

H-H_ -1 1.22220300 -2.47021800 -7.93679600 L

C-C_3 -1 -0.19865500 -1.75916900 -6.50052900 L

O-O_3 -1 -0.08586600 -1.25974900 -5.13303400 L

C-C_3 -1 -0.63721600 0.04562900 -5.04408200 L

H-H_ -1 -1.45943000 0.04508600 -4.32754300 L

N-N_R -1 0.37815300 0.93491200 -4.41477700 L

C-C_R -1 1.46261600 1.56458900 -4.99033300 L

H-H_ -1 1.66302600 1.43896600 -6.04444200 L

N-N_R -1 2.16915800 2.28297600 -4.15256400 L

C-C_R -1 1.51242000 2.11963900 -2.93812700 L

C-C_R -1 1.80484700 2.65444900 -1.65553200 L

O-O_R -1 2.72592500 3.39730400 -1.32138100 L

N-N_R -1 0.87254800 2.22913100 -0.70101200 L

H-H_ -1 0.99543700 2.55264500 0.23748100 L

C-C_R -1 -0.20147800 1.39942100 -0.95132100 L

N-N_R -1 -0.97761800 1.11004400 0.10102900 L

H-H_ -1 -0.85313100 1.42321400 1.05329900 L

H-H_ -1 -1.76142300 0.49574800 -0.07119900 L

N-N_R -1 -0.47484100 0.90146900 -2.15387100 L

C-C_R -1 0.42192700 1.30588100 -3.09333300 L

C-C_3 -1 -1.27151300 -0.87548000 -7.14049200 L

H-H_ -1 -1.07146300 -0.77484600 -8.20755100 L

C-C_3 -1 -1.05523700 0.47412600 -6.45193400 L

H-H_ -1 -0.81552900 1.22949200 -7.19973900 L

H-H_ -1 -1.96290200 0.76169100 -5.92259800 L

O-O_2 -1 -2.62156300 -1.25475700 -6.91787300 L

P-P_3+q -1 -3.26603600 0.06928100 -6.95043200 L

O-O_2 -1 -3.98172100 -0.97065200 -7.72203900 L

O-O_2 -1 -3.00950800 1.36038500 -7.62953200 L

O-O_2 -1 -4.10327700 0.25006700 -5.59994900 L H-H_ 329 0.00

C-C_3 -1 -4.00353200 -0.75902100 -4.57617900 H

H-H_ -1 -2.96732500 -0.84048000 -4.24666900 H

H-H_ -1 -4.33327000 -1.71750600 -4.97694300 H

C-C_3 -1 -4.87709700 -0.38516600 -3.39074400 H

H-H_ -1 -5.08261100 -1.27460100 -2.79442300 H

O-O_3 -1 -4.11490500 0.48425600 -2.49864000 H

C-C_3 -1 -4.56962300 1.82456700 -2.61882500 H

H-H_ -1 -4.93493600 2.17356200 -1.65359500 H

N-N_R -1 -3.38808300 2.69291800 -2.88324700 L H-H_ 335 0.00

C-C_R -1 -2.87134900 2.81439500 -4.13579100 L

H-H_ -1 -3.31868500 2.27746300 -4.95836700 L

C-C_R -1 -1.79292800 3.60708000 -4.37444300 L

H-H_ -1 -1.39242100 3.69354300 -5.38459500 L

C-C_R -1 -1.23615200 4.29782000 -3.25238100 L

N-N_R -1 -0.18840300 5.08453600 -3.41176800 L

H-H_ -1 0.17437700 5.55400500 -2.59510600 L

H-H_ -1 0.22455400 5.20186600 -4.32653700 L

N-N_R -1 -1.74816900 4.17252800 -2.03144200 L

C-C_R -1 -2.82699300 3.37422200 -1.81300300 L

O-O_R -1 -3.33250700 3.23188200 -0.68978100 L

C-C_3 -1 -6.13751600 0.41949300 -3.72013600 H

H-H_ -1 -6.48707500 0.15184700 -4.71708400 H

C-C_3 -1 -5.62980800 1.86090200 -3.72277000 H

H-H_ -1 -5.79777800 2.30392400 -4.70413200 H

H-H_ -1 -6.16554100 2.43633800 -2.96809200 H

O-O_2 -1 -7.19943900 0.34245900 -2.77989200 H

P-P_3+q -1 -7.81332500 1.65400700 -2.92879000 H

O-O_2 -1 -9.17091300 1.06414700 -2.96875200 H

O-O_2 -1 -7.87957100 2.84820800 -3.79930700 H

O-O_2 -1 -7.86985600 2.37908900 -1.50283300 H

C-C_3 -1 -7.24857800 1.74240300 -0.37134000 H

H-H_ -1 -6.18414100 1.60814000 -0.56791600 H

H-H_ -1 -7.71154100 0.77006800 -0.20059800 H

C-C_3 -1 -7.42201400 2.60256000 0.86977500 L H-H_ 500 0.00

H-H_ -1 -7.28180500 1.99047800 1.76044100 L

O-O_3 -1 -6.33821300 3.57901400 0.92258200 L

C-C_3 -1 -6.82388500 4.86583700 0.57074800 L

H-H_ -1 -6.66625700 5.55442000 1.40141100 L

N-N_R -1 -5.95183600 5.41033000 -0.50791600 L

C-C_R -1 -5.98394400 5.16085100 -1.86463300 L

H-H_ -1 -6.72188600 4.48428200 -2.27181100 L

N-N_R -1 -5.06925900 5.80277400 -2.54674000 L

C-C_R -1 -4.38043500 6.52727100 -1.58123400 L

C-C_R -1 -3.27815100 7.41489600 -1.70730300 L

O-O_R -1 -2.67004000 7.74799800 -2.72392100 L

N-N_R -1 -2.89580600 7.93358500 -0.46500200 L

H-H_ -1 -2.12402900 8.56932200 -0.45052400 L

C-C_R -1 -3.49751800 7.63829200 0.74120900 L

N-N_R -1 -2.97960600 8.24113100 1.81993100 L

H-H_ -1 -2.19365300 8.87435400 1.84284800 L

H-H_ -1 -3.40870100 8.02323900 2.70762700 L

N-N_R -1 -4.53097200 6.80883600 0.85762200 L

C-C_R -1 -4.91609100 6.29560800 -0.34169200 L

C-C_3 -1 -8.69750000 3.44673000 0.92053100 L

H-H_ -1 -9.49194300 2.93966200 0.37313400 L

C-C_3 -1 -8.29321400 4.71785600 0.17018600 L

H-H_ -1 -8.93854200 4.84843800 -0.69741600 L

H-H_ -1 -8.39407500 5.57825400 0.83114000 L

O-O_3 -1 -9.14761400 3.83918100 2.20912300 L

H-H_ -1 -9.94890600 4.36730200 2.21533400 L

N-N_3 -1 -8.37479600 -16.83540400 8.08931200 H

C-C_3 0 -8.56311700 -15.37641100 7.95075600 H

C-C_3 0 -8.02344500 -14.88578400 6.63592300 H

C-C_3 0 -8.25958600 -13.40256200 6.44625100 H

C-C_3 0 -7.40987500 -12.51378100 7.32494200 H

C-C_3 0 -7.78216200 -11.05590000 7.17604300 H

C-C_3 0 -6.96805700 -10.12124000 8.02717700 H

N-N_3 0 -5.59050800 -9.97443600 7.53375600 H

N-N_3 0 -5.10302100 -10.62932600 4.69144800 H

N-N_3 0 -5.57501600 -7.17372000 6.82680400 H

Pt-Pt4+2 0 -5.34829600 -8.91301800 5.78445500 H

N-N_3 0 -5.06742200 -7.82483500 4.07809600 H

C-C_3 0 -6.22825800 -7.61251600 3.21115800 H

C-C_3 0 -7.31053200 -6.85574500 3.93135300 H

C-C_3 0 -8.47617700 -6.50129900 3.03908700 H

C-C_3 0 -8.14104900 -5.53104700 1.93035200 H

C-C_3 0 -9.37850000 -5.08055200 1.19560600 H

C-C_3 0 -9.07112000 -4.15736700 0.04930800 H

N-N_3 0 -10.29402600 -3.70898600 -0.62343200 H

N-N_3 0 -9.42305200 -0.98098400 -1.18472800 H

N-N_3 0 -10.69805700 -4.12252800 -3.46318300 H

Pt-Pt4+2 0 -10.05697000 -2.55537900 -2.30655500 H

N-N_3 0 -9.85846800 -1.40702100 -3.97644600 H

C-C_3 0 -8.76531000 -1.76656900 -4.87979300 H

C-C_3 0 -8.89063400 -1.14971800 -6.25278900 H

C-C_3 0 -9.18588000 0.33937500 -6.30309500 H

C-C_3 0 -10.65426800 0.68136500 -6.15439000 H

C-C_3 0 -10.98987700 2.10919400 -6.51029300 H

C-C_3 0 -10.39860000 3.14673400 -5.59168700 H

N-N_3 0 -5.82513000 11.41143300 0.12618000 H

C-C_3 0 -6.29634900 10.25569600 0.91725600 H

C-C_3 0 -6.90733700 9.20634300 0.03508600 H

C-C_3 0 -8.21902700 9.60806000 -0.59395800 H

C-C_3 0 -8.80444000 8.49415500 -1.42496400 H

C-C_3 0 -10.14350800 8.85236900 -2.02689300 H

C-C_3 0 -10.69486500 7.77013500 -2.91654600 H

N-N_3 0 -11.04336900 6.55615000 -2.15990400 H

N-N_3 0 -9.09472400 5.20008000 -3.86496500 H

N-N_3 0 -10.84890500 2.99684900 -4.19811500 H

Pt-Pt4+2 0 -10.95140700 4.75750000 -3.15837100 H

N-N_3 0 -12.80033400 4.22898100 -2.45071600 H

H-H_ 0 -8.74893700 -17.18018200 8.97245200 H

H-H_ 0 -7.38761800 -17.08993200 8.05945000 H

H-H_ 0 -8.06551500 -14.91478600 8.80138100 H

H-H_ 0 -9.63231100 -15.18644800 8.03261300 H

H-H_ 0 -6.95247300 -15.10310200 6.56762400 H

H-H_ 0 -8.51292100 -15.43135500 5.82451300 H

H-H_ 0 -9.31826300 -13.18028000 6.62101400 H

H-H_ 0 -8.06716100 -13.15015500 5.40022600 H

H-H_ 0 -7.51876300 -12.79135500 8.37871600 H

H-H_ 0 -6.35576200 -12.68795600 7.07572800 H

H-H_ 0 -8.83028600 -10.93403400 7.46141500 H

H-H_ 0 -7.71584600 -10.74296200 6.12811000 H

H-H_ 0 -7.41649500 -9.12879200 8.04082800 H

H-H_ 0 -6.93488200 -10.48608600 9.05697900 H

H-H_ 0 -5.03518100 -9.51534000 8.25036900 H

H-H_ 0 -4.85123400 -11.44433700 5.24179700 H

H-H_ 0 -5.94838100 -10.87535900 4.18347300 H

H-H_ 0 -5.12937800 -7.19076500 7.73833400 H

H-H_ 0 -5.16111900 -6.37771700 6.30568500 H

H-H_ 0 -4.71536400 -6.89073100 4.35445500 H

H-H_ 0 -5.89331900 -7.05597900 2.33188400 H

H-H_ 0 -6.60122500 -8.57931900 2.86679200 H

H-H_ 0 -6.87927700 -5.94133000 4.35500500 H

H-H_ 0 -7.66902500 -7.46305000 4.76816300 H

H-H_ 0 -9.26567100 -6.06494200 3.65798800 H

H-H_ 0 -8.90051400 -7.41430300 2.60597200 H

H-H_ 0 -7.62638400 -4.65961900 2.35156500 H

H-H_ 0 -7.44608100 -5.98705900 1.21772200 H

H-H_ 0 -10.05401600 -4.57764200 1.89661900 H

H-H_ 0 -9.91558600 -5.95789400 0.81715200 H

H-H_ 0 -8.53330600 -3.27445400 0.39861700 H

H-H_ 0 -8.44187400 -4.65087300 -0.69198100 H

H-H_ 0 -10.87129800 -3.20446600 0.04519500 H

H-H_ 0 -9.37300500 -0.11637000 -1.76972500 H

H-H_ 0 -10.01965000 -0.78883100 -0.38671000 H

H-H_ 0 -10.61416200 -3.94678900 -4.45962100 H

H-H_ 0 -11.67801700 -4.33483300 -3.29890200 H

H-H_ 0 -9.70476000 -0.41857300 -3.69001100 H

H-H_ 0 -7.84665400 -1.45505800 -4.38305000 H

H-H_ 0 -8.70825900 -2.85234400 -4.97161300 H

H-H_ 0 -7.95416900 -1.35977700 -6.77580900 H

H-H_ 0 -9.66970600 -1.68019500 -6.81158800 H

H-H_ 0 -8.60049000 0.87827400 -5.55023100 H

H-H_ 0 -8.85239000 0.72040500 -7.27323300 H

H-H_ 0 -11.00097300 0.48839400 -5.13530600 H

H-H_ 0 -11.23549800 0.01727100 -6.80142700 H

H-H_ 0 -10.64210700 2.32573200 -7.52479800 H

H-H_ 0 -12.07781300 2.23404400 -6.52779700 H

H-H_ 0 -10.68846200 4.13503000 -5.94941100 H

H-H_ 0 -9.30968800 3.08954600 -5.57538500 H

H-H_ 0 -5.42131600 12.13451000 0.72030100 H

H-H_ 0 -5.10698600 11.13619300 -0.54402200 H

H-H_ 0 -5.43292800 9.87741100 1.45864700 H

H-H_ 0 -7.01534400 10.63786900 1.64032000 H

H-H_ 0 -7.06631000 8.32245100 0.65735300 H

H-H_ 0 -6.19303700 8.91242500 -0.74100600 H

H-H_ 0 -8.92764500 9.89020700 0.19158000 H

H-H_ 0 -8.09961700 10.48806300 -1.23610100 H

H-H_ 0 -8.10165000 8.23508600 -2.22596800 H

H-H_ 0 -8.88314500 7.60610400 -0.78601100 H

H-H_ 0 -10.03716000 9.75962200 -2.62739900 H

H-H_ 0 -10.86343800 9.08634300 -1.23552300 H

H-H_ 0 -9.96030200 7.51259300 -3.67873000 H

H-H_ 0 -11.59004800 8.09798100 -3.44176300 H

H-H_ 0 -10.43660300 6.49099200 -1.34791000 H

H-H_ 0 -8.58918700 5.89726900 -3.33003800 H

H-H_ 0 -9.12417400 5.53684200 -4.82238200 H

H-H_ 0 -10.23055100 2.31865600 -3.70312900 H

H-H_ 0 -13.12308200 4.79144600 -1.66971900 H

H-H_ 0 -13.51137900 4.30785100 -3.17235900 H

H-H_ 0 -6.58189500 11.84614300 -0.40099000 H

H-H_ 0 -11.96679200 6.69832200 -1.76224400 H

H-H_ 0 -8.53335000 4.32343400 -3.84538400 H

H-H_ 0 -12.81588000 3.26997100 -2.11614200 H

H-H_ 0 -11.77203800 2.57176100 -4.21058600 H

H-H_ 0 -10.17196500 -4.97346800 -3.28697800 H

H-H_ 0 -10.84588200 -4.53183900 -0.85276300 H

H-H_ 0 -8.48878900 -1.16104800 -0.83020000 H

H-H_ 0 -4.32312000 -8.24860000 3.53406000 H

H-H_ 0 -4.36421400 -10.53332700 4.00136800 H

H-H_ 0 -5.16548200 -10.89509400 7.46266300 H

H-H_ 0 -8.84227500 -17.34757800 7.34173100 H

H-H_ 0 5.22723200 -9.41687800 -5.56977900 L

H-H_ 0 15.56156600 -5.15089400 4.15489300 L

H-H_ 0 14.29379000 -4.06803200 4.68350300 L

H-H_ 0 -0.55196400 -2.81251500 -6.46903400 L

H-H_ 0 -10.74090600 -1.43673200 -4.47992800 H

H-H_ 0 -6.55450300 -6.95598600 6.98394000 H

Na-Na -1 18.60624800 1.12113900 6.32392500 L

Na-Na -1 16.74285000 4.01693900 -2.61520400 L

Na-Na -1 8.74588400 14.36022300 6.18065700 L

Na-Na -1 4.46601600 8.08706900 14.20986500 L

Na-Na -1 -0.09448200 -15.09767500 -4.56622600 L

Na-Na -1 -3.44902400 -12.17217000 1.43781600 L

Na-Na -1 -0.23219900 -1.26038000 12.61247800 L

Na-Na -1 10.76135600 3.41253500 -9.91780200 L

Na-Na -1 1.24393200 1.00191100 -12.66470000 L

Na-Na -1 -5.60348100 -0.94206500 -10.57478600 L

*Model B*

2 1 2 1 2 1

H-H_ -1 -3.18999200 -10.51369300 9.97359100 H

O-O_3 -1 -3.53779800 -9.98960200 9.24040400 H

C-C_3 -1 -4.96300900 -9.93489400 9.35522800 H

H-H_ -1 -5.25913600 -9.49781100 10.31743700 H

H-H_ -1 -5.39079500 -10.94552200 9.28852400 H

C-C_3 -1 -5.55583700 -9.09430800 8.23009100 H

H-H_ -1 -6.63546200 -9.28972500 8.21595200 H

O-O_3 -1 -5.34796400 -7.68655300 8.47097400 H

C-C_3 -1 -4.72125700 -7.05527500 7.33356500 H

H-H_ -1 -5.48847800 -6.55373300 6.73366000 H

N-N_R -1 -3.76048600 -6.01884200 7.87740000 L H-H_ 507 0.

C-C_R -1 -2.70066300 -6.44623100 8.61418400 L

H-H_ -1 -2.56348600 -7.50590700 8.76993900 L

C-C_R -1 -1.81053900 -5.57854700 9.16596600 L

H-H_ -1 -0.97469100 -5.95564600 9.75479500 L

C-C_R -1 -2.03439500 -4.18521700 8.93153300 L

N-N_R -1 -1.20893500 -3.28835100 9.43758700 L

H-H_ -1 -1.40062900 -2.31430000 9.24873100 L

H-H_ -1 -0.41617100 -3.58031400 9.99147900 L

N-N_R -1 -3.06403300 -3.75493200 8.20754400 L

C-C_R -1 -3.94357800 -4.63919800 7.66464600 L

O-O_R -1 -4.91540800 -4.27738200 6.98495600 L

C-C_3 -1 -4.97785200 -9.41020100 6.83792100 H

H-H_ -1 -4.41333800 -10.34476900 6.83375900 H

C-C_3 -1 -4.07963200 -8.20212500 6.53529600 H

H-H_ -1 -3.06938400 -8.44577000 6.87180100 H

H-H_ -1 -4.03888400 -8.00741300 5.46627900 H

O-O_3 -1 -6.09409300 -9.55966800 5.92927100 H

P-P_3+5-1 -5.91226900 -9.71128800 4.30209200 H

O-O_2- -1 -7.16307200 -10.44708400 3.82677700 H

O-O_2 -1 -4.54320300 -10.20145700 3.91595600 H

O-O_3 -1 -6.02682400 -8.13983800 3.84757500 H

C-C_3 -1 -7.19931500 -7.37280900 4.16107100 H

H-H_ -1 -7.24026900 -7.19189500 5.24079100 H

H-H_ -1 -8.09652500 -7.92717500 3.86053500 H

C-C_3 -1 -7.15967100 -6.04674600 3.41919300 H

H-H_ -1 -8.16339700 -5.60716200 3.48170500 H

O-O_3 -1 -6.22035000 -5.14067200 4.03334600 H

C-C_3 -1 -5.28551500 -4.65142300 3.05246500 H

H-H_ -1 -5.60176000 -3.66465200 2.69455100 H

N-N_R -1 -3.97351500 -4.50813000 3.75220300 L H-H_ 39 0.0

C-C_R -1 -3.18500500 -5.51346700 4.22746500 L

H-H_ -1 -3.48464800 -6.54227900 4.09086800 L

N-N_R -1 -2.11323500 -5.10057100 4.85615900 L

C-C_R -1 -2.18074900 -3.71528700 4.75906300 L

C-C_R -1 -1.30141400 -2.71440400 5.24790100 L

O-O_R -1 -0.25674700 -2.84968100 5.88280100 L

N-N_R -1 -1.75598200 -1.43140400 4.92111000 L

H-H_ -1 -1.20228200 -0.65614600 5.22469900 L

C-C_R -1 -2.90703200 -1.15081700 4.21387600 L

N-N_R -1 -3.16771500 0.14721500 4.00488600 L

H-H_ -1 -2.61914300 0.93511300 4.31871900 L

H-H_ -1 -4.01314200 0.36093800 3.49527300 L

N-N_R -1 -3.72964200 -2.08995800 3.75501800 L

C-C_R -1 -3.30184300 -3.34314200 4.06394300 L

C-C_3 -1 -6.76791200 -6.16484500 1.93573500 H

H-H_ -1 -6.86775300 -7.18787100 1.56500700 H

C-C_3 -1 -5.30862600 -5.68674600 1.92323000 H

H-H_ -1 -4.66615300 -6.54333900 2.14356800 H

H-H_ -1 -5.02926600 -5.28360900 0.95017300 H

O-O_3 -1 -7.64460500 -5.30338400 1.19857600 H

P-P_3+5-1 -7.65399300 -5.09430400 -0.43973600 H

O-O_2- -1 -9.10471500 -4.92812300 -0.83676000 H

O-O_2 -1 -6.75342200 -6.08031900 -1.14408400 H

O-O_3 -1 -6.88233200 -3.63397400 -0.49653700 H

C-C_3 -1 -7.39295200 -2.51331300 0.22844800 H

H-H_ -1 -7.28634300 -2.68761900 1.30471100 H

H-H_ -1 -8.45839600 -2.37752900 0.00186700 H

C-C_3 -1 -6.63964400 -1.24272900 -0.15414100 H

H-H_ -1 -7.27123200 -0.38746200 0.11306300 H

O-O_3 -1 -5.40351400 -1.13550200 0.58127300 H

C-C_3 -1 -4.29046200 -1.02695200 -0.32503100 H

H-H_ -1 -4.01016300 0.02743400 -0.42831700 H

N-N_R -1 -3.14635900 -1.76308000 0.30893000 L H-H_ 72 0.0

C-C_R -1 -3.15402500 -3.12986300 0.30343300 L

H-H_ -1 -3.97691800 -3.64933800 -0.16262900 L

C-C_R -1 -2.14862600 -3.85434300 0.86332800 L

H-H_ -1 -2.18182800 -4.94340500 0.83686100 L

C-C_R -1 -1.08071500 -3.11997100 1.46849200 L

N-N_R -1 -0.07224300 -3.76027800 2.03047700 L

H-H_ -1 0.66241500 -3.20583800 2.44589000 L

H-H_ -1 -0.04939700 -4.76957200 2.03551500 L

N-N_R -1 -1.06834500 -1.79067400 1.48412000 L

C-C_R -1 -2.08389800 -1.08361100 0.91905300 L

O-O_R -1 -2.10812700 0.15593400 0.91466600 L

C-C_3 -1 -6.27974800 -1.17612100 -1.64804200 H

H-H_ -1 -6.88778100 -1.86139000 -2.24017900 H

C-C_3 -1 -4.79000400 -1.55312400 -1.67478300 H

H-H_ -1 -4.73328800 -2.63703200 -1.78526200 H

H-H_ -1 -4.27765700 -1.10534100 -2.52358800 H

O-O_3 -1 -6.52305100 0.16903500 -2.09371400 H

P-P_3+5-1 -6.08451000 0.73916100 -3.56325300 H

O-O_2- -1 -7.21349700 1.56783200 -4.14051100 H

O-O_2 -1 -5.49296700 -0.35295300 -4.43419400 H

O-O_3 -1 -4.85151000 1.72917800 -3.11300700 H

C-C_3 -1 -4.90911900 2.51085000 -1.91290200 H

H-H_ -1 -4.99513100 1.86131500 -1.03829200 H

H-H_ -1 -5.78271500 3.17569300 -1.94099900 H

C-C_3 -1 -3.64519100 3.35128100 -1.79970800 H

H-H_ -1 -3.85706600 4.17949400 -1.11249600 H

O-O_3 -1 -2.56217600 2.57866300 -1.24251300 H

C-C_3 -1 -1.46047700 2.51539400 -2.16799500 H

H-H_ -1 -0.68898000 3.23086500 -1.85790100 H

N-N_R -1 -0.87460900 1.14366100 -2.06627500 L H-H_ 102 0.

C-C_R -1 -1.40813500 -0.04775600 -2.49042900 L

H-H_ -1 -2.35202800 -0.06032400 -3.01577300 L

N-N_R -1 -0.69463800 -1.09787900 -2.16632200 L

C-C_R -1 0.40915900 -0.56141900 -1.51311000 L

C-C_R -1 1.53428600 -1.20273600 -0.93064300 L

O-O_R -1 1.79228300 -2.40345100 -0.87103700 L

N-N_R -1 2.41945700 -0.27225200 -0.37222200 L

H-H_ -1 3.24549400 -0.62852800 0.06505400 L

C-C_R -1 2.24324400 1.09655800 -0.37674900 L

N-N_R -1 3.20789600 1.81467600 0.21262100 L

H-H_ -1 4.03850000 1.46147100 0.66627000 L

H-H_ -1 3.08298300 2.81751000 0.22173100 L

N-N_R -1 1.18932900 1.69530500 -0.92404200 L

C-C_R -1 0.31927300 0.80448500 -1.47171200 L

C-C_3 -1 -3.15829800 3.89387000 -3.14827000 H

H-H_ -1 -3.95679000 3.91309000 -3.89120300 H

C-C_3 -1 -2.04121200 2.90261500 -3.52492400 H

H-H_ -1 -2.50943000 2.05331300 -4.02877000 H

H-H_ -1 -1.31130600 3.34361300 -4.20133600 H

O-O_3 -1 -2.70284600 5.24760300 -2.92891000 H

P-P_3+5-1 -1.95490100 6.10863500 -4.10573300 H

O-O_2- -1 -2.39690100 7.55115600 -3.94538000 H

O-O_2 -1 -2.06345300 5.43462100 -5.45949700 H

O-O_3 -1 -0.38999700 5.98678100 -3.64121600 H

C-C_3 -1 -0.00685100 6.22646800 -2.27963200 H

H-H_ -1 -0.40745500 5.44331400 -1.63073600 H

H-H_ -1 -0.39026600 7.19879200 -1.94601200 H

C-C_3 -1 1.51284200 6.23399200 -2.21234500 H

H-H_ -1 1.82027600 6.75800300 -1.30014200 H

O-O_3 -1 2.00655300 4.88277600 -2.15144500 H

C-C_3 -1 2.88527200 4.62415800 -3.25860800 H

H-H_ -1 3.92720200 4.72744700 -2.92791200 H

N-N_R -1 2.66208600 3.20623400 -3.63706300 L H-H_ 135 0.

C-C_R -1 1.63614700 2.61608400 -4.33693900 L

H-H_ -1 0.86149800 3.23337800 -4.76800100 L

N-N_R -1 1.73551700 1.32546700 -4.44938400 L

C-C_R -1 2.92080600 1.01998500 -3.79130900 L

C-C_R -1 3.58834100 -0.18794300 -3.55809400 L

N-N_R -1 3.13636300 -1.37644300 -3.98241000 L

H-H_ -1 3.66104200 -2.21607000 -3.78451700 L

H-H_ -1 2.26994500 -1.43217300 -4.49979300 L

N-N_R -1 4.73941000 -0.13591000 -2.87375500 L

C-C_R -1 5.18254800 1.04515900 -2.45563500 L

H-H_ -1 6.10742600 1.10958900 -1.90219800 L

N-N_R -1 4.64583100 2.24125300 -2.61352400 L

C-C_R -1 3.48988400 2.16001300 -3.30478700 L

C-C_3 -1 2.16305700 6.88673200 -3.44640900 H

H-H_ -1 1.47364600 7.54991100 -3.96907300 H

C-C_3 -1 2.55533800 5.67664600 -4.30788600 H

H-H_ -1 1.68365500 5.40704500 -4.90834400 H

H-H_ -1 3.38320100 5.90031500 -4.98010300 H

O-O_3 -1 3.27038200 7.67280800 -2.96260300 H

P-P_3+5-1 4.40151700 8.37081800 -3.92360900 H

O-O_2- -1 4.65563300 9.75802400 -3.33544500 H

O-O_2 -1 4.08733700 8.22975200 -5.38820000 H

O-O_3 -1 5.69555100 7.44243300 -3.59630100 H

C-C_3 -1 6.04953800 7.03007200 -2.26728000 H

H-H_ -1 5.26194600 6.39818800 -1.84800800 H

H-H_ -1 6.18555200 7.90903300 -1.62393000 H

C-C_3 -1 7.35374300 6.25366700 -2.36883100 H

H-H_ -1 7.82911500 6.21118300 -1.38173400 H

O-O_3 -1 7.07531900 4.90391400 -2.80024400 H

C-C_3 -1 7.76671000 4.62412900 -4.02881100 H

H-H_ -1 8.68826300 4.07136300 -3.80213000 H

N-N_R -1 6.89320000 3.70180700 -4.83127300 L H-H_ 167 0.

C-C_R -1 5.76200300 3.95320400 -5.58049400 L

H-H_ -1 5.42163400 4.97099000 -5.70230400 L

N-N_R -1 5.22122100 2.89720200 -6.11303500 L

C-C_R -1 6.05694700 1.85780900 -5.72202300 L

C-C_R -1 6.02649700 0.47948300 -5.96539500 L

N-N_R -1 5.07884900 -0.12136400 -6.69724300 L

H-H_ -1 5.10849800 -1.12104100 -6.83910400 L

H-H_ -1 4.33401000 0.42413100 -7.10670400 L

N-N_R -1 7.00550300 -0.26326500 -5.43131300 L

C-C_R -1 7.94390000 0.33914300 -4.70555100 L

H-H_ -1 8.74132900 -0.24100100 -4.26598000 L

N-N_R -1 8.07558600 1.61921400 -4.41054000 L

C-C_R -1 7.07790700 2.34306300 -4.96028300 L

C-C_3 -1 8.30487300 6.88764400 -3.39976400 H

H-H_ -1 8.01695300 7.91482000 -3.62544000 H

C-C_3 -1 8.11016500 5.98453700 -4.62715400 H

H-H_ -1 7.27709000 6.40065200 -5.19696600 H

H-H_ -1 9.00253200 5.96859100 -5.24987800 H

O-O_3 -1 9.63301000 6.91904700 -2.85064200 H

P-P_3+5-1 10.94656600 7.28228000 -3.80180400 H

O-O_2- -1 11.96615500 7.90997900 -2.87480200 H

O-O_2 -1 10.53899700 7.96122500 -5.08781400 H

O-O_3 -1 11.41648100 5.75406200 -4.20473900 H

C-C_3 -1 11.53416200 4.84109700 -3.11217000 H

H-H_ -1 10.56337000 4.48151200 -2.84169700 H

H-H_ -1 11.97516400 5.34125600 -2.27535300 H

C-C_3 -1 7.31242600 -8.69434400 -10.28278400 L

H-H_ -1 6.92498400 -9.39290100 -10.99470100 L

O-O_2 -1 8.01532700 -9.39453600 -9.26697800 L

P-P_3+q-1 7.33931000 -9.38765500 -8.21633200 L

O-O_2- -1 7.77969500 -10.79684800 -8.32181000 L

O-O_2 -1 5.88902300 -9.14113700 -8.05677400 L

O-O_2 -1 8.19783600 -8.76148700 -7.02120900 L

C-C_3 -1 9.58385400 -8.44499200 -7.24760500 L

H-H_ -1 9.66430800 -7.71384500 -8.05271800 L

H-H_ -1 10.12301100 -9.35083100 -7.52510600 L

C-C_3 -1 10.19923200 -7.86507800 -5.98400300 L

H-H_ -1 11.28439500 -7.95833400 -6.03152600 L

O-O_3 -1 9.95592000 -6.42588400 -5.95816400 L

C-C_3 -1 8.94458900 -6.12022300 -5.00895600 L

H-H_ -1 9.34821900 -5.44981300 -4.25030800 L

N-N_R -1 7.85666000 -5.27393100 -5.67326100 L

C-C_R -1 6.85119700 -5.81138500 -6.43284000 L

H-H_ -1 6.80225500 -6.88018900 -6.58585200 L

C-C_R -1 5.88632000 -5.04199400 -6.95382500 L

C-C_3 -1 4.78142700 -5.62117000 -7.78686700 L

H-H_ -1 4.22441400 -6.34920000 -7.19720200 L

H-H_ -1 4.11045000 -4.82318400 -8.10672400 L

H-H_ -1 5.20514900 -6.11127100 -8.66290000 L

C-C_R -1 5.87675700 -3.61946700 -6.72941100 L

O-O_R -1 5.03514700 -2.83541100 -7.16573800 L

N-N_R -1 6.92512200 -3.16499400 -5.95432000 L

H-H_ -1 6.96089700 -2.09328900 -5.75691100 L

C-C_R -1 7.93141000 -3.93243000 -5.40535400 L

O-O_R -1 8.82008400 -3.43733800 -4.73092500 L

C-C_3 -1 9.61349100 -8.37451600 -4.66486000 L

H-H_ -1 9.25539300 -9.39543700 -4.79580300 L

C-C_3 -1 8.42241800 -7.44106500 -4.44099000 L

H-H_ -1 7.50367000 -8.02604600 -4.40047800 L

H-H_ -1 8.55190700 -6.90370600 -3.50094000 L

O-O_2 -1 10.44933600 -8.27870600 -3.52138800 L

P-P_3+q-1 9.74649300 -8.25327500 -2.47344600 L

O-O_2- -1 10.82200700 -9.21056900 -2.13230800 L

O-O_2 -1 8.35055900 -8.71494400 -2.30828200 L

O-O_2 -1 10.05845800 -6.93959800 -1.61565600 L

C-C_3 -1 11.13437400 -6.07487200 -2.02677900 L

H-H_ -1 10.93879000 -5.70390600 -3.03300800 L

H-H_ -1 12.07073400 -6.63304800 -2.02207800 L

C-C_3 -1 11.24944600 -4.89748000 -1.07338800 L

H-H_ -1 12.24183000 -4.45454800 -1.15982300 L

O-O_3 -1 10.33929700 -3.84354800 -1.51257800 L

C-C_3 -1 9.20894500 -3.78765700 -0.65428800 L

H-H_ -1 9.15184300 -2.80218800 -0.19155000 L

N-N_R -1 7.92834200 -3.85111200 -1.48775000 L

C-C_R -1 7.39870300 -5.02533200 -1.95526900 L

H-H_ -1 7.88940300 -5.96296800 -1.73984900 L

C-C_R -1 6.24473300 -5.04345900 -2.63762300 L

C-C_3 -1 5.65722300 -6.32334700 -3.15348500 L

H-H_ -1 5.46114500 -6.99663900 -2.31878600 L

H-H_ -1 4.72357000 -6.11112500 -3.67535200 L

H-H_ -1 6.35826200 -6.79466300 -3.84223800 L

C-C_R -1 5.52411000 -3.82501000 -2.90016800 L

O-O_R -1 4.46249200 -3.74477900 -3.51504900 L

N-N_R -1 6.12941600 -2.69330600 -2.39332500 L

H-H_ -1 5.62091100 -1.74492300 -2.56718300 L

C-C_R -1 7.31479300 -2.64263200 -1.68824700 L

O-O_R -1 7.77234300 -1.58796400 -1.27924700 L

C-C_3 -1 10.84243900 -5.17246700 0.37606700 L

H-H_ -1 11.03951500 -6.21674700 0.61736800 L

C-C_3 -1 9.33190400 -4.93055600 0.35516300 L

H-H_ -1 8.81309300 -5.84230000 0.65025700 L

H-H_ -1 9.08226100 -4.12936900 1.05183800 L

O-O_2 -1 11.39702700 -4.31596500 1.36374800 L

P-P_3+q-1 10.50267600 -4.40830800 2.26332700 L

O-O_2- -1 11.88682900 -4.59079800 2.75413000 L

O-O_2 -1 9.48426100 -5.37363200 2.73389600 L

O-O_2 -1 10.11909200 -2.89719800 2.62476000 L

C-C_3 -1 10.67521000 -1.82808400 1.83584100 L

H-H_ -1 10.37101900 -1.94613900 0.79671600 L

H-H_ -1 11.76354500 -1.85606400 1.90070500 L

C-C_3 -1 10.17887800 -0.48848100 2.35287900 L

H-H_ -1 10.84693800 0.30332200 2.01295600 L

O-O_3 -1 8.89899600 -0.18311000 1.72048200 L

C-C_3 -1 7.84094500 -0.37038000 2.64920300 L

H-H_ -1 7.30448400 0.56982000 2.78422600 L

N-N_R -1 6.82732900 -1.26596700 2.02289000 L

C-C_R -1 6.98984700 -2.61640400 2.02136800 L

H-H_ -1 7.86340200 -3.05551100 2.47974000 L

C-C_R -1 6.06403900 -3.43170600 1.44918700 L

H-H_ -1 6.20965900 -4.51176400 1.45774700 L

C-C_R -1 4.92507900 -2.80197300 0.85567600 L

N-N_R -1 3.98750600 -3.53384100 0.28278900 L

H-H_ -1 3.19946700 -3.05166500 -0.12466900 L

H-H_ -1 4.06958900 -4.53976300 0.26030700 L

N-N_R -1 4.77523200 -1.48097700 0.86202600 L

C-C_R -1 5.71296500 -0.68211900 1.43918100 L

O-O_R -1 5.60837200 0.55301400 1.46486000 L

C-C_3 -1 9.88773400 -0.42042900 3.85410100 L

H-H_ -1 10.54545000 -1.10974400 4.38309100 L

C-C_3 -1 8.44500400 -0.91986800 3.94284700 L

H-H_ -1 8.40593800 -1.81153600 4.56853900 L

H-H_ -1 7.81731200 -0.14319000 4.37825000 L

O-O_2 -1 9.92897200 0.86841200 4.44927100 L

P-P_3+q-1 8.94772800 0.72909000 5.51501900 L

O-O_2- -1 10.18212200 1.42760500 5.93638600 L

O-O_2 -1 8.48578800 -0.39873000 6.35626400 L

O-O_2 -1 7.83927900 1.87325000 5.37081400 L

C-C_3 -1 7.88525300 2.74968500 4.22942200 L

H-H_ -1 7.79236300 2.16283200 3.31545700 L

H-H_ -1 8.83439700 3.28585000 4.22039300 L

C-C_3 -1 6.74562000 3.75324200 4.29687500 L

H-H_ -1 6.97461100 4.60877300 3.66218300 L

O-O_3 -1 5.55669100 3.15777200 3.69351000 L

C-C_3 -1 4.63045500 2.78835500 4.70421500 L

H-H_ -1 3.69308600 3.32470700 4.55211200 L

N-N_R -1 4.25875300 1.36191200 4.49225100 L

C-C_R -1 4.93586800 0.21932400 4.86617500 L

H-H_ -1 5.87637100 0.29680900 5.39204900 L

N-N_R -1 4.33434300 -0.89352200 4.52430300 L

C-C_R -1 3.18074300 -0.46341800 3.87850200 L

C-C_R -1 2.12730500 -1.20813600 3.28499400 L

O-O_R -1 1.99531000 -2.42796700 3.20456400 L

N-N_R -1 1.15030400 -0.36504900 2.74106700 L

H-H_ -1 0.36537600 -0.79772100 2.29698700 L

C-C_R -1 1.18393600 1.01434500 2.76831300 L

N-N_R -1 0.14936200 1.63840000 2.19056300 L

H-H_ -1 -0.64038100 1.20864500 1.73012000 L

H-H_ -1 0.17009000 2.64878700 2.19788500 L

N-N_R -1 2.17045800 1.70984900 3.32675500 L

C-C_R -1 3.12822900 0.90493400 3.86072300 L

C-C_3 -1 6.29674300 4.15634600 5.70310700 L

H-H_ -1 7.14458400 4.09952200 6.38625900 L

C-C_3 -1 5.28072200 3.06892700 6.06025000 L

H-H_ -1 5.61463800 2.53789100 6.95117500 L

H-H_ -1 4.31082700 3.52648800 6.25178400 L

O-O_2 -1 5.63914900 5.40889700 5.82449200 L

P-P_3+q-1 4.74929300 5.11942000 6.96196500 L

O-O_2- -1 5.44218100 6.42351700 7.05298000 L

O-O_2 -1 4.79845300 4.24156800 8.15397600 L

O-O_2 -1 3.25036500 5.46272200 6.52285900 L

C-C_3 -1 2.99204800 5.83954700 5.15626000 L

H-H_ -1 3.29212600 5.02771900 4.49306200 L

H-H_ -1 3.56137900 6.73647200 4.91251500 L

C-C_3 -1 1.51111300 6.11958300 4.96519100 L

H-H_ -1 1.36736600 6.72757000 4.07150100 L

O-O_3 -1 0.82824700 4.86386600 4.66603100 L

C-C_3 -1 0.08951200 4.42916300 5.79870600 L

H-H_ -1 -0.96656800 4.35937800 5.54035600 L

N-N_R -1 0.47474800 3.01970800 6.09028800 L

C-C_R -1 1.60688600 2.73022900 6.78678000 L

H-H_ -1 2.24246900 3.52831500 7.13910500 L

C-C_R -1 1.95644100 1.44331000 7.05123900 L

H-H_ -1 2.86671400 1.22950400 7.61157900 L

C-C_R -1 1.08203200 0.42164400 6.56363600 L

N-N_R -1 1.36016300 -0.84958300 6.78437700 L

H-H_ -1 0.71791700 -1.53985300 6.42382300 L

H-H_ -1 2.19154800 -1.10611500 7.29827400 L

N-N_R -1 -0.02252300 0.71448700 5.88337100 L

C-C_R -1 -0.35467400 2.00815700 5.62850300 L

O-O_R -1 -1.37553600 2.32089400 4.99804900 L

C-C_3 -1 0.77451900 6.67155300 6.18814600 L

H-H_ -1 1.46640700 7.25506200 6.79558400 L

C-C_3 -1 0.37504800 5.40351100 6.94461100 L

H-H_ -1 0.82377300 5.41535200 7.93762000 L

H-H_ -1 -0.71005900 5.36220800 7.03697800 L

O-O_2 -1 -0.41290800 7.40701000 5.93324900 L

P-P_3+q-1 -1.19533800 7.11864100 7.12614300 L

O-O_2- -1 -1.23942700 8.54190300 6.71966700 L

O-O_2 -1 -0.86284000 6.82743400 8.53788200 L

O-O_2 -1 -2.61283300 6.51085500 6.69782800 L

C-C_3 -1 -2.86340900 6.23834500 5.30691800 L

H-H_ -1 -2.13366300 5.51543600 4.94008000 L

H-H_ -1 -2.77906100 7.16196200 4.73356500 L

C-C_3 -1 -4.26155400 5.66832000 5.13205100 L

H-H_ -1 -4.58003200 5.79389800 4.09745500 L

O-O_3 -1 -4.20947000 4.22243100 5.32586400 L

C-C_3 -1 -4.76666200 3.87910100 6.58572000 L

H-H_ -1 -5.61898200 3.21513900 6.43840900 L

N-N_R -1 -3.78054700 3.02994900 7.31164900 L

C-C_R -1 -2.67285100 3.40978500 8.04150400 L

H-H_ -1 -2.42643600 4.45719500 8.14305200 L

N-N_R -1 -2.00523800 2.40878100 8.55752000 L

C-C_R -1 -2.71339700 1.28690400 8.14342700 L

C-C_R -1 -2.47886600 -0.09294100 8.38803000 L

O-O_R -1 -1.57315900 -0.61717900 9.03544400 L

N-N_R -1 -3.45085000 -0.89227300 7.77578700 L

H-H_ -1 -3.36962700 -1.88245400 7.88965500 L

C-C_R -1 -4.51173700 -0.42439100 7.02758600 L

N-N_R -1 -5.33326300 -1.35605400 6.52520100 L

H-H_ -1 -5.25023100 -2.35708400 6.62652100 L

H-H_ -1 -6.10700700 -1.02212300 5.96895600 L

N-N_R -1 -4.73153600 0.86790300 6.80122100 L

C-C_R -1 -3.79540500 1.65964900 7.39017800 L

C-C_3 -1 -5.30673200 6.15001600 6.14083400 L

H-H_ -1 -5.06009900 7.16047300 6.46648000 L

C-C_3 -1 -5.12291800 5.17923400 7.30960500 L

H-H_ -1 -4.85231200 5.73630200 8.20553300 L

H-H_ -1 -6.05327100 4.63953500 7.48417800 L

O-O_3 -1 -6.66499600 6.07175200 5.73329800 L

H-H_ -1 -7.30147300 6.37956300 6.38227400 L

Na-Na -1 -0.00573400 9.38712400 9.51126900 L

Na-Na -1 5.79054400 7.71579500 9.29726600 L

Na-Na -1 11.55794500 1.84741700 7.66204200 L

Na-Na -1 13.33441900 -4.33316200 5.76421600 L

Na-Na -1 11.23792000 -11.12351600 -1.11056100 L

N-N_3 0 -9.39356300 -10.12265100 2.69662900 H

C-C_3 0 -8.95686300 -9.56408200 1.41096300 H

C-C_3 0 -9.97638800 -8.64448100 0.80392200 H

C-C_3 0 -9.50990600 -8.11659100 -0.53182000 H

C-C_3 0 -9.70314900 -9.08098900 -1.67688300 H

C-C_3 0 -9.15040900 -8.51453900 -2.96370700 H

C-C_3 0 -9.57080900 -9.25209700 -4.20243800 H

N-N_3 0 -11.02327800 -9.15953900 -4.42436800 H

N-N_3 0 -12.68337300 -7.44088300 -2.74921900 H

N-N_3 0 -10.91571700 -7.06924800 -6.41703300 H

Pt-Pt4+ 0 -11.79591100 -7.26152600 -4.58350900 H

N-N_3 0 -12.57146200 -5.37078300 -4.73573500 H

C-C_3 0 -11.95738100 -4.35106500 -3.87321100 H

C-C_3 0 -10.53186900 -4.09557500 -4.27017500 H

C-C_3 0 -9.86213100 -3.04285600 -3.41764900 H

C-C_3 0 -10.53446000 -1.68947400 -3.46460300 H

C-C_3 0 -9.67816600 -0.62636300 -2.82604100 H

C-C_3 0 -10.26938000 0.75567700 -2.91839200 H

N-N_3 0 -9.33942500 1.75203500 -2.38587800 H

N-N_3 0 -8.81444200 3.72883200 -4.37033400 H

N-N_3 0 -10.80874300 3.69646200 -0.80430100 H

Pt-Pt4+ 0 -9.81084000 3.72438700 -2.59054100 H

N-N_3 0 -10.19555100 5.72957500 -2.83239800 H

C-C_3 0 -11.20268600 6.07430600 -3.84450500 H

C-C_3 0 -11.17345900 7.53274200 -4.21917600 H

C-C_3 0 -9.93947800 7.92675200 -4.99628000 H

C-C_3 0 -9.95584500 9.37801600 -5.41236700 H

C-C_3 0 -8.84830100 9.74644000 -6.37576300 H

C-C_3 0 -7.45671500 9.44912700 -5.87898900 H

N-N_3 0 3.78616200 10.93495700 -1.24569900 H

C-C_3 0 2.91700900 10.12535800 -0.38069200 H

C-C_3 0 1.68508200 9.67583500 -1.11503900 H

C-C_3 0 0.77469100 10.78729700 -1.56933300 H

C-C_3 0 -0.39661700 10.26978500 -2.36840400 H

C-C_3 0 -1.30996500 11.38913600 -2.79744700 H

C-C_3 0 -2.43643400 10.98007000 -3.70615800 H

N-N_3 0 -3.34633600 10.00167700 -3.09509400 H

N-N_3 0 -4.92982900 8.21629200 -4.70883700 H

N-N_3 0 -7.14581900 10.16009600 -4.63625600 H

Pt-Pt4+ 0 -5.23684700 10.04393400 -3.87341600 H

N-N_3 0 -5.52173200 11.88622600 -3.01310200 H

H-H_ 0 -8.50615700 -10.32669300 3.27735800 H

H-H_ 0 -9.94554900 -10.96802100 2.58107700 H

H-H_ 0 -8.71328800 -10.39993500 0.75625600 H

H-H_ 0 -8.02689100 -9.02610100 1.60509000 H

H-H_ 0 -10.93407000 -9.16483100 0.69325700 H

H-H_ 0 -10.14671400 -7.80971200 1.49159100 H

H-H_ 0 -8.45115700 -7.83560100 -0.47648600 H

H-H_ 0 -10.01452000 -7.17262100 -0.75834200 H

H-H_ 0 -9.22713300 -10.04493300 -1.46922000 H

H-H_ 0 -10.77454700 -9.30604500 -1.76407700 H

H-H_ 0 -8.05908400 -8.51918200 -2.91591400 H

H-H_ 0 -9.44211500 -7.46235300 -3.06008000 H

H-H_ 0 -9.07330200 -8.83128700 -5.07612000 H

H-H_ 0 -9.29664200 -10.30809700 -4.14168400 H

H-H_ 0 -11.26571500 -9.68030300 -5.26348800 H

H-H_ 0 -13.30455000 -8.24411800 -2.69878500 H

H-H_ 0 -12.00015500 -7.53855100 -2.00183900 H

H-H_ 0 -10.44793900 -7.91034300 -6.74134800 H

H-H_ 0 -10.20950400 -6.33804300 -6.42347500 H

H-H_ 0 -12.52281100 -5.03746900 -5.69562900 H

H-H_ 0 -12.55213500 -3.43878000 -3.94605000 H

H-H_ 0 -12.00634500 -4.69661700 -2.83864500 H

H-H_ 0 -10.50609800 -3.78414600 -5.32189100 H

H-H_ 0 -9.97186000 -5.03322900 -4.19434600 H

H-H_ 0 -8.83134600 -2.93089400 -3.77133300 H

H-H_ 0 -9.78351900 -3.40916500 -2.38732600 H

H-H_ 0 -10.73189500 -1.41427200 -4.50813300 H

H-H_ 0 -11.50817500 -1.72249900 -2.96451900 H

H-H_ 0 -8.69875700 -0.61444200 -3.31496700 H

H-H_ 0 -9.49448700 -0.87859400 -1.77420300 H

H-H_ 0 -10.46710600 1.01261600 -3.96081900 H

H-H_ 0 -11.21802400 0.82224900 -2.38266700 H

H-H_ 0 -8.45054200 1.62127800 -2.89338400 H

H-H_ 0 -8.34768200 4.60380800 -4.58243900 H

H-H_ 0 -8.08797000 2.99216200 -4.37982800 H

H-H_ 0 -11.71170400 4.15786800 -0.86039700 H

H-H_ 0 -10.27933100 4.16119400 -0.07236700 H

H-H_ 0 -9.31425800 6.17916700 -3.06826500 H

H-H_ 0 -11.02028700 5.45839300 -4.72667600 H

H-H_ 0 -12.17414200 5.78549100 -3.44651900 H

H-H_ 0 -12.05724900 7.73880700 -4.82899400 H

H-H_ 0 -11.27008000 8.15108500 -3.31972800 H

H-H_ 0 -9.03632500 7.72789200 -4.40514300 H

H-H_ 0 -9.86103300 7.29429200 -5.88848300 H

H-H_ 0 -9.92718400 10.01676000 -4.52197900 H

H-H_ 0 -10.91316800 9.59880600 -5.89237000 H

H-H_ 0 -8.98419500 9.19578100 -7.31066600 H

H-H_ 0 -8.91932900 10.80785300 -6.63175900 H

H-H_ 0 -6.71843300 9.74410000 -6.62424500 H

H-H_ 0 -7.33617900 8.38116000 -5.69666700 H

H-H_ 0 4.61729800 11.23655800 -0.74446900 H

H-H_ 0 4.11855900 10.39144400 -2.13005600 H

H-H_ 0 3.50422800 9.26655700 -0.05786100 H

H-H_ 0 2.66631700 10.71790600 0.49995300 H

H-H_ 0 1.13407800 9.00671700 -0.44682200 H

H-H_ 0 2.00002500 9.07621800 -1.97148400 H

H-H_ 0 0.41175900 11.35360700 -0.70435900 H

H-H_ 0 1.31644300 11.50168600 -2.20056800 H

H-H_ 0 -0.02905200 9.73454000 -3.25206000 H

H-H_ 0 -0.94619400 9.53306600 -1.77039900 H

H-H_ 0 -0.72500600 12.14919300 -3.32406500 H

H-H_ 0 -1.71977400 11.88640300 -1.91006400 H

H-H_ 0 -2.05623900 10.53036100 -4.62501700 H

H-H_ 0 -3.00772500 11.86173000 -3.99720000 H

H-H_ 0 -2.93248700 9.06388200 -3.20084900 H

H-H_ 0 -3.97436000 7.86926600 -4.49900200 H

H-H_ 0 -5.02815900 8.23385200 -5.71870100 H

H-H_ 0 -7.77996100 9.84295800 -3.90626700 H

H-H_ 0 -4.77909800 12.15629900 -2.37580400 H

H-H_ 0 -5.57551700 12.62515800 -3.70891500 H

H-H_ 0 3.31264400 11.77953600 -1.55600800 H

H-H_ 0 -3.40649600 10.16704000 -2.09285900 H

H-H_ 0 -5.58340200 7.52569800 -4.35317100 H

H-H_ 0 -6.37706200 11.93125700 -2.46664600 H

H-H_ 0 -7.37662800 11.14072900 -4.78034500 H

H-H_ 0 -10.46222800 6.16252400 -1.95223400 H

H-H_ 0 -11.00072300 2.75849700 -0.46647100 H

H-H_ 0 -9.12672600 1.52691100 -1.41755800 H

H-H_ 0 -9.44452600 3.53994600 -5.14423800 H

H-H_ 0 -13.56988200 -5.41604800 -4.54726300 H

H-H_ 0 -13.25737700 -6.64220000 -2.49545000 H

H-H_ 0 -11.59066000 -6.83923900 -7.14122000 H

H-H_ 0 -11.49578000 -9.67212600 -3.68482800 H

H-H_ 0 -9.97122800 -9.46224900 3.21241800 H

Na-Na -1 5.00610900 -12.81666000 -8.60397600 L

H-H_ 0 12.16640700 4.01837300 -3.43758500 H

H-H_ 0 8.07639300 -8.14421300 -10.87232100 L

H-H_ 0 6.57113300 -7.88464000 -10.02016300 L

*Model C*

0 1 -4 1 -4 1

C-C_3 0 -13.83564700 3.29896800 0.13611500 L

H-H_ 0 -13.85520300 2.22584700 0.42899200 L

C-C_3 0 -11.69620500 3.55049300 0.99775100 L

C-C_3 0 -10.39344600 4.36037600 0.85427800 L H-H_ 9 0.0000

C-C_3 0 -9.52799900 3.76792500 -0.28256800 L

C-C_3 0 -9.33379000 2.25675800 -0.06904400 L

C-C_3 0 -10.72387800 1.59597800 0.12182000 L H-H_ 8 0.0000

C-C_3 0 -10.61812500 0.10414900 0.37174300 H

N-N_3 0 -10.72541100 5.75427600 0.61551800 H

O-O_3 0 -12.52343100 3.73648800 -0.14136100 L

O-O_3 0 -8.27218400 4.39447800 -0.30481000 L

O-O_3 0 -11.41223200 2.17655400 1.21977300 L

O-O_3 0 -9.83564700 -0.11439200 1.53693400 H

S-S_3+6 0 -9.97685200 6.84247200 1.63454100 H

O-O_2 0 -10.47920800 6.51427200 2.96515000 H

O-O_2 0 -8.52545400 6.69910200 1.53793900 H

O-O_2 0 -10.44914000 8.12476000 1.11857000 H

S-S_3+6 0 -10.23095100 -1.31747500 2.46525300 H

O-O_2 0 -10.37309400 -2.49242300 1.59743100 H

O-O_2 0 -9.13043600 -1.39676900 3.39544500 H

O-O_2 0 -11.51882100 -0.98211500 3.07541100 H

H-H_ 0 -12.20134200 3.94272200 1.91181000 L

H-H_ 0 -9.82948400 4.23777500 1.80857700 L

H-H_ 0 -10.04695700 3.91328300 -1.26167900 L

H-H_ 0 -8.74398600 2.12143700 0.87088500 L

H-H_ 0 -11.31532800 1.71269900 -0.81579600 L

H-H_ 0 -11.62316600 -0.29914600 0.51916800 H

H-H_ 0 -10.16476400 -0.40225900 -0.48188800 H

H-H_ 0 -10.64858800 6.03558900 -0.35220900 H

H-H_ 0 -8.35163900 5.18863300 -0.89453900 L

C-C_3 0 -7.51585000 1.00569800 -0.90764900 L H-H_ 32 0.0000

C-C_3 0 -7.02801400 0.27064700 -2.17217200 H

C-C_3 0 -5.50782300 0.23649900 -2.26121700 L H-H_ 32 0.0000

C-C_3 0 -4.83814300 1.62470600 -2.03546700 L

C-C_3 0 -5.82679800 2.58241100 -1.33455800 L H-H_ 36 0.0000

C-C_R 0 -5.09904800 3.77972400 -0.71730500 H

O-O_3 0 -8.70083400 1.72120800 -1.22356100 L

O-O_3 0 -7.47703000 -1.07473200 -2.15769300 H

O-O_3 0 -5.10651200 -0.32245800 -3.48726300 L

O-O_3 0 -6.53878700 1.87429800 -0.33731600 L

O-O_R 0 -4.57980100 4.58493100 -1.50942400 H

O-O_R 0 -5.07718100 3.86805700 0.52211700 H

S-S_3+6 0 -8.65882600 -1.52273300 -3.12630500 H

O-O_2 0 -8.49263900 -2.97856100 -3.08491900 H

O-O_2 0 -9.89431100 -1.07697000 -2.50699400 H

O-O_2 0 -8.39022200 -0.92744100 -4.41881300 H

H-H_ 0 -7.73931700 0.22782200 -0.14281200 L

H-H_ 0 -7.41393000 0.77289400 -3.06365200 H

H-H_ 0 -5.16871500 -0.44904200 -1.45716000 L

H-H_ 0 -4.58761800 2.10341000 -3.01341800 L

H-H_ 0 -6.53628000 2.99966300 -2.08923500 L

H-H_ 0 -5.41825200 0.28045500 -4.21198600 L

Pt-Pt4+2 0 4.47346200 5.39739000 -1.83620800 H

N-N_3 0 6.44947100 4.86757200 -1.95414900 H

N-N_3 0 4.00455200 4.38775000 -3.56334900 H

N-N_3 0 2.52634500 5.98755000 -1.72658700 H

H-H_ 0 6.98591300 5.58870000 -2.42676300 H

H-H_ 0 6.61044100 4.00722000 -2.46738800 H

H-H_ 0 4.76490900 4.44683100 -4.23477400 H

H-H_ 0 3.20373500 4.86331000 -3.99490600 H

H-H_ 0 2.32620900 6.55134900 -0.89656200 H

H-H_ 0 6.85385800 4.74410200 -1.01713100 H

H-H_ 0 1.88877600 5.19032700 -1.72155900 H

H-H_ 0 2.25834100 6.51407800 -2.55357900 H

N-N_3 0 4.86094900 6.41297000 -0.10292400 H

H-H_ 0 5.49363200 5.85501800 0.47521000 H

H-H_ 0 3.98210400 6.48795900 0.41480200 H

C-C_3 0 5.41019800 7.76020500 -0.28546900 H

H-H_ 0 4.92512800 8.21299000 -1.15017900 H

H-H_ 0 6.46849000 7.65852000 -0.53282300 H

C-C_3 0 3.60063600 2.98616700 -3.41521900 H

H-H_ 0 3.21385200 2.63021600 -4.37392900 H

H-H_ 0 2.76184300 2.95815100 -2.71795900 H

C-C_3 0 4.71714400 2.10214800 -2.94741000 H

H-H_ 0 5.15731400 2.51643200 -2.03421100 H

H-H_ 0 5.50796000 2.07485300 -3.70753000 H

C-C_3 0 4.25377100 0.69760500 -2.64717900 H

H-H_ 0 3.60391200 0.33816400 -3.45137100 H

H-H_ 0 3.63370500 0.71785000 -1.74419900 H

C-C_3 0 5.42173000 -0.24628300 -2.44253300 H

H-H_ 0 5.73625800 -0.66234100 -3.40525500 H

H-H_ 0 6.27693900 0.33072600 -2.07811900 H

C-C_3 0 5.13554200 -1.35449100 -1.46016100 H

H-H_ 0 4.85867300 -0.91565600 -0.49578600 H

H-H_ 0 6.03287300 -1.96211500 -1.30950900 H

C-C_3 0 4.00701700 -2.25495900 -1.87013400 H

H-H_ 0 3.10496700 -1.67420000 -2.06940300 H

H-H_ 0 4.25447000 -2.79914100 -2.78524500 H

C-C_3 0 5.19863000 8.63670500 0.92640900 H

H-H_ 0 4.12746100 8.83480600 1.02320400 H

H-H_ 0 5.68547400 9.59727900 0.73376900 H

C-C_3 0 5.70766600 8.04703200 2.22039100 H

H-H_ 0 5.47994600 8.73620400 3.03870300 H

H-H_ 0 5.14851400 7.13153300 2.44927000 H

C-C_3 0 7.18217500 7.72976500 2.21922100 H

H-H_ 0 7.76385800 8.65041500 2.09960500 H

H-H_ 0 7.42271300 7.08687000 1.36654400 H

C-C_3 0 7.58815900 6.99407200 3.47142300 H

H-H_ 0 7.43035500 7.61689900 4.35698100 H

H-H_ 0 6.93784000 6.11982300 3.56955700 H

C-C_3 0 9.01517200 6.51726000 3.41203300 H

H-H_ 0 9.72931400 7.28857700 3.69493600 H

H-H_ 0 9.26712800 6.16108800 2.41285100 H

N-N_3 0 3.69197500 -3.21059400 -0.81145900 H

H-H_ 0 4.50552600 -3.78675600 -0.59776800 H

N-N_3 0 9.19473800 5.36012900 4.30398400 H

H-H_ 0 8.53421000 4.61540100 3.98277100 H

H-H_ 0 8.99081400 5.59087200 5.27384500 H

H-H_ 0 10.14142300 4.99061500 4.26406300 H

H-H_ 0 3.49495100 -2.66204400 0.03735000 H

C-C_3 0 -2.53292300 1.13415600 -1.99928200 L

C-C_3 0 -1.64927200 0.08416600 -1.27175400 L H-H_ 117 0.000

C-C_3 0 -0.91752900 0.71813700 -0.07920100 L

C-C_3 0 -0.17928300 1.98662900 -0.52861600 L

C-C_3 0 -1.23319800 2.91999800 -1.16845100 L H-H_ 116 0.000

C-C_3 0 -0.64296100 4.26502100 -1.55744900 H

N-N_3 0 -2.41764700 -1.04162700 -0.79142400 H

O-O_3 0 -3.68010500 1.46231400 -1.22433900 L

O-O_3 0 -0.03346700 -0.20548500 0.49409300 L

O-O_3 0 -1.79607900 2.30876300 -2.32220400 L

O-O_3 0 0.46964400 4.11596700 -2.42607900 H

S-S_3+6 0 -2.50163300 -2.34770200 -1.78575300 H

O-O_2 0 -2.82734700 -1.98045700 -3.14992300 H

O-O_2 0 -1.17353700 -2.96727500 -1.70225400 H

O-O_2 0 -3.55063800 -3.14441300 -1.14118400 H

S-S_3+6 0 0.32918400 4.35978800 -4.01411000 H

O-O_2 0 1.45848600 5.25305000 -4.28021000 H

O-O_2 0 0.48780100 3.05207800 -4.61571500 H

O-O_2 0 -0.96426500 4.97865400 -4.21611300 H

H-H_ 0 -2.80715000 0.65741000 -2.96701200 L

H-H_ 0 -0.87427500 -0.25791100 -1.99774600 L

H-H_ 0 -1.66419200 0.99581500 0.69879300 L

H-H_ 0 0.55394100 1.70312600 -1.32489100 L

H-H_ 0 -2.03070000 3.14232300 -0.42179700 L

H-H_ 0 -1.40645300 4.87364000 -2.04149800 H

H-H_ 0 -0.28333200 4.78751300 -0.66855300 H

H-H_ 0 -3.26263300 -0.81303300 -0.29334400 H

H-H_ 0 0.59469700 -0.48309700 -0.22177200 L

C-C_3 0 1.83725500 2.59447000 0.52390800 L H-H_ 289 0.000

C-C_3 0 2.42423900 3.52715000 1.60200000 H

C-C_3 0 3.81730600 3.07058700 2.02720900 L H-H_ 289 0.000

C-C_3 0 3.86512000 1.56240000 2.40175000 L

C-C_3 0 2.59332100 0.84429300 1.90501000 L H-H_ 144 0.000

C-C_R 0 2.74033800 -0.67679800 1.98515100 H

O-O_3 0 0.41847400 2.60770200 0.60468700 L

O-O_3 0 2.53330500 4.83535400 1.07318200 H

O-O_3 0 4.25817700 3.83356600 3.12172800 L

O-O_3 0 2.32945500 1.25554800 0.57776700 L

O-O_R 0 2.84366500 -1.17421200 3.10767400 H

O-O_R 0 2.73542900 -1.32249700 0.90805600 H

S-S_3+6 0 1.60148700 6.00120100 1.64477600 H

O-O_2 0 2.18896300 7.12819000 0.91654100 H

O-O_2 0 0.24499700 5.68439900 1.24093500 H

O-O_2 0 1.80268500 6.03793400 3.07782000 H

H-H_ 0 2.16120100 3.01444400 -0.45390400 L

H-H_ 0 1.77620300 3.53454800 2.48281300 H

H-H_ 0 4.49490700 3.24171900 1.15811200 L

H-H_ 0 3.87410500 1.44051500 3.51261100 L

H-H_ 0 1.73097100 1.10429900 2.56459400 L

H-H_ 0 4.57919900 4.70120000 2.76234300 L

C-C_3 0 6.13634400 0.97109000 2.64133200 L

C-C_3 0 7.44165500 1.17515600 1.83046200 L H-H_ 238 0.000

C-C_3 0 7.72786600 -0.05712000 0.95448500 L

C-C_3 0 7.59179100 -1.37807900 1.73407900 L

C-C_3 0 6.27652600 -1.36856000 2.54934300 L H-H_ 257 0.000

C-C_3 0 6.12824200 -2.61106400 3.40658900 H

N-N_3 0 7.35488800 2.34213200 0.97550300 H

O-O_3 0 5.00080600 0.97212600 1.78369900 L

O-O_3 0 9.02299500 0.03379500 0.41967000 L

O-O_3 0 6.19868400 -0.22955600 3.39248400 L

O-O_3 0 7.17345600 -2.65281400 4.35783800 H

S-S_3+6 0 7.79540800 3.78436200 1.63514300 H

O-O_2 0 7.35523000 3.88018200 3.03294300 H

O-O_2 0 9.24370000 3.87355100 1.55627300 H

O-O_2 0 7.09363400 4.74164100 0.76906600 H

S-S_3+6 0 7.16550200 -3.92721900 5.32266700 H

O-O_2 0 8.33525200 -3.68755100 6.14874900 H

O-O_2 0 5.90031100 -3.86766500 6.04271500 H

O-O_2 0 7.27219700 -5.09015500 4.45383600 H

H-H_ 0 6.09574200 1.80587700 3.37425100 L

H-H_ 0 8.28857400 1.28372700 2.54710600 L

H-H_ 0 6.98413200 -0.07527000 0.13045200 L

H-H_ 0 8.45279300 -1.45252000 2.44263800 L

H-H_ 0 5.42332700 -1.39073000 1.83692700 L

H-H_ 0 5.16489400 -2.56438800 3.92221900 H

H-H_ 0 6.13883700 -3.50391100 2.77899000 H

H-H_ 0 6.51444200 2.39183500 0.42079200 H

H-H_ 0 8.96629400 0.60550300 -0.39011000 L

C-C_3 0 8.55128500 -3.39769300 0.96679500 L H-H_ 263 0.000

C-C_3 0 8.28472100 -4.58518800 0.01649100 H

C-C_3 0 9.59163100 -5.22790100 -0.43130400 L H-H_ 263 0.000

C-C_3 0 10.55623700 -4.18717700 -1.06792300 L

C-C_3 0 10.19683300 -2.75975500 -0.58256800 L H-H_ 194 0.000

C-C_R 0 11.34756700 -1.77564700 -0.79628600 H

O-O_3 0 7.53150700 -2.43418900 0.78404600 L

O-O_3 0 7.49997600 -5.57474100 0.65349000 H

O-O_3 0 9.32223000 -6.24537900 -1.36347800 L

O-O_3 0 9.83712800 -2.80551200 0.78607800 L

O-O_R 0 11.55788900 -1.41233000 -1.96653600 H

O-O_R 0 11.97981000 -1.39756000 0.20482300 H

S-S_3+6 0 5.94144300 -5.68317900 0.34319400 H

O-O_2 0 5.64524500 -7.05320400 0.69405100 H

O-O_2 0 5.28541700 -4.68235500 1.16823900 H

O-O_2 0 5.78933300 -5.37354200 -1.08447200 H

H-H_ 0 8.51564800 -3.79072100 2.00759900 L

H-H_ 0 7.75453900 -4.23818500 -0.87570300 H

H-H_ 0 10.08786100 -5.66201900 0.47128600 L

H-H_ 0 10.43035700 -4.18122800 -2.17831400 L

H-H_ 0 9.34131600 -2.36862500 -1.18512600 L

H-H_ 0 9.09496400 -7.06273200 -0.84766700 L

C-C_3 0 12.49980400 -5.40294800 -1.59545100 L

C-C_3 0 13.56972100 -6.22516300 -0.86494700 L

C-C_3 0 14.04568700 -3.82648400 -2.41847500 L

O-O_3 0 11.89183700 -4.49436500 -0.68851700 L

O-O_3 0 13.00617200 -4.72932700 -2.74056100 L

H-H_ 0 11.76797600 -6.15705800 -1.96237000 L

H-H_ 0 14.12756900 -6.85515800 -1.58984200 L

Pt-Pt4+2 0 2.09239000 -4.35191400 -1.31698000 H

N-N_3 0 3.32617800 -5.42861400 -2.55329700 H

N-N_3 0 0.40398100 -5.29250800 -2.03281700 H

N-N_3 0 0.96154100 -3.32238900 0.02466200 H

H-H_ 0 3.43999900 -5.00256200 -3.46760700 H

H-H_ 0 2.99892900 -6.37571200 -2.71281800 H

H-H_ 0 0.58711800 -5.59105200 -2.98734600 H

H-H_ 0 -0.28356000 -4.53527400 -2.11148100 H

H-H_ 0 0.67834000 -3.93177800 0.78489900 H

H-H_ 0 4.25547400 -5.50535600 -2.12016800 H

H-H_ 0 0.10898300 -2.96768400 -0.41963000 H

H-H_ 0 1.49364300 -2.54633400 0.44470300 H

C-C_3 0 -0.21682300 -6.40726200 -1.31696100 H

H-H_ 0 -0.51778200 -6.04098000 -0.33301900 H

H-H_ 0 0.53501200 -7.18081800 -1.16149700 H

C-C_3 0 -1.41162400 -6.95487500 -2.06399400 H

H-H_ 0 -1.81558200 -7.79456900 -1.49040700 H

H-H_ 0 -1.07897900 -7.37014000 -3.02307500 H

C-C_3 0 -2.48842400 -5.92122100 -2.28915700 H

H-H_ 0 -2.09272100 -5.08818400 -2.87887700 H

H-H_ 0 -2.76684900 -5.47869200 -1.32739900 H

C-C_3 0 -3.71979500 -6.42982000 -3.00330000 H

H-H_ 0 -3.44295100 -6.75649100 -4.00963500 H

H-H_ 0 -4.11321300 -7.31740200 -2.49238400 H

C-C_3 0 -4.80025100 -5.36592500 -3.08087600 H

H-H_ 0 -5.50945800 -5.58663900 -3.88243900 H

H-H_ 0 -4.33464400 -4.40305900 -3.31801700 H

C-C_3 0 -5.53434600 -5.24502600 -1.76684600 H

H-H_ 0 -6.30198900 -6.01510200 -1.67695800 H

H-H_ 0 -4.83135600 -5.39804000 -0.94675000 H

N-N_3 0 -6.15949900 -3.94090800 -1.54982700 H

H-H_ 0 -6.85630700 -3.72527300 -2.26762100 H

H-H_ 0 -5.41839700 -3.24391500 -1.64304900 H

H-H_ 0 13.08361400 -6.88847800 -0.11812600 L

H-H_ 0 14.28140900 -5.56531800 -0.32539600 L

H-H_ 0 14.15736900 -3.10653900 -3.25467500 L

H-H_ 0 15.00923800 -4.36970700 -2.31894000 L

H-H_ 0 13.84345000 -3.23751100 -1.49832400 L

Pt-Pt4+2 0 -6.97532000 -3.77367800 0.32054600 H

N-N_3 0 -5.09171500 -3.65998100 1.11699200 H

N-N_3 0 -7.81053800 -3.63820500 2.17702500 H

N-N_3 0 -8.85977600 -3.87090900 -0.45397300 H

H-H_ 0 -4.81576400 -4.53754700 1.54676300 H

H-H_ 0 -5.02462300 -2.94830200 1.83711400 H

H-H_ 0 -8.80688700 -3.47319400 2.02660700 H

H-H_ 0 -7.52959100 -2.78252200 2.65244300 H

H-H_ 0 -8.87843700 -3.60275600 -1.44512000 H

H-H_ 0 -4.38660400 -3.43037900 0.40584400 H

H-H_ 0 -9.49941300 -3.25455400 0.05212800 H

H-H_ 0 -9.23252800 -4.81247600 -0.38152200 H

C-C_3 0 -7.66819300 -4.75949900 3.10920500 H

H-H_ 0 -6.65389100 -4.74973700 3.50861100 H

H-H_ 0 -7.79416000 -5.68887300 2.55098100 H

C-C_3 0 -8.70420000 -4.64711400 4.20179400 H

H-H_ 0 -8.69003800 -3.62132100 4.58636400 H

H-H_ 0 -8.43659200 -5.30140500 5.03440100 H

C-C_3 0 -10.09304600 -4.98565900 3.69654300 H

H-H_ 0 -10.21999400 -6.07328000 3.68598300 H

H-H_ 0 -10.19601600 -4.66300700 2.65590200 H

C-C_3 0 -11.20401300 -4.35535500 4.50115100 H

H-H_ 0 -11.09864300 -4.64075700 5.55180000 H

H-H_ 0 -11.09716400 -3.26413200 4.47838100 H

C-C_3 0 -12.57839400 -4.77270400 4.02373600 H

H-H_ 0 -13.34472700 -4.46865800 4.74371500 H

H-H_ 0 -12.62066500 -5.86458300 3.98940000 H

C-C_3 0 -12.96455100 -4.27314100 2.65521800 H

H-H_ 0 -12.13970400 -4.33422100 1.94627500 H

H-H_ 0 -13.80151800 -4.84499300 2.25997700 H

N-N_3 0 -13.38807600 -2.86170600 2.68228700 H

H-H_ 0 -14.13776700 -2.71810800 3.35636600 H

H-H_ 0 -13.74998700 -2.57848400 1.77400800 H

H-H_ 0 -12.61694600 -2.18755500 2.90905100 H

H-H_ 0 -14.44459000 3.40462700 -0.78489600 L

H-H_ 0 -14.30728200 3.92626600 0.92424100 L
